# Two-Dimensional IR Spectroscopy as a Window into the Molecular Structure and Dynamics of Electrolytes and Ionic Liquids: A Review

**DOI:** 10.1021/acs.chemrev.6c00440

**Published:** 2026-10-14

**Authors:** Valeria Bonilla, Daniel G. Kuroda

**Affiliations:** Department of Chemistry, 5779 Louisiana State University, Baton Rouge, Louisiana 70803, United States

## Abstract

Two-dimensional infrared (2DIR) spectroscopy has emerged as a powerful tool for probing the structure and picosecond dynamics of complex, condensed-phase systems at the molecular level. In electrolytes and ionic liquids, where physicochemical properties arise from the interplay between solvation and ionic interactions, 2DIR spectroscopy provides unique insights into intermolecular interactions and dynamical processes that are often inaccessible to conventional spectroscopies. This review highlights seminal and recent advances in understanding the behavior of these systems using 2DIR spectroscopy, with particular emphasis on spectral diffusion, vibrational relaxation, and chemical exchange dynamics. The review also describes how complementary information from the vibrational modes of solvents, salts, and external probes enables detailed characterization of ion–ion interactions, hydrogen-bonding networks, and solvation environments, providing a molecular framework of the structure and time evolution of these charged systems. Moreover, particular attention is given to the dynamics of distinct molecular moieties and to the role of specific anions, cations, and solvent environments in governing behavior on femtosecond to picosecond time scales. Finally, the review describes current challenges, including the limited availability of intrinsic vibrational reporters and experimental constraints, and key open questions in electrochemical and energy-relevant systems that remain despite an extensive number of studies in the area.

## Graphical abstract

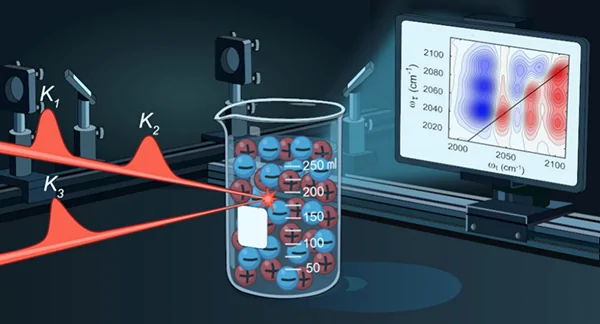

## 1 Introduction

Liquid electrolytes play a central role in modern chemistry as they define ionic transport, charge separation, and electrochemical reactions in a variety of systems, ranging from biological membranes to energy storage devices. Liquid electrolytes are traditionally described as substances that conduct electricity through ion diffusion.^1–3^ This definition encompasses a broad range of salts dissolved in molecular solvents, including both aqueous and organic systems, as well as ionic liquids. The structure of a liquid electrolyte, and consequently its conductivity, stability, and reactivity, are governed by the interplay between ion–solvent and ion–ion interactions, including ion pairing, hydrogen-bonding, and collective solvent fluctuations that govern local organization and dynamical behavior.^4–7^ While significant progress has been made in characterizing the structure of liquid electrolytes at low salt concentrations, establishing a detailed understanding of how these interactions evolve over time and with salt concentration remains a major challenge.^8^

Arrhenius, Debye, and Hückel developed the classical electrolyte theory which established a basis for understanding dilute ionic solutions by quantitatively relating ion dissociation, ionic strength, and activity coefficients to the long-range Coulombic interactions occurring within a continuum solvent framework.^1,9,10^ However, emerging technologies, especially those related to energy storage, require operation under much higher salt concentrations (as in high-concentration electrolytes, or HCEs) and more extreme conditions, such as high voltages and elevated temperatures. Under these environments, the limitations of classical electrolyte theory become evident, as its mean-field approximations break down and strong ion–ion correlations, short-range interactions, and specific solvation effects begin to dominate.^11–13^ These limitations highlight the need for more comprehensive molecular frameworks that explicitly account for the dynamical nature of solvation in concentrated electrolytes.

These challenges are particularly pronounced in a class of electrolytes that has emerged over the past few decades: ionic liquids (ILs).^14,15^ ILs are broadly defined as salts composed entirely of ions with melting points near room temperature. Owing to their purely ionic composition, ILs exhibit unique physicochemical properties, including negligible vapor pressure, wide electrochemical windows, high thermal stability, and intrinsic ionic conductivity. These IL properties arise from the delicate balance of noncovalent interactions including Coulombic (ion–ion), van der Waals (steric effects) and hydrogen-bonding interactions.^16–18^

The development of ILs has driven extensive research into their potential applications as electrolytes in rechargeable batteries, supercapacitors, fuel cells, and as media for electrochemical synthesis, catalysis, and separations.^19–22^ This interest is partly motivated by their high chemical tunability, which enables the systematic variation of the cation and anion chemistry. These chemical changes in the IL give access to a vast number of possible combinations, allowing us to tailor key physicochemical properties such as viscosity, dielectric response, hydrophobicity, and ion-coordination behavior. However, despite their versatility, the practical implementation of ILs is often hindered by poor mass transport.^23^ Further, characterization of the IL structure is complicated by nanoscale segregation into ionic and nonionic domains, leading to highly heterogeneous and probe-dependent dynamical behavior that challenges the interpretation of experimental observables.^24^

Recent research has demonstrated that, in the limit of HCEs, conventional electrolytes and ILs converge.^25–27^ An example of this convergence is solvate ionic liquids (SILs),^28^ which form via the chelation of cations. This last process produces stable supercations at a solvent-to-salt molar ratio of one,^29^ which, despite their binary composition, have a behavior similar to ILs.^30^ The vast number of possibilities among conventional electrolytes, HCEs, and ILs highlights the need for a unified molecular framework capable of describing their underlying structures, solvation environments, and transport mechanisms. In this context, advanced spectroscopic techniques capable of resolving ultrafast molecular dynamics have emerged as powerful tools for probing time-dependent fluctuations of local ionic environments with high temporal and structural sensitivity. Among these methods, 2DIR spectroscopy is uniquely suited for capturing these molecular features, as it provides direct access to spectral diffusion, intermolecular energy transfer, and chemical exchange in complex environments (more details about these processes can be found in Section 3).

2DIR spectroscopy is rarely used as a stand-alone technique and is typically complemented by other time-resolved methods, including pump–probe spectroscopy and nuclear magnetic resonance (NMR). As a result, interpretations of electrolyte structure and dynamics often rely on the collective insights from these complementary approaches. Nevertheless, this review primarily focuses on 2DIR spectroscopic studies due to their unique ability to simultaneously probe molecular structure and dynamical processes with high temporal resolution and molecular specificity. By resolving these processes, 2DIR spectroscopy provides a powerful means for relating microscopic structural heterogeneity, including ion pairing, hydrogen-bonding interactions, and collective solvent fluctuations, to the molecular mechanisms that ultimately influence macroscopic behavior.

Spectroscopy measurements offer valuable insights into the origins of electrolyte properties, but establishing direct quantitative relationships between local spectroscopic observables and bulk transport metrics remains a significant challenge. Moreover, the local structures and dynamics that give rise to spectroscopic observables do not necessarily exhibit simple or direct correlations with macroscopic properties such as ionic conductivity, diffusivity, transference number, and viscosity, nor with bulk descriptors like dielectric constant, polarity, or ionic strength. This complexity arises because transport emerges from the collective interplay of numerous molecular processes spanning a broad range of temporal and spatial scales, whereas spectroscopic observables are often sensitive to specific local environments and are influenced by factors only indirectly related to macroscopic behavior. Furthermore, probe-dependent mechanisms, intermolecular vibrational coupling, orientational dynamics, reorientation-induced spectral diffusion, vibrational energy transfer, and photothermal contributions can all modulate the observed response and complicate the interpretation of the molecular aspects derived from 2DIR spectroscopy in terms of bulk transport properties.

Despite the challenges, a molecular framework for rationalizing electrolytes and ionic liquids has emerged from complementary studies performed across diverse chemical compositions, concentrations, temperatures, solvent environments, and ionic species. When analyzed collectively, these investigations reveal recurring structural motifs, dynamical time scales, and molecular mechanisms that relate individual systems. These emerging patterns provide the foundations for a molecular framework that links local solvation structure and dynamical heterogeneity to the collective processes that ultimately govern macroscopic transport, offering valuable molecular principles for the rational design and optimization of next-generation electrolyte materials.

This review begins by establishing the structural and spectroscopic foundations required to interpret these complex systems and by discussing how vibrational probes selectively access different aspects of solvation and transport. Subsequent sections examine how ion pairing, aggregation, and solvent reorganization govern the behavior of aqueous and organic electrolytes, before extending these concepts to ionic liquids, where structural heterogeneity, strong ion-ion interactions, and factors such as hydrogen-bonding and confinement give rise to distinct regimes of behavior. Finally, a section highlighting current challenges and emerging directions is presented.

## 2 Molecular Structure of Electrolytes and Ionic Liquids

Conventional electrolytes are typically composed of salts dissolved in a molecular solvent. At the microscopic level, these systems consist of discrete cations and anions whose interactions are strongly influenced by the surrounding solvent molecules (Figure 1a,b).^1^ Solvation plays a critical role here, as ions are stabilized through ion-dipole and/or hydrogen-bonding interactions, and dielectric screening provided by the solvent.^31,32^ The degree of ion dissociation in an electrolyte depends on the physicochemical characteristics of the solvent, the temperature, and the salt concentration. Hence, dilute electrolytes are often approximated as collections of largely independent solvated ions.^33^

**Figure 1:**
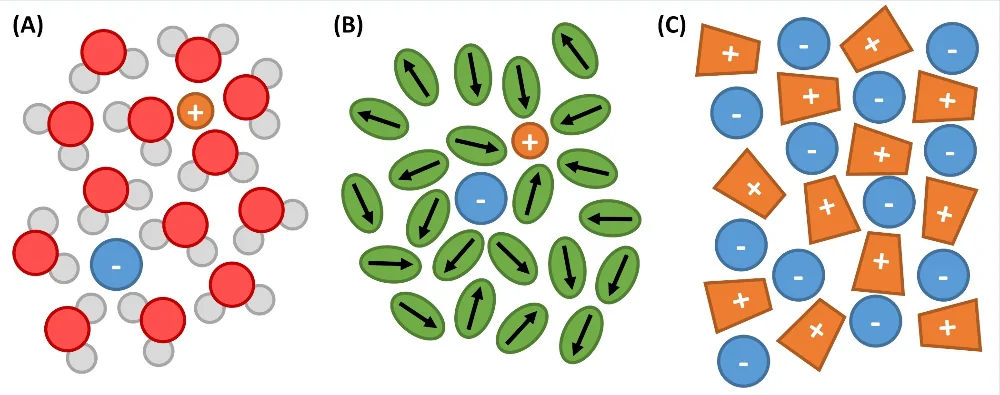
Cartoon representation of liquid electrolytes. (A) Aqueous electrolyte. (B) Organic electrolyte. (C) Ionic liquid. Each figure showcases the molecular structure of a cation and anion in water with the latter represented in red and gray circles (left) and in a molecular solvent represented by green ovals with arrows as dipoles (middle), and of an ionic liquid (right).

In dilute electrolytes, the solvation shell around ions is well-defined when the solvent has a strong affinity for the ionic components,^1,34^ such as in the case of water^2^ and organic carbonates.^35^ Under these conditions, the solvent molecules effectively coordinate the ions, forming stable, solvent-rich primary solvation shells that isolate the charged particles both spatially and electrostatically. Consequently, in diluted systems, Coulombic interactions are largely screened, and ions behave as independent species with only weak, long-range correlations.^32^ This screening also suppresses the formation of contact ion pairs (CIPs), and favors fully solvated, solvent-separated ion pairs (SSIPs).^34^

Water and organic solvents differ fundamentally in how they solvate ions (Figure 1). In water, solvation is governed by a combination of dipole reorientation and the restructuring of an extended hydrogen-bond network, enabling efficient stabilization of both cations and anions.^36^ Aqueous cations are coordinated through the oxygen lone pairs, with the hydrogen atoms oriented toward neighboring water molecules, such that water acts as a Lewis base while largely maintaining its hydrogen-bond connectivity. In contrast, anions are stabilized through directional hydrogen-bonds with O–H groups, which perturbs the local hydrogen-bonding network and leads to more restricted solvent dynamics. These distinct interactions have direct implications for vibrational spectroscopy. Because anion solvation involves O–H groups oriented toward the ion, changes in the O–H stretch vibrational modes provide a sensitive reporter of anion–solvent interactions and solvation structure.^37^ In contrast, water molecules coordinating cations lack a distinct spectroscopic signature, making cation solvation environments more challenging to isolate and characterize experimentally in aqueous solutions.^38,39^

This situation is effectively reversed in organic solvents. In the absence of hydrogen-bond donor functionality, solvation is dominated by cation-solvent interactions, where electron-rich groups such as carbonyls or ethers coordinate the cation, while anions remain comparatively poorly solvated.^35,40^ Consequently, vibrational probes in these systems capture the structure and dynamics of cation solvation shells, whereas solvent-anion interactions are significantly harder to resolve due to their weaker and less defined nature.^41^

The contrast in solvation between aqueous and organic electrolytes manifests in their transport properties. Aqueous electrolytes typically exhibit higher ionic conductivities than their organic counterparts, primarily because a larger fraction of ions remains dissociated in water,^42,43^ whereas ion pairing is more prevalent in organic solvents.^34^ In low concentration electrolytes, molecular solvents dominate the local ionic environment (Figure 2). As salt concentration increases, or when the solvent has a weaker affinity for ions, ion pairing and aggregation become increasingly important.^34^ Under these conditions, the distinction between “free” ions and ionic aggregates becomes less well-defined, leading to a continuously evolving speciation landscape. Consequently, describing the organic electrolyte structure across concentrations is inherently complex, as incomplete dielectric screening gives rise to stronger ion–ion correlations and the emergence of collective, network-like organization within the solution.

**Figure 2:**
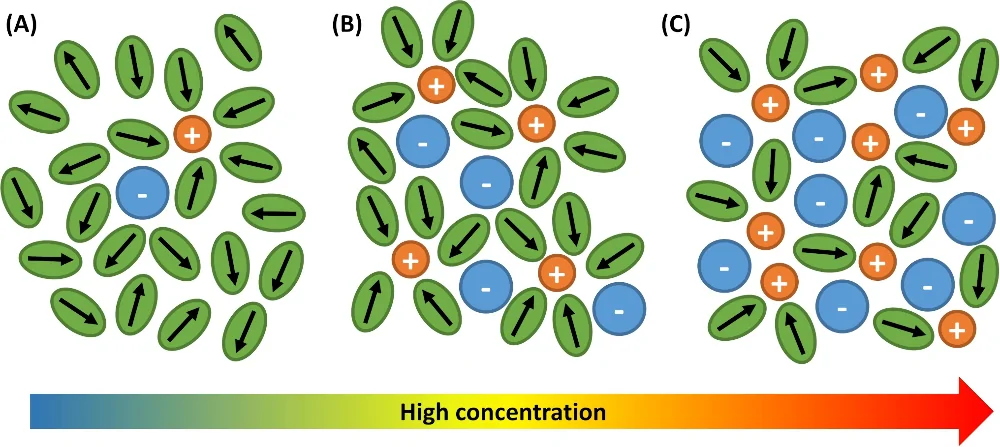
Cartoon representation of an electrolyte as a function of concentration. Each figure corresponds to a dilute (A), a concentrated (B), and a high concentration (C) electrolyte. Green ovals and arrows showcase the solvent and its dipole, respectively.

In the limit of maximum concentration, approached in high-concentration electrolytes (HCEs),^44^ where the solvent is no longer the dominant component, or in systems composed exclusively of ions without a molecular solvent, such as ionic liquids (ILs), the concept of discrete solvation shells breaks down entirely. For example, in ILs, the liquid state arises from the combination of bulky, asymmetric, and charge-delocalized organic cations with weakly coordinating anions. This pairing reduces lattice energy and suppresses crystallization, resulting in materials with melting points near ambient temperature.^45,46^ At the molecular level, the absence of solvent-mediated screening leads to strong Coulombic interactions that are counterbalanced by steric constraints.^47,48^ As a result, ILs exhibit pronounced short- and intermediate-range order, characterized by distorted charge alternation (Figure 1c) and, in many cases, nanosegregation into polar and nonpolar domains.^24,49^ Within this highly correlated environment, ion transport is no longer governed by the motion of independent species but instead proceeds through collective rearrangements within the interconnected ionic network.^50^

The difference between ion–ion and ion–solvent interactions also gives rise to distinct macroscopic behavior in salt solutions. Electrolytes with lower salt concentrations typically exhibit higher ion diffusivity and lower viscosity, but also higher solvent volatility and narrower electrochemical stability windows.^35,51^ In contrast, HCEs and ILs display higher viscosity, negligible vapor pressure, and wide electrochemical windows.^45,52,53^

Historically, conventional electrolytes have been represented at the molecular level by “static” solvation shells, in which ions do not interchange their solvating partners. However, studies using 2DIR spectroscopic methods have demonstrated that these structures are highly fluxional and continuously reorganize, making and breaking on picosecond timescales. These ultrafast processes have been observed to directly influence macroscopic properties, such as ionic conductivity and viscosity, but they remain inaccessible to techniques that rely on static structural models or ensemble-averaged measurements. 2DIR spectroscopy is uniquely capable of probing these processes. Furthermore, as electrolyte compositions expand to concentration regimes where many-body interactions and mesoscale organization dominate, describing molecular structure and dynamics on their intrinsic timescales becomes essential due to the lack of molecular theories that can explain them. Consequently, 2DIR spectroscopy is critical for establishing molecular frameworks across the full spectrum of electrolyte systems, from dilute solutions to ILs.

## 3 Principles of 2DIR Spectroscopy

The principles of 2DIR spectroscopy have been thoroughly described in the literature.^54–63^ Therefore, only a brief overview of the 2DIR method is presented here.

Conventional linear IR spectroscopy provides an ensemble-averaged view of molecular structure and local environments that, in general, cannot be used to determine whether vibrational transitions are coupled or to directly probe the timescales over which molecular environments evolve. 2DIR spectroscopy addresses both limitations by extending the experiment from a single frequency axis to two using a sequence of three temporally separated ultrafast infrared pulses. The 2D infrared spectrum and its time evolution provide structural and dynamical information about molecular systems with femtosecond-to-picosecond time resolution. This makes 2DIR spectroscopy a vibrational analog of 2D NMR, but with a temporal resolution many orders of magnitude faster. Since the first experimental realization in 1998 by Hochstrasser’s group,^64^ 2DIR spectroscopy has been applied to a wide range of condensed-phase systems ranging from proteins in solution to complex liquids, electrolytes, ionic liquids, and many more.

The fundamental principle of 2DIR spectroscopy is the use of a sequence of three ultrafast mid-infrared laser pulses that interact with the sample via its transition dipole moment, generating a third-order polarization field, usually called photon echo (Figure 3). The delays between these interactions define the key experimental time variables: the coherence time (τ), corresponding to the interval between the first and second pulses, the waiting time (T), corresponding to the interval between the second and third pulses, and the detection time (t), corresponding to the time interval between the third pulse and the fourth pulse used to measure the emitted third-order signal. In this interaction picture, the first pulse promotes the system from the ground state to a coherent superposition of vibrational states and the second pulse converts the coherence state into a population state. While in the population state, the system evolves during the waiting period and experiences changes in its local molecular environment. After a given time in the population state, the third pulse converts the population back into a coherent state, leading to the emission of a third-order nonlinear signal field (i.e., photon echo). The Fourier transform of the signal with respect to τ time yields the excitation-frequency axis (ω_τ_), while Fourier transformation with respect to t time produces the detection-frequency axis (ω_t_) (Figure 3). Hence, the resulting two-dimensional infrared (2DIR) spectrum correlates the excitation and detection frequencies, while the variation of the waiting time enables its evolution, and consequently, the direct observation of molecular structural fluctuations, intermolecular interactions, and dynamical processes.

**Figure 3:**
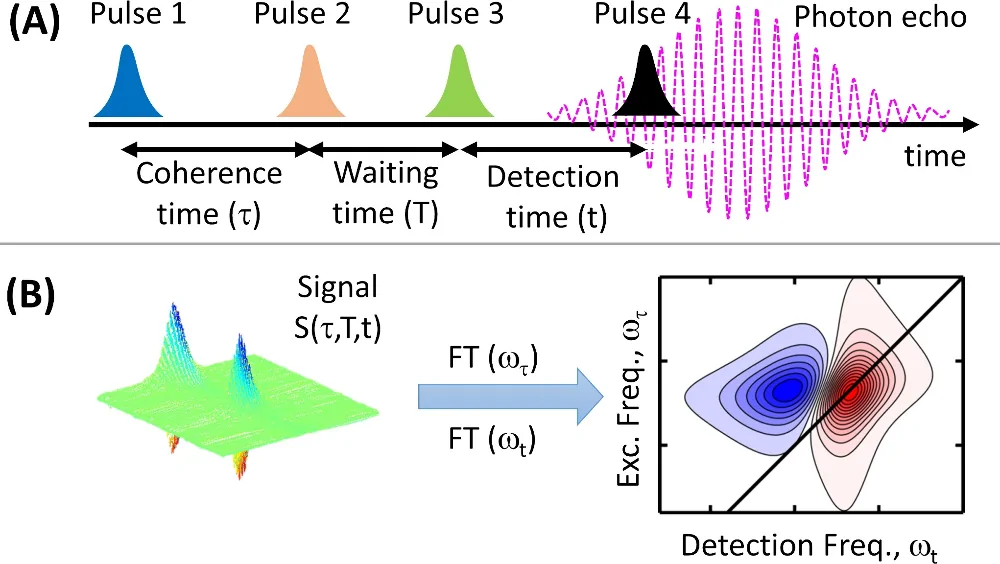
2DIR spectroscopy. (A) Pulse sequence in 2DIR spectroscopy and the relevant time delays between the pulses denoted as coherent time (τ), waiting time (T), and detection time (t). (B) Signal treatment to obtain the 2DIR spectrum.

The most prominent features of a 2DIR spectrum are the diagonal peaks. Diagonal peaks have direct correspondence to FTIR bands, in which a vibrational probe can observe multiple chemically distinct environments with distinct frequencies, such as those arising from a molecule residing within an ion solvation shell or remaining in the bulk phase. These chemically distinct environments, or equivalent IR bands, appear as a distinct diagonal feature in the 2DIR spectrum. However, because the 2DIR spectrum is created by three pulse interactions, each diagonal feature contains two peaks: one on the diagonal and one slightly off it. The former originates from transitions between the ground state and first excited state, whereas the latter arises from transitions between the first and second excited states, and it is shifted from the diagonal due to the anharmonicity of the vibrational potential (Figure 4).

**Figure 4:**
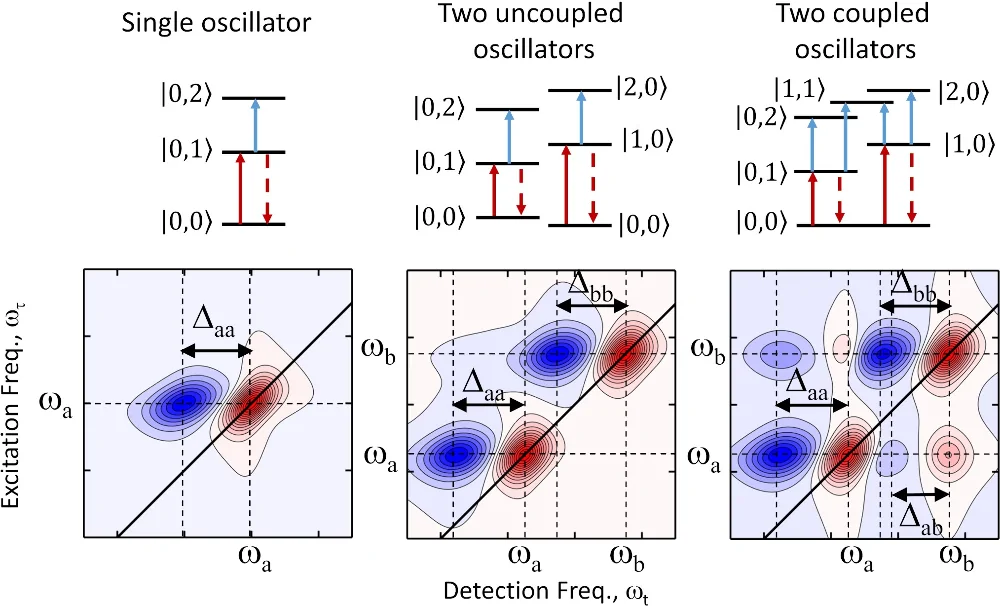
Signals in the 2DIR spectra. 2DIR spectra at zero waiting time and vibrational manifold for single oscillator and two oscillators without and with coupled transitions, where ω_i_ presents the position of the peaks, and Δ_ii_ represent the anharmonicities. In the 2DIR spectra, red is positive and blue is negative.

Beyond reporting different chemical environments, the evolution of 2DIR peak shapes with waiting time provides insights into the rate at which local molecular environments change. In the context of electrolytes and ionic liquids, these dynamics include processes such as ion solvation-shell rearrangements, hydrogen-bond fluctuations, and other intermolecular interactions that modulate vibrational frequencies. Consequently, the waiting-time dependence of 2DIR peaks offers a direct experimental window into the structural dynamics of complex liquids.

The physical basis of this behavior lies in the interplay between homogeneous and inhomogeneous broadening. Homogeneous broadening in the IR line shape results from processes that affect all probe molecules in the system similarly, such as vibrational lifetime and rapid rearrangements of the molecular environment (i.e., pure dephasing). Inhomogeneous broadening, in contrast, arises from a distribution of slightly dissimilar chemical environments sampled by the vibrational probe, which gives rise to a distribution of vibrational frequencies. The relative contributions of these broadening mechanisms can be quantified from the shape of 2DIR peaks. At zero waiting time, excitation and detection frequencies are maximally correlated, resulting in peaks elongated along the diagonal due to inhomogeneous broadening (i.e., the distribution of chemical environments), but narrow along the antidiagonal due to homogeneous broadening. As the waiting time increases, changes in the solvation environment of the probe alter the initially excited vibrational frequencies, leading to a progressive loss of diagonal elongation, reflecting the loss of correlation between excitation and detection. This process, commonly referred to as spectral diffusion, reflects frequency fluctuations associated with structural rearrangements of the surrounding environment. Simultaneously, the overall signal intensity decreases because of vibrational population relaxation; however, this process affects peak amplitude rather than peak shape.

The time dependence of these fluctuations is described by the frequency-frequency correlation function (FFCF, ⟨*δω*(0)*δω*(*t*)⟩), which provides a quantitative framework for relating measured spectral evolution to the underlying molecular dynamics. Several approaches have been applied to extract the FFCF from 2DIR spectra. Commonly used metrics include the center line slope (CLS),^65–68^ nodal line slope (NLS),^69^ dynamic mode decomposition,^70^ inhomogeneity index,^71,72^ eccentricity,^73^ ellipticity,^74^ pseudo-Zernike similarity (PZS),^75^ dynamic line width,^76,77^ among others. However, the relationship between experimentally measured line shape evolution and the FFCF is generally nontrivial, and these metrics rely on approximate relationships between observable line shape parameters and the underlying frequency fluctuations.^66,68,69^ Among these approaches, CLS and NLS are the methods most frequently used in the studies discussed throughout this review. These metrics monitor the waiting-time-dependent change in the diagonal tilt of 2DIR peaks, which reflects the progressive loss of frequency correlation associated with spectral diffusion.

Systems that exhibit more than one vibrational transition within the investigated IR window may also have off-diagonal features, which are commonly referred to as cross peaks (Figure 5). One possible mechanism that gives rise to cross peaks occurs when two vibrational modes are coupled. Two types of vibrational coupling exist: anharmonic coupling for modes in the same molecule, or dipole–dipole coupling for modes that are far apart or in different molecules. 2DIR cross peaks arising from vibrational coupling are often interpreted using the exciton formalism, where the coupling constant depends on the distance and orientation between the involved transition dipoles. Thus, cross peaks can provide information about coupling, energy transfer pathways, and the arrangement and proximity of molecular species.

As discussed throughout this review, these off-diagonal signatures have been extensively used to characterize the geometry of ion-solvation structures, contact ion pairs, and higher-order ionic aggregates in electrolytes and ionic liquids, complementing other structural methods on different timescales. Additionally, cross peaks from coupled vibrational transitions appear at zero waiting time and evolve according to specific pathways (Figure 5a). For example, cross peak dynamics, tracking energy flow between coupled vibrational modes, provides a direct experimental signature to characterize pathways of intra- and intermolecular energy redistribution. When combined with isotopic labeling or site-specific vibrational probes, 2DIR cross peak dynamics provide interaction maps that are difficult to access by other spectroscopic techniques. However, extracting structural parameters from these off-diagonal features often requires precise knowledge of the vibrational Hamiltonians.

**Figure 5:**
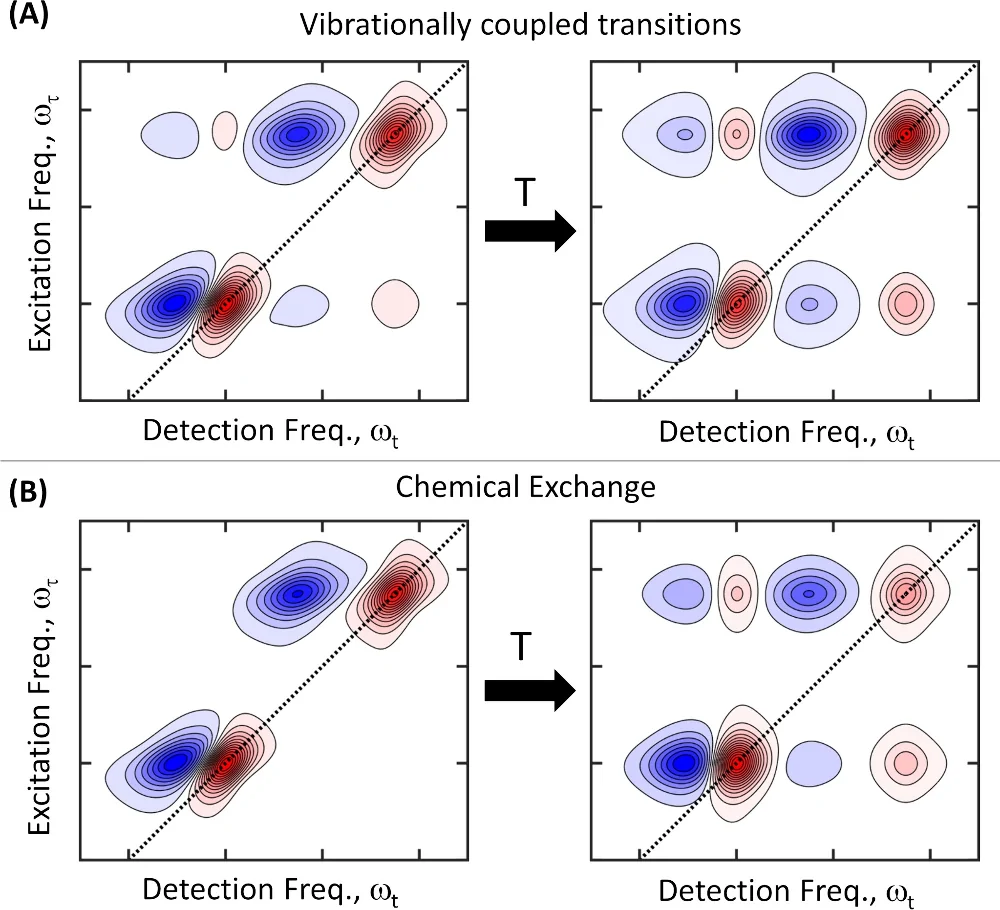
2DIR spectra with cross peaks. Temporal evolution of the 2DIR spectra for coupled transitions (top) and vibration modes corresponding to species undergoing chemical exchange (bottom).

2DIR spectral signatures of coherences also appear in systems having coupled vibrational transitions. Coherences manifest as oscillatory features in the signal over time, and in 2DIR spectra, they produce characteristic oscillatory modulations in peak shapes, tilts, or cross peaks. Hence, coherent 2DIR signatures provide insights into anharmonic coupling. The presence of this quantum effect is crucial for demonstrating and/or characterizing strongly coupled vibrational transitions, which are typically seen in many solvents used in electrolytes, such as water and organic carbonates.

Another possible mechanism that gives rise to cross peaks in the 2DIR spectrum is chemical exchange (Figure 5b). Cross peaks arising from such mechanism reflect the interconversion of molecules between distinct structural or chemical environments on a timescale comparable to the waiting time of the experiment, which is typically on the picosecond timescale. When the vibrational mode of a molecule experiences environments with different frequencies, the exchange of the molecular environment during the waiting period leads to cross peaks connecting the initial and final environments, or equivalent, frequencies of the molecule. Since chemical exchange dynamics are slower than the duration of femtosecond IR pulses, molecules largely retain their molecular environment during excitation (zero waiting time). Consequently, the initially absent chemical exchange cross peaks grow with increasing waiting time due to the speciation exchange occuring in the picosecond timescale. As a result, 2DIR chemical exchange cross peaks serve as a spectral signature of dynamical processes, including conformational switching, hydrogen-bond making and breaking, and ionic speciation exchange in complex molecular systems.

Additional structural information can be obtained through polarization-dependent 2DIR spectroscopic measurements. Because vibrational coupling and transition dipole orientations influence polarization-dependent signals, 2DIR anisotropy measurements provide insights into molecular orientation, rotational dynamics, and local structural organization. Together, spectral diffusion, cross-peak dynamics, and anisotropy measurements allow 2DIR spectroscopy to simultaneously probe molecular geometry, relative orientation of vibrational transition dipole moments, and ultrafast orientational dynamics in liquids, biomolecules, and complex condensed-phase systems.

Despite all the information encoded in a 2DIR spectrum, the technique alone does not provide a direct and unambiguous assignment of spectroscopic signatures to specific molecular configurations or interactions. Experimental observables, including vibrational frequencies, lineshapes, cross peaks, and their waiting-time evolution, arise from entangled structural and dynamical processes and therefore are not easily interpreted from the 2DIR spectra alone. Consequently, the analysis of 2DIR data often relies on the complementary insights provided by molecular dynamics (MD) simulations and vibrational frequency maps,^78^ or other methods.^79^ MD simulations provide the atomistic time evolution of the system, containing the structural fluctuations and environmental heterogeneities that give rise to the vibrational response. Frequency maps and other methods translate these instantaneous molecular configurations into vibrational frequencies, from which vibrational dynamics can be directly assessed or used to compute the linear and 2D vibrational spectra. Comparison between experiments and simulations therefore enables spectroscopic observables to be linked to specific molecular structures, interactions, and dynamical processes, providing a molecular-level interpretation of the measured vibrational response.

Finally, it is also important to note that the ability to extract physically meaningful insights from these signals is intrinsically tied to the choice of vibrational reporter, since the measured dynamics reflect the specific interactions and environments sampled by the probe. In complex media such as electrolytes and ILs, where heterogeneity, strong electrostatic interactions, and nanostructured domains coexist, this consideration becomes particularly critical. Accordingly, the following section discusses the range of vibrational probes used in these systems, emphasizing how their chemical identity, localization, and spectroscopic properties govern their sensitivity to distinct structural motifs and dynamical processes.

## 4 Vibrational Probes in Electrolytes and Ionic Liquids

Vibrational modes respond to the molecular environment through shifts in their central frequencies, and to fluctuations of local environments and their dynamics through changes in the IR line shape. Consequently, applying 2DIR spectroscopy to these systems requires probes that not only possess suitable spectroscopic properties but also selectively report on specific local environments within the electrolyte or IL matrix.

### 4.1 Guidelines for the Selection of Vibrational Probes

The choice of vibrational probe determines both the accessible spectral window and the extent to which the resulting spectra can be unambiguously interpreted. The most effective IR reporters for studying electrolytes and ILs generally have the following characteristics in the selected vibrational mode: (i) a strong transition dipole, (ii) an associated frequency appearing in an isolated region of the IR spectrum, and (iii) sensitivity to the molecular environment.^80–82^ Additional considerations may also include choosing the size of the molecular probe to enable high-level computational modeling of solute–solvent interactions, or selecting a vibrational mode with an appropriate vibrational lifetime for the timescale of the process being investigated. For example, the solvation dynamics obtained from spectral diffusion, which is caused by the picosecond fluctuations of the molecular environment, only requires vibrational modes with fairly short lifetimes.^54^ In contrast, studying chemical exchange requires probes with sufficiently long vibrational lifetimes to capture the process within the experimental time window. The nitrile group is a representative example, as its nitrile stretch meets the three primary criteria, exhibits a sufficiently long vibrational lifetime and often presents resolved IR bands corresponding to the distinct subensembles (ion pair-free, coordinated-free, aggregated, etc.).^83^

Most vibrational probes deviate from the ideal case, and there are inevitable trade-offs between their capabilities and limitations in different contexts. For example, probes with very large transition dipoles (e.g., organic carbonates) require specialized cells with extremely short path lengths,^84–86^ and sometimes produce coupled vibrational transitions if their modes are close in frequency to other vibrational modes of the system.^87^ These effects complicate both spectral interpretation and quantitative modeling.

In many electrolytes and ILs, suitable intrinsic vibrational modes from either the ions and/or the solvent may simply be absent, requiring the use of external probes. While often effective, the use of external reporters can perturb the electrolyte structure, and create probe–probe interactions at higher concentrations. Moreover, probes impose practical constraints, such as chemical compatibility, water sensitivity, and electrochemical stability, restricting the applicability of IR reporters in many realistic battery electrolyte formulations. Overcoming these challenges is not straightforward, but the trade-offs are system dependent. Thus, a wide range of molecular probes and experimental strategies have been used to gain valuable insights and advance our molecular understanding of electrolytes. Using external probes in ILs is particularly challenging because they are single component systems, meaning that molecules can modulate their properties more strongly compared to traditional electrolytes. In the following sections, a summary of the most commonly used probes in the field is presented.

### 4.2 Anion-Based Probes

Anions with strong, sensitive, narrow, and spectrally isolated vibrational modes in the IR region are excellent built-in reporters of the local structure in electrolytes. Their vibrational frequencies directly reveal the strength of the cation–anion interaction potential, such as the case with Li^+^, and consequently, their speciation as free ions, contact ion pairs (CIPs), or aggregates (AGGs).^26,88,89^ Because these are usually intrinsic vibrational signatures of the solution components, anion vibrational modes often provide a nonperturbative means of probing electrolyte and IL organization.

Various anionic species that exhibit the aforementioned desirable characteristics of the nitrile stretch have been used in 2DIR studies. These anions include thiocyanate (SCN^–^),^90–92^ cyanide (CN^–^),^93^ cyanate (OCN^–^),^94^ selenocyanate (SeCN^–^),^95,96^ dicyanamide ion (N­(CN)_2_^–^),^97,98^ tricyanomethanide ion (C­(CN)_3_^–^),^99,100^ and metal complexes, such as [Fe­(CN)_6_]^3–^, [Fe­(CN)_6_]^4–^, [Co­(CN)_6_]^4–^ or [Ru­(CN)_6_]^4–^.^101–104^ From this list, the thiocyanate anion is a notable case because it interacts differently through its nitrogen and sulfur ends,^105^ and presents distinct responses to hydrogen-bonding and cation coordination. This dual sensitivity makes SCN^–^ a powerful probe for elucidating the mechanisms by which different cations interact with the anion in aqueous and organic electrolytes and in ILs.

The azide ion (N_3_^–^) is another widely used anion probe.^106–108^ Like nitriles, the azide ion exhibits a strong transition dipole and high environmental sensitivity, and is spectrally isolated. However, its symmetric structure makes spectral interpretation and modeling more difficult than with the nitrile group.^109^ Moreover, its practical application is limited by safety considerations because some azide salts are difficult to handle.^110^

Despite their advantages, none of the aforementioned anions can be incorporated into practical electrolyte formulations because they undergo electrochemical decomposition at relatively low potentials, limiting their stability in real battery environments. Nevertheless, some anions commonly found in electrolytes feature vibrational modes that are well-suited for 2DIR experiments. These anions include nitrates, imidazole-based lithium salts, and sulfonimides.^89,111,112^ Beyond battery applications, other anions, such as sulfates,^113,114^ phosphates,^115,116^ borohydrides,^117,118^ and carboxylates,^119,120^ serve as prototypical systems for studying ion pairing and hydrogen-bond dynamics in aqueous electrolytes relevant in the biological field. A similar case is observed for ILs, where anion-based ILs, such as those containing tricyanomethanide, have shown particularly low viscosity,^121^ making them ideal for studying their molecular structure through the nitrile groups of the anion.^100,122^

Not all anions with sensitive vibrational modes can be effectively probed using 2DIR spectroscopy. Practical constraints such as the spectral range of femtosecond IR laser sources, detector sensitivity, the transparency windows of IR optical materials, and the overlap with vibrational modes of the solvent can limit which vibrational modes are experimentally accessible. Consequently, many anions with valuable vibrational signatures have been studied using other infrared techniques, such as FTIR, ATR, or Raman spectroscopy. Examples include perchlorates,^123^ nitrites,^124^ and borates,^125^ which are informative but fall outside the most accessible spectral regions for ultrafast IR methods.^54^

### 4.3 Cation-Based Probes

In 2DIR spectroscopy, cation-based vibrational reporters provide complementary views by probing the environment surrounding the positively charged species in electrolytes and ILs. This selective probing provides access to solvation dynamics that are often difficult to extract from anion based probes alone. For instance, when an IR active anion resides near a cation, its vibrational dynamics reflect changes in both the solvation shell and the reorganizations involving nearby cations. Thus, an IR active cationic probe provides a suitable way to disentangle ion–ion and ion–solvent interactions. Moreover, the interaction between a cation and the surrounding solvent molecules, particularly water, differs fundamentally from that of an anion because the orientation of the solvent dipoles in the cation solvation shell is reversed (Figure 1). As a result, the solvation dynamics observed from a cationic probe are expected to differ from those obtained using anionic reporters offering a valuable insight into processes that play a central role in the electrochemical performance of energy storage systems.

Metal cations and many molecular cations lack suitable intrinsic vibrational modes, and so the application of 2DIR spectroscopy to cationic species is quite limited. Examples of molecular cations include guanidinium^126,127^ and imidazolium derivatives including a C–D group^128^ or with covalently bonded SeCN group.^129^ Metal complex cations with vibrationally active ligands have also been used, including species such as [Re­(CO)_3_(CH_3_CN)_3_]^+^,^130^ and [Mn­(CO)_5_]^+^.^131^ Despite possessing IR-active modes, and its relevant application in battery electrolytes, ammonium (NH_4_^+^) has not been used in 2DIR studies due to the limited spectral resolution and vibrational overlap. Especially in the case of water, where its study has been limited to FTIR and Raman spectroscopy.^132–134^

As discussed above, probing electrolytes and ILs through their ionic components offers both significant advantages and inherent challenges. An alternative strategy involves directly interrogating the vibrational modes of the solvent in the case of electrolytes, or added neutral solutes and small organic solvents to ILs. This approach is broadly applicable and constitutes the most commonly used method in 2DIR spectroscopy for studying electrolyte dynamics, although its use in IL systems remains more limited.

### 4.4 Solvents and Solutes as IR Probes

In electrolytes containing vibrationally inactive salts, the molecular structure and dynamics can be directly probed using the solvent as an intrinsic IR reporter, even under operando conditions. Water and most organic solvents contain the polar functional groups necessary for ion coordination, and these groups naturally exhibit strong, well-defined vibrational modes that are ideal for 2DIR spectroscopy. The use of solvent vibrational modes represents a conceptual shift from using ionic reporters, as it enables us to obtain a collective view of the system dynamics, rather than a local view shaped by ion-specific interactions. The solvent vibrational modes also enable direct access to processes, such as solvent reorientation, chemical exchange, and hydrogen-bond rearrangement, by probing the time evolution of diagonal line shapes and the appearance of cross peaks in the 2DIR spectra.

An example of an intrinsic vibrational mode of the solvent is the carbonyl stretch of organic carbonates.^85,135^ This vibration lies in a relatively free IR spectral region and is highly sensitive to coordination with metal ions, making it an effective reporter for tracking solvation shell structure in electrolytes. In particular, free and cation bound carbonyl populations are often spectroscopically resolved allowing individual analysis of their spectral diffusion dynamics, while cross peaks arising from the chemical exchange between these two populations provide direct insights into solvent exchange processes and solvation dynamics within the electrolyte.^85,136^

Solvent molecules constitute the dominant component of most electrolytes, but their vibrational modes, such as carbonyl stretches can exhibit strong anharmonic coupling and large transition dipole moments, resulting in excessively strong absorption and detector saturation. To mitigate these effects, some studies have used alternative functional groups with weaker transition dipoles. For example, nitrile stretching modes, whose transition dipole strengths are more than 20 times smaller than those in carbonyls, have been used to study lithium electrolytes.^137^ While this approach effectively mitigates issues related to strong absorption and vibrational coupling, the results are not always easily transferable across solvents. In the latter case, the two solvents may interact similarly with the cation but not with the anion, leading to differences in ion speciation.^138^ Another alternative is to dilute the electrolyte with a structurally similar but IR inactive solvent. This lowers the absorbance of the desired vibrational mode of the solvent while enabling the study of its interactions with ions.^137^ However, this approach does not guarantee that the mixed electrolyte will preserve the same ionic speciation as the original system, since small changes in enthalpy can significantly alter ion association equilibria. In addition, a dilution inherently modifies solvent–solvent interactions by reducing the local concentration of the original solvent.

Similarly to specific vibrational modes of organic solvents, the hydroxyl stretch of water in aqueous electrolytes provides a highly sensitive, molecular level probe of molecular structure when investigated using time-resolved vibrational spectroscopy.^139–142^ This sensitivity is essential because the arrangement of water molecules, ions, and the surrounding hydrogen-bond network is linked to macroscopic properties relevant to electrochemical systems, including ion transport, and conductivity.^142,143^ Beyond bulk structure, local and intermediate structures, such as hydration shells, hydrogen-bond connectivity, and ion pairing configurations, play a decisive role in governing ion mobility and reactivity in aqueous environments.^144^ Accordingly, elucidating how ions distort or reorganize the water hydrogen-bond network is essential for predicting the behavior of aqueous electrolytes across different temperatures and concentrations, and in the presence of confinement.^39,145–147^

The capacity of water to form strong, directional hydrogen-bonds produces an extended and dynamically interconnected network that shapes its vibrational response. Intermolecular coupling between neighboring O–H oscillators promotes rapid delocalization of vibrational excitations and efficient energy flow through the solvent network, which in turn accelerates vibrational relaxation and dephasing of the O–H stretching mode.^148^ These ultrafast collective processes shorten excited-state lifetimes and obscure the links between spectroscopic observables and specific local solvation environments, making it difficult to disentangle structural dynamics from energy redistribution in neat H_2_O.^149^ To overcome these limitations, studies have typically focused on the O–D stretch in an isotopic mixture of dilute HOD in H_2_O to isolate the individual oscillators.^143,150–152^ This approach suppresses intermolecular energy transfer, extends vibrational lifetimes, and restores the local nature of the vibrational mode required for quantitative interpretation of 2DIR spectra in terms of hydrogen-bond and solvation dynamics.

Due to the lack of intrinsic suitable reporters, ILs benefit from the use of neutral solutes as vibrational probes. The most notable example is carbon dioxide, as ILs have been proposed and deployed for use in its industrial-scale extraction. Carbon dioxide is an interesting case because it provides insights into the overall structure and dynamics of the IL, as well as its solvation shell, which helps explain its solubility.^153–160^ In addition, some reports investigated ILs diluted with molecular solvents, such as water or alcohols, using 2DIR spectroscopy,^161^ while others have examined water or benzonitrile solutions using complementary vibrational spectroscopic techniques, including FTIR, Raman, and ATR spectroscopy.^162–164^

In principle, built-in IR reporters avoid the perturbations introduced by external molecular probes. However, relying solely on the solvent can be challenging when it lacks favorable spectroscopic properties. For these reasons, some studies have adopted solvent-mimic vibrational probes that closely resemble the host solvent while offering improved spectroscopic performance. Examples include organic thiocyanates (CH_3_SCN)^91,165^ and phenyl selenocyanate (PhSeCN)^96^ in conventional organic electrolytes. In these cases, the probes contain a nitrile stretch allowing the observation of different bonding environments (e.g., solvent–solvent versus ion–solvent) and the tracking of relatively slow dynamics, such as chemical exchange, which intrinsic solvent vibrational modes often cannot access. Notably, both CH_3_SCN and PhSeCN have been shown to reproduce the characteristic dynamics of the original solvents in electrolytes, yielding spectral diffusion time constants comparable to those obtained using the O–D stretch of HOD in water or the carbonyl stretch in propylene carbonate.^141,166^ However, the use of these molecular probes still suffers from limitations similar to those previously described for ionic reporters. An alternative strategy that avoids these challenges while retaining the advantages of intrinsic probing is the use of selective isotope labeling.

### 4.5 Isotopic Labeling

Isotopic labeling is a powerful method for reducing the complexity of certain vibrational probes while maintaining the interaction potential essentially unchanged. The method consists of substituting certain atoms that participate in the vibrational mode of interest with heavier isotopes, such as D, ^13^C, ^15^N, and ^18^O. Replacing lighter atoms with their heavier analogues shifts the vibrational mode to lower frequencies, isolating its associated vibrational band from the original or other overlapping modes. Examples of this can be found in studies of amides,^167^ thiocyanate,^168^ and water^169,170^ to name a few. Hence, the isotopic labeling strategy, combined with 2DIR spectroscopy, provides a way to isolate single vibrational transitions, simplifying the interpretation of the 2DIR spectra and IR line shape analysis.^170^

Isotopic substitution is also an effective method for reducing or controlling vibrational coupling, allowing one to probe specific local modes without strong excitonic delocalization. While isotopic labeling enables the isolation of vibrational modes from anharmonically coupled transitions (both excitons and Fermi resonances), it also opens the possibility of a multireporter strategy, in which the isotopologues act as secondary reporters with distinct frequencies for otherwise identical functional groups. This isotope labeling strategy has been used in combination with 2DIR spectroscopy to probe site-specific solvation and intermolecular interactions, from which geometrical constraints, such as distance,^171^ as well as solvent/ion exchange processes have been obtained.^168^

The main drawbacks of the isotope labeling strategy are the high cost of isotopically labeled materials and the need for custom synthesis when labeling specific molecules. Also, in many cases, a single isotopic substitution is insufficient to isolate the desired vibrational mode, requiring double isotopic labeling.^172^ Nevertheless, mechanistic 2DIR studies that require the disentanglement of overlapping vibrational bands often rely on isotopic labeling because it provides a direct method of separating, assigning, and interpreting the spectral IR components.^173^

### 4.6 Compiled Overview of Probes and Studied Systems

The broad range of vibrational probes discussed above highlights that the access to specific structural motifs and dynamical processes is inherently defined by the nature and placement of the IR reporter. In this context, interpreting results across studies requires careful consideration of the probe-dependent perspective provided by each experiment. To aid the reader in navigating this diversity, Tables 1, 2, 3, 4, 5, and 6 are presented in this section to compile all the systems investigated by 2DIR spectroscopy, along with their corresponding salt concentrations, the probes used to study them, and the main 2DIR observables discussed in the work, including solvation dynamics (SD), chemical exchange (CX), vibrational coupling (VC), vibrational energy transfer (VET), molecular photothermal effect (MPTE) and 2DIR anisotropy (2DA). To facilitate comparison across studies, concentrations originally reported as mole fractions or molar ratios were converted to molality (*m*) whenever possible. Concentrations reported in molarity (M) were retained in their original form when the information required for conversion, such as solution density, was not available. A more comprehensive version of the tables is provided in the Supporting Information, including the specific vibrational modes used for the reporters and their location within the system (anion, cation, solvent/solute, or isotopically labeled component). By organizing this information into a set of tables, a concise summary of the investigated systems is provided as a preview of the material discussed in the following sections.

**Table 1:** Compilation of Lithium Salt-Containing Electrolytes Studied with 2DIR Spectroscopy^a^

Salt	Solvent	Salt concentration	Probe	Main 2DIR observable	Reference
LiCl	Water	9–14 *m*	HOD	SD	^174^
	Water	1–5 M	acetone	SD	^175^
	Water	9–14 *m*	CH_3_SCN	SD	^139,141,176^
	Water (electrode interface)	0.1 M	4-mercaptobenzonitrile	CX	^177^
LiBr	Water	14 *m*	CH_3_SCN	SD	^176^
	DMF	0.5 M	SCN^–^	CX	^178^
	CH_3_CN/CH_3_SCN	0.5–1 M	CD_3_CN, CH_3_SCN, SCN^–^	VC, VET, MPTE	^179^
LiSCN	Water	4 M	SCN^–^	VET	^168^
	Water	5 *m*	SCN^–^/S^13^C^15^N^–^	VET	^180^
	EC-PC	0.1 M	SCN^–^	SD	^181^
	EC-PC (GPE)	0.03 M	SCN^–^	SD	^182^
	DMF	0.1–0.7 M	SCN^–^	SD, CX	^178,181^
	CH_3_CN	1.3 *m*	CH_3_CN	SD	^137^
	CH_3_CN/CH_3_SCN	0.05–0.7 M	CD_3_CN; CH_3_SCN; SCN^–^	VC, VET, MPTE	^179^
	CH_3_NO_2_	1.2 M	SCN^–^	SD, CX	^183^
	Benzonitrile	1.2 M	SCN^–^	SD, CX	^183,184^
	G1, G2, G4	2–9 *m*	SCN^–^	CX	^26^
	EtAc	1.2 M	SCN^–^	SD, CX	^183^
	CHCl_3_	0.1 *m*	SCN^–^	SD	^185^
	DMC	1.2 M	SCN^–^	SD, CX	^183^
LiTFSI	Water	0.2–5 M	LiTFSI	2DA	^89^
	Water	0.2–22 M	HOD	SD, VC	^142,143,186^
	Water	1–18 *m*	SCN^–^	SD	^140^
	PC	1–3 M	PhSeCN	SD, CX	^166^
	PC–PPC	1:6 salt/solvent ratio	PhSeCN	SD, CX	^187^
	PC-DMC	1.3 M	PhSeCN	CX	^188^
	CH_3_CN	0.2–16 *m*	multiple CH_3_CN isotopes	SD, VET	^137,189,190^
	BC, MP, DMC	3–8 *m*	carbonate	SD, VET	^136^
	DMI, TMU, TEU	0.5–0.8 *m*	urea	SD, VET	^191^
LiBETI	CH_3_CN	12 *m*	CD_3_CN	SD	^192^
LiFTFSI	CH_3_CN	12 *m*	CD_3_CN	SD	^192^
LiFSI	CH_3_CN	12 *m*	CD_3_CN	SD	^192^
	DMC-TTE	1–3 M	carbonate	SD, VET	^193^
LiNO_3_	Water	14 *m*	CH_3_SCN	SD	^176^
LiClO_4_	CH_3_CN-DMSO	6 M	CH_3_CN	SD, VC	^194^
	EC-PC (GPE)	1:9 salt/solvent ratio	SCN^–^	SD	^182^
	CH_3_CN	2 M	CH_3_SCN	CX	^195^
	DMC-THF	1 M	carbonate	SD, VET	^196^
LiNO_2_	Water	14 *m*	CH_3_SCN	SD	^176^
LiBF_4_	CH_3_CN	1 *m*	CH_3_CN	CX	^197^
LiPF_6_	PC	1–3 M	carbonate	VET, MPTE	^198^
	PC-DMC	1 M	carbonate	VET	^135^
	BC	0.9–2 *m*	carbonate	SD, VET, MPTE	^85,198^
	EMC	1–3 M	carbonate	SD, VET, MPTE	^198–200^
	DMC	1–3 *m*	carbonate	SD, VET, MPTE	^85,198–201^
	DEC	1–3 M	carbonate	SD, VET, MPTE	^135,198–200^
LiTDI	BC	0.5 *m*	TDI^–^	SD, VC	^112^
	DMC	0.5 *m*	TDI^–^	SD, VC	^112^

a A complete list of the abbreviations used in this table can be found in the last section of the manuscript.

**Table 2:** Compilation of Sodium Salt-Containing Electrolytes Studied with 2DIR Spectroscopy^a^

Salt	Solvent	Salt concentration	Probe	Main 2DIR observable	Reference
NaCl	Water	6 M	N_3_^–^, CH_3_COO^–^	SD	^202^
	Water	1–4 M	acetone	SD	^175^
NaBr	Water	1–6 M	HOD	SD	^146,149–151,203^
NaI	Water	3–4 M	SCN^–^	CX	^204^
NaCN	Water	2.5 M	CN^–^	SD	^205^
NaSCN	Water	0.05–4 M	SCN^–^	SD, CX, VET	^90,92,140,168,204,206–208^
	Water	5 *m*	SCN^–^/S^13^C^15^N^–^	VET	^180^
	Water (membrane)	0.0001 M	SCN^–^	SD	^209,210^
	Water (normal-phase micelle)	0.125 M	SCN^–^	SD	^208^
	Water (reverse micelle)	0.125 M	SCN^–^	SD	^208^
	THF	0.070 M	SCN^–^	SD, CX	^211^
	G2,G3,G4	0.5 M	SCN^–^	SD	^212^
	CHCl_3_	0.1 *m*	SCN^–^	SD	^185^
NaN_3_	Water	0.05–0.25 M	N_3_^–^	SD, VC	^106,107,213^
	Water (membrane)	0.0001 M	N_3_^–^	SD	^209^
NaNO_3_	Water	1–8 M	NO_3_^–^	2DA, VC	^111,214^
NaClO_4_	Water	6 M	HOD	CX, SD, 2DA	^215–217^
NaBF_4_	Water	5.5 M	HOD	SD, CX	^149,152^
	Water	6 *m*	HOD, BH_4_^–^	SD	^118^
Na_2_SO_4_	Water	1.5 *m*	HOD	SD	^203^
	Water	0.2 M	SO_4_^2–^	SD	^114^
NaPO_2_(Me)_2_	Water	0.2 M	[PO_2_(Me)_2_]^2–^	SD, VC	^116,218,219^
NaPF_6_	Water	5 M	HOD	CX	^220^
	PC-DMC (1:1)	1 M	carbonate	SD, VC	^221^
	HFE-DMC (4:6)	1 M	carbonate	SD, VC	^221^
	DMC	1 M	carbonate	SD, VC	^221^
Na_2_[Fe(CN)_5_NO]	Water	0.072 M	[Fe(CN_5_NO)]^2–^	SD	^222^
NaTFA	Water	0.125 M	CH_3_COO^–^	SD	^119^

a A complete list of the abbreviations used in this table can be found in the last section of the manuscript.

**Table 3:** Compilation of Potassium Salt-Containing Electrolytes Studied with 2DIR Spectroscopy^a^

Salt	Solvent	Salt concentration	Probe	Main 2DIR observable	Reference
KF	Water	5 *m*	SCN^–^/S^13^C^15^N^–^	VET	^223^
KI	Water	5 *m*	SCN^–^/S^13^C^15^N^–^	VET	^223^
KCl	Water (electrode interface)	0.1 M	4-mercaptobenzonitrile	CX	^177^
KCN	Water	0.9 M	D_2_O	SD	^224^
KSCN	Water	0.2–10 M	SCN^–^	VET, SD	^168,225^
	Water	2–10 M	SCN^–^/S^13^C^15^N^–^	VET	^168,180,223,226^
	Water	0.05–6 *m*	SCN^–^; H_2_O	VET	^227^
	MeOH	0.2 M	SCN^–^	SD	^228^
	THF	Saturated solution	SCN^–^	SD, CX	^211^
	CHCl_3_	0.1 *m*	SCN^–^	SD	^185^
KOCN	MeOH	0.05 M	SCN^–^	SD	^228^
KSeCN	Water	0.1–0.5 *M*	SeCN^–^	SD	^229,230^
	DMF	0.4 M	SeCN^–^	CX	^231^
K_2_CO_3_	Water	5 *m*	SCN^–^/S^13^C^15^N^–^	VET	^223^
KC(CN)_3_	Water	0.1 M	C(CN)_3_^–^	SD, VET	^99^
KC(CN)_3_	Water (reverse micelle)	0.001 M	C(CN)_3_^–^	SD	^147^
KH_2_PO_4_	Water	1 M	H_2_PO_4_^–^	SD, VC	^232,233^
K_2_HPO_4_	Water	5 *m*	SCN^–^/S^13^C^15^N^–^	VET	^223^
K_3_[Co(CN)_6_]	Water	0.08 *M*	[Co(CN)_6_]^3–^	SD, CX	^103^
	Glycerol	0.08 *M*	[Co(CN)_6_]^3–^	SD, CX	^103^
	Ethylene glycol	0.08 *M*	[Co(CN)_6_]^3–^	SD, CX	^103^
K_3_[Fe(CN)_6_]	Water	0.29 M	[Fe(CN)_6_]^3–^	SD	^102^
K_4_[Fe(CN)_6_]	Water	0.03–0.04 M	[Fe(CN)_6_]^4–^	SD	^102,225,234^
K_4_[Ru(CN)_6_]	Water	0.07 M	[Ru(CN)_6_]^4–^	SD	^104^

a A complete list of the abbreviations used in this table can be found in the last section of the manuscript.

**Table 4:** Compilation of Monovalent Cation-Containing Electrolytes Studied with 2DIR Spectroscopy^a^

Salt	Solvent	Salt concentration	Probe	Main 2DIR observable	Reference
CsCl	Water	10 M	C_2_O_4_^2–^	SD, CX	^235^
AgSCN	DMTF	0.5 M	SCN^–^	CX	^105^
CuSCN	DMTF	0.5 M	SCN^–^	CX	^105^
CsSCN	Water	4 M	SCN^–^	VET	^168^
	Water	5 *m*	SCN^–^/S^13^C^15^N^–^	VET	^180^
	CHCl_3_	0.1 *m*	SCN^–^	SD	^185^
	DMTF	0.5 M	SCN^–^	CX	^105^
NH_4_Br	Water	5.6 *m*	NH_4_^+^; H_2_O	VET	^227^
NH_4_SCN	Water	2–11 *m*	SCN^–^; NH_4_^+^; H_2_O	VET	^227^
	PC	0.05 M	SCN^–^	SD	^236^
	THF	0.07 M	SCN^–^	SD, CX	^211^
N(Bu)_4_SCN	NMF	0.2 M	SCN^–^	SD	^237^
	FA	0.2 M	SCN^–^	SD	^237^
	DMSO	0.2 M	SCN^–^	SD	^237^
	CH_3_CN	0.2 M	SCN^–^	SD	^237^
	DMF	0.2 M	SCN^–^	SD	^237^
N(Bu)_4_N_3_	NMF	0.07 M	N_3_^–^	SD	^237^
	DMSO	0.07 M	N_3_^–^	SD	^237^
	MeOH	0.07 M	N_3_^–^	SD	^237^
Rb_2_SO_4_	Water	1–2 *m*	HOD	SD	^203^
Cs_2_C_2_O_4_	Water	0.1 M	C_2_O_4_^2–^	SD, CX	^235^
GdmCl	Water	0.1 M	DGdm^+^	VET	^127^
GdmCl	d-Glycerol/Water (59%)	0.2 M	DGdm^+^	VET	^127^
GdmAc	DMSO	0.4 M	CH_3_COO^–^; DGdm^+^	VC, 2DA	^126^

a A complete list of the abbreviations used in this table can be found in the last section of the manuscript.

**Table 5:** Compilation of Divalent Cation-Containing Electrolytes Studied with 2DIR Spectroscopy^a^

Salt	Solvent	Salt concentration	Probe	Main 2DIR observable	Reference
MgCl_2_	Water	3 M	SCN^–^	CX	^206^
	Water	2 M	[PO_2_(Me)_2_]^2–^	SD, VC	^116,218,219^
	Water	0.5–4 *m*	SeCN^–^	SD	^238^
	Water (silica)	2.5–4.2 *m*	SeCN^–^	SD	^145^
	Water (electrode interface)	0.1 M	4-mercaptobenzonitrile	CX	^177^
MgBr_2_	Water	3 M	SCN^–^	CX	^206^
	Water	2 *m*	HOD	SD	^203^
MgI_2_	Water	1.5–3 M	SCN^–^	CX	^204,206^
CaCl_2_	Water	3M	SCN^–^	CX	^206^
	Water	0.5–2 M	[PO_2_(Me)_2_]^2–^	SD, VC	^116,219^
CaBr_2_	Water	3 M	SCN^–^	CX	^206^
CaI_2_	Water	3 M	SCN^–^	CX	^204,206^
ZnCl_2_	Water	1–4 M	acetone	SD	^175^
Mg(TFSI)_2_	CH_3_CN	0.2–11 *m*	multiple CH_3_CN isotopes	VET	^189,190^
Ca(TFSI)_2_	CH_3_CN	0.2–8 *m*	multiple CH_3_CN isotopes	VET	^189,190^
Zn(TFSI)_2_	CH_3_CN	0.3–4 *m*	multiple CH_3_CN isotopes	VET	^189,190^
Ba(TFSI)_2_	CH_3_CN	0.3–6 *m*	multiple CH_3_CN isotopes	VET	^189,190^
Mg(ClO_4_)_2_	Water	3.4 M	SCN^–^	SD, CX	^90^
Ca(ClO_4_)_2_	Water	3M	SCN^–^	CX	^206^
MgSO_4_	Water	0.2–1 M	SO_4_^2–^	SD	^114^
	Water	0.5–2 M	HOD	SD	^203^

a A complete list of the abbreviations used in this table can be found in the last section of the manuscript.

**Table 6:** Compilation of Ionic Liquids Studied with 2DIR Spectroscopy^a^

IL	Probe	Main 2DIR observable	Reference
[BMIm][BF_4_]	[BMIm][DCA]	SD, VC	^98^
	[BMIm][TCM]	SD	^100^
	NaN_3,_ TBAN_3,_ 2-azido ethanol, 3′-azido3′-deoxythymidine, 3-azido propylamine	SD	^239^
	HOD	SD	^240,241^
[BMIm][DCA]	CO_2_	SD, VC	^154^
[BMIm][PF_6_]	NaN_3,_ TBAN_3,_ 2-azido ethanol, 3′-azido3′-deoxythymidine, 3-azido propylamine	SD	^239^
	CO_2_	SD, VC	^154^
	D_2_O, HOD	SD, VET	^242^
[BMIm][SCN]	[BMIm][SCN] (colloidal suspension)	SD	^243^
	[BMIm][SCN]	VC	^244^
	CO_2_	SD, VC	^154^
[BMIm][TFA]	[BMIm][TCM]	SD	^100^
	CO_2_	SD, VC	^154^
[BMIm][TfO]	CO_2_	SD, VC	^154^
[BMIm][TFSI]	2-SeCN-BmimTFSI	SD	^129^
	[BMIm][SCN]	SD	^245,246^
	[BMIm][SCN] (in thin films)	SD	^246^
	[BMIm][SeCN]	SD	^129,247,248^
	[BMIm][SeCN] (in thin films)	SD	^247,248^
	SCN^-^	SD	^249^
	KSeCN	SD	^250^
	[BMIm][TFSI]	VC	^244^
	CO_2_	SD, VC	^154,156^
[BMMIm][TFSI]	[BMIm][SCN]	SD	^245,251^
	KSCN	SD	^251^
[DMIm][BF_4_]	HOD	SD	^240^
[DMIm][TFSI]	[DMIm][SeCN] (in bulk and in thin films)	SD	^248^
	KSeCN	SD	^250^
	CO_2_	SD	^156^
[EMIm][BF_4_]	[EMIm][TCM]	SD	^100,122^
	NaN_3,_ TBAN_3,_ 2-azido ethanol, 3′-azido3′-deoxythymidine, 3-azido propylamine	SD	^239^
	HOD	SD	^240^
[EMIm][CH_3_COO]	[EMIm][CH_3_COO]	VC	^244^
[EMIm][FAP]	[D-EMIm][FAP]	SD, VC, CX	^128,252^
	W(CO)_6_	VC	^253^
[EMIm][TFA]	[EMIm][TFA]	VC	^254^
[EMIm][TFSI]	[EMIm][SeCN]	SD	^255,256^
	[EMIm][SeCN] (membrane)	SD	^256^
	SCN-	SD	^249^
	KSeCN	SD	^250,255^
	CO_2_	SD	^153,156–158,160^
	CO_2_ (membrane)	SD	^157^
	CO_2_ (polymer matrix)	SD, VET	^159^
	CD_3_OD,CD_3_CD_2_OD	SD	^161,257^
	HOD	SD	^161,186,257^
[EMImOH][BF_4_]	[EMIm][TCM]	SD	^100^
[EtNH_3_][NO_3_]	NH_4_SCN	SD, 2DA	^258^
[HMIm][Cl]	N(Bu)_4_SCN	SD	^259^
[HMIm][FAP]	W(CO)_6_	SD	^253^
[HMIm][PF_6_]	CD_3_OD	SD	^257,260^
	W(CO)_6_	SD	^253^
[HMIm][TFSI]	[HMIm][SeCN] (in bulk and in thin films)	SD	^248^
	SCN^-^	SD	^249^
	KSeCN	SD	^250^
	CO_2_	SD	^156^
[MMPyrr][TFSI]	CO_2_	SD	^155^
[Mpyr][TFSI]	CO_2_	SD, VC	^155^

a A complete list of the abbreviations used in this table can be found in the last section of the manuscript.

## 5 Organic and Aqueous Electrolytes

### 5.1 Ion Pairing and Salt Aggregation

Ultrafast 2DIR spectroscopy provides a unique molecular-level view of ion pairing and ligand exchange in electrolyte solutions by simultaneously resolving structurally and spectrally distinct solvation states and their picosecond interconversion. Over the years, 2DIR studies have revealed how ion identity, solvation structure, and local interactions govern the formation and dissociation of contact ion pairs and larger aggregates in both aqueous and organic electrolytes. These insights have been complemented by other time-dependent methods, such as pump–probe spectroscopy and anisotropy.

#### 5.1.1 Ion Association and Exchange in Aqueous Electrolytes

Ion pairing in aqueous media is governed by the balance between hydration structure and anion identity, where both determine the propensity for contact ion pair (CIP) formation, as well as the pathways and timescales of dissociation. Over the past decades, 2DIR spectroscopy studies have established a molecular-level picture of ion pairing in water electrolytes. These studies have revealed that CIP formation and interconversion typically occur on tens of picoseconds timescales for alkali and alkaline earth metal ions, reflecting the dynamic competition between direct ion–ion interactions and solvent-mediated stabilization.^90,116,204,206,218,219^ Furthermore, investigations of symmetric ions like oxalate have shown that ion complexation can induce symmetry breaking, giving rise to multiple distinct ion pair geometries that also interconvert on picosecond timescales.^235^

Beyond CIP in single salt systems, methodological advances in 2DIR spectroscopy have enabled the study of competing vibrational energy transfer pathways, providing a quantitative framework for assessing relative binding affinities in multicomponent electrolytes.^227^ More recently, the study of electrolytes has also been extended to strongly acidic environments, where ion pairing has been shown to significantly modulate local proton structure and hydration dynamics.^214^ Collectively, these findings demonstrate that ion pairing in aqueous solutions is a highly dynamic and environment-dependent process that can be systematically studied using ultrafast infrared spectroscopies.

Foundational evidence for CIP formation was established by Park et al. in a 2DIR study in 2010. In this work, the authors assigned two distinct vibrational bands to the free SCN^–^ and its blue-shifted Mg-SCN^+^ contact pair (Figure 6a,b,c).^90^ The authors determined a CIP dissociation time of ∼ 52 ± 10 ps and a formation time of ∼ 140 ± 30 ps. The authors also proposed that CIP dissociation proceeds primarily through perchlorate-thiocyanate exchange within the first solvation shell of Mg^2+^, rather than via water exchange. Furthermore, the authors observed a slower characteristic time scale of the orientational dynamics relative to the ion pair dissociation, suggesting that CIPs dissociate before complete reorientation occurs.

**Figure 6:**
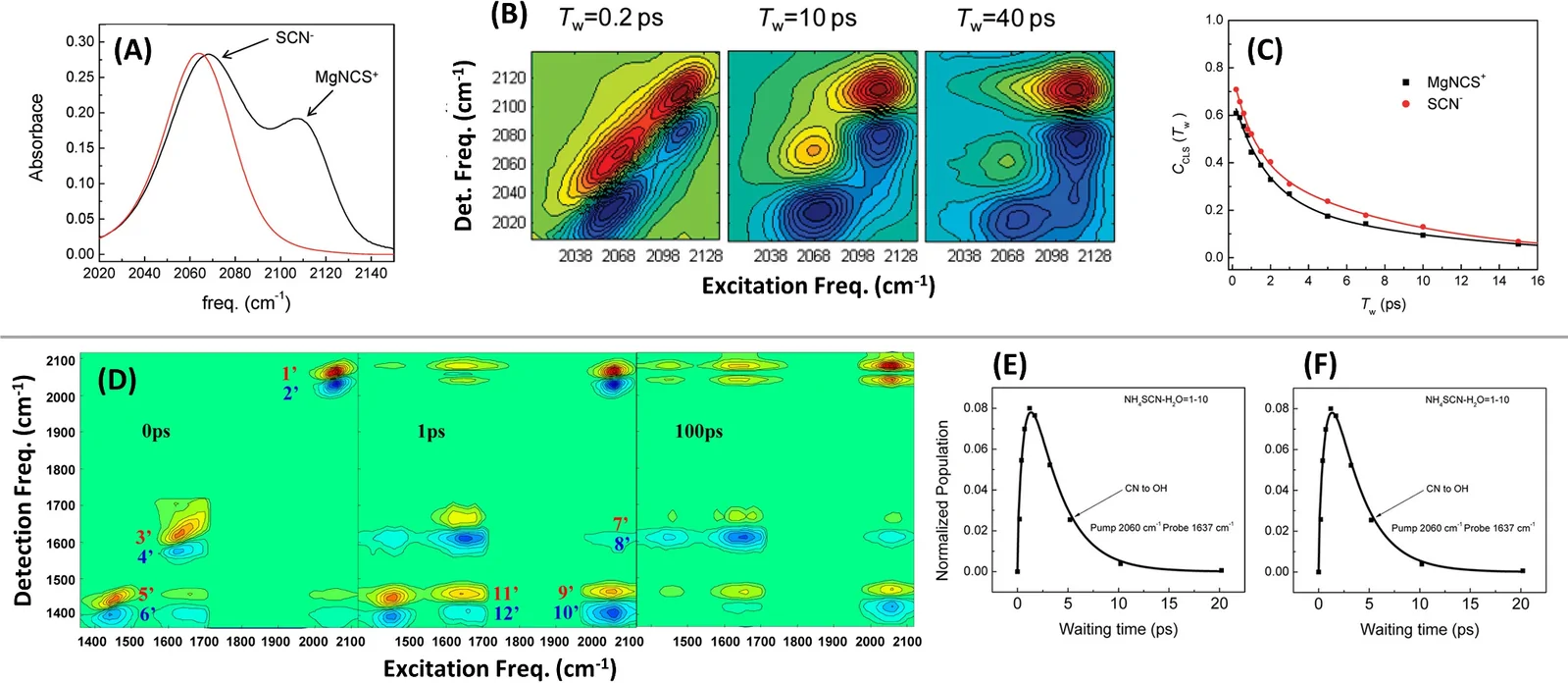
Ion pairing in aqueous electrolytes. Top panel: FTIR and 2DIR spectra of thiocyanate (SCN^–^) ion in D_2_O. (A) NaSCN (red curve), and Mg­(ClO_4_)_2_ and NaSCN (black cruve) dissolved in D_2_O. (B) 2DIR spectra obtained from Mg­(ClO_4_)_2_ and NaSCN dissolved in D_2_O at different T_W_s. (C) Spectral diffusion dynamics of free SCN^–^ ion and MgNCS^+^ contact ion pair in D_2_O, where data points are the experimental center line slopes and the solid line a biexponential fit. Adapted with permission from ref.^90^ Copyright 2010 American Chemical Society. Bottom panel: (D) Waiting time dependent 2DIR spectra of the KSCN/H_2_O (1/10 molar ratio) solution. Cross peak evolution of the CN to OH (E) and CN to NH (F) through vibrational energy transfer in the KSCN/H_2_O (1/10) solution. The dots are experimental data and the lines are their modeling. Adapted with permission from ref.^227^ Copyright 2013 American Chemical Society.

Despite their chemical similarity, divalent cations exhibit markedly different ion pair dissociation mechanisms when competing anions are present. Sun et al. showed that the Mg-SCN^+^ dissociation time is largely insensitive to the concentration of I^–^, consistent with a dissociative anion-exchange mechanism.^204^ In contrast, Ca-SCN^+^ CIP dissociation accelerates at higher I^–^ concentrations, indicating an associative exchange where incoming anions promote displacement. Strikingly, water exchange solvating Ca^2+^ is 5 orders of magnitude faster than that of Mg^2+^, while anion residence times for the two cations remain comparable. These results evidenced that cation-specific shell rigidity and local geometry play a key role in ion-pair dynamics, rather than being determined by the bulk water exchange rates.

The role of ion–solvent interactions in stabilizing distinct solvation shell configurations was further elucidated in a follow-up work by Sun et al.^206^ The authors studied the anion exchange within the first solvation shell of Mg^2+^ and Ca^2+^ for anions with different Lewis base softness (Cl^–^, Br^–^, I^–^, and ClO_4_^–^). By inferring CIPs from chemical-exchange cross peaks in 2DIR spectra, the authors observed that hard Cl^–^ anions did not show exchange, whereas other softer anions did. The preference of hard Lewis acids (Mg^2+^, Ca^2+^) for softer bases (Br^–^ and I^–^) challenged both hard–soft Lewis acid–base (HSAB) theory and Collins’ law of matching water affinities. These results also suggested that ion–water interactions and anion-induced perturbations of the hydration-shell are as critical as direct ion–ion interactions in defining the solution stability of ions.

Studies of phosphate model systems reinforced the role of cation identity in determining CIP structure. Using dimethyl phosphate ions as probes,^116,218,219^ Fingerhut et al. and Schauss et al. in two different studies observed blueshifts of the asymmetric PO_2_^–^ stretch upon Mg^2+^ addition, attributing the CIP induced shift to Coulombic interactions and exchange repulsion.^218^ The magnitude of the shift followed the trend: Mg^2+^ > Ca^2+^ > Na^+^, indicating sensitivity to cation charge density and local structure. Moreover, 2DIR spectra did not reveal interconversion among ion pairs within the probe lifetime (a few hundred femtoseconds).^116^ Calculations linked the changes in the vibrational manifold to structural differences among CIPs, with Mg^2+^ forming the most rigid and compact geometry, while Ca^2+^ and Na^+^ present a larger number of structural arrangements.^219^

Direct evidence for multiple CIP geometries and their dynamics was provided by Kuroda et al. in their study of aqueous solutions of cesium oxalate.^235^ The authors identified two possible geometries of CIPs (side-on and end-on) that interconvert on picosecond timescales, indicating a shallow potential energy surface between the two species. Notably, diagonal-peak dynamics revealed anomalously slow spectral diffusion for CIP modes, consistent with partial insulation of carboxylate groups from bulk water fluctuations upon ion pairing.

The Zheng group introduced the use of vibrational energy transfer to quantify competing relaxation pathways and ion binding affinities in electrolytes (Figure 6d,e,f).^227^ Li et al. showed that in solutions with a 1:10 salt to water molar ratio, ∼ 90% of the CN-stretch energy of KSCN flows to the water bending mode within few picoseconds. In contrast, in a similar NH_4_SCN solution, only 49% of the vibrational energy is transferred to water, while 44% flows to the NH bending mode of NH_4_^+^ on a comparable picosecond timescale, highlighting the predominance of NH_4_SCN CIPs over solvent-separated pairs (SSIP). The results evidenced a much larger affinity of NH_4_^+^ for SCN^–^ than that for water, demonstrating that CIP formation arises from specific ion–ion interactions, rather than from the statistics associated with high ion concentrations.

Finally, a study performed in acidic media revealed the formation of contracted ion pairs in concentrated alkali nitrate solutions. Lewis et al. observed cross peaks between NO_3_^–^ vibrations and the water bend of molecules in its solvation shell. The authors also showed the presence of H^+^-NO_3_ from a distinct anisotropic 2DIR cross peak between NO_3_^–^ and the hydrated protons at concentrations larger than 2 M.^214^ Finally, the authors observed that undissociated HNO_3_ persists alongside the contracted ion pairs even at 1–2 M, with both species showing similar concentration dependencies.

#### 5.1.2 Ion Association and Exchange in Organic Electrolytes

Similar to aqueous systems, in organic electrolytes, 2DIR spectroscopy has been widely used to quantify CIP lifetimes, to probe how ion–ion, solvent–ion, and solvent–solvent interactions govern ionic speciation, and to resolve the geometries of ion coordination.

Early work by Ji et al. on LiSCN in benzonitrile provided one of the first demonstrations of 2DIR measurements resolving ion pair dynamics in nonaqueous media. The authors demonstrated that the interconversion occurs on the order of tens of picoseconds (Figure 7a,b).^184^ Subsequent work by Lee et al. on LiSCN in DMF, however, showed that a more strongly coordinating solvent significantly slows the interconversion processes by stabilizing free ions relative to CIPs, causing the association and dissociation times to extend to hundreds of picoseconds.^178^ Furthermore, the authors observed that ionic association in polar organic solvents is governed by a balance between enthalpy and entropy, where endothermic, yet entropically favorable association typically occurs.^178^ The particularly long timescales in DMF were later extended by Park et al. using the SeCN^–^ probe (Figure 7c–g), where the long vibrational lifetime allowed the authors to observe the subnanosecond dissociation dynamics of the system.^261^ Together, these studies provided clear evidence that solvent polarity and coordination strength influence ion pair interconversion timescales.

**Figure 7:**
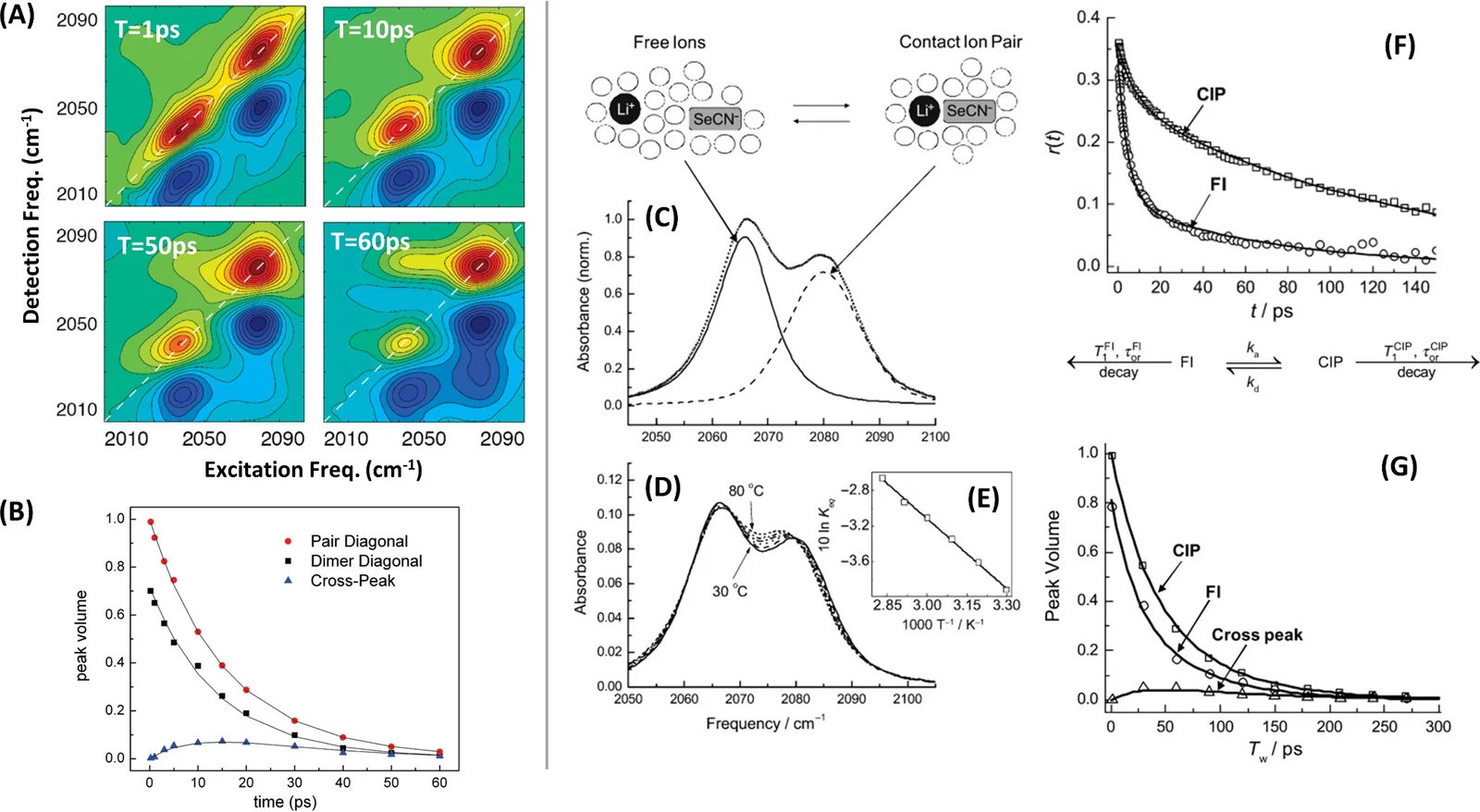
Ion pairing in organic solvents. Left panel: Ion pairing dynamics of LiSCN solution in benzonitrile. (A) Time evolution of 2DIR spectra of the LiSCN solution in benzonitrile. (B) Waiting time evolution of peak volumes for diagonal and the cross peaks extracted from the 2DIR spectra. Solid lines correspond to kinetic model. Adapted with permission from ref.^184^ Copyright 2010 American Chemical Society. Right panel: Ion pairing dynamics of SeCN^–^ and Li^+^ solution in dimethylformamide (C) FTIR spectrum of SeCN^–^ and LiBr in dimethylformamide, with its decomposition into free SeCN^–^ and Li^+^-bound. (D) Temperature-dependent FTIR spectra of the same system, with (E) the van’t Hoff plot as inset. (F) Orientational relaxation dynamics (anisotropy decays) of free ions and Li^+^-bound. (G) Peak volumes (data points) of the free ions and Li^+^-bound and of the cross peaks as a function of waiting time. The lines are fits to the two-species exchange kinetic model. Adapted with permission from ref.^261^ Copyright 2010 John Wiley and Sons.

Building on these findings, Ji et al. investigated the effect of different organic solvents on ion-pairing timescales, and the impact of the interplay between conformational and chemical interconversion dynamics in LiSCN.^183^ In solvents forming stable Li^+^-solvent complexes, like benzonitrile, dimethyl carbonate, and ethyl acetate, the ion-pair dissociation was found to occur on faster timescales than the reorganization of the solvent molecules coordinated to the Li^+^. This behavior contrasted with that observed in nitromethane solutions, a very weakly coordinating solvent, highlighting the importance of solvent Lewis basicity in governing ion assembly pathways. This study also addressed the spectral diffusion dynamics in the ion pair dimers, where the dissociation depends more on electrostatic reorganization than on local solvent rearrangements.^183^ Apart from the effects of dielectric constants and Lewis basicity, complementary work by Rushing et al. on LiTDI showed that the solvent structure can dictate the ionic speciation between CIPs and SSIPs. Although FTIR alone cannot fully resolve these species, the FFCFs clearly show a difference between them. While linear carbonates favor CIPs with a fast FFCF dephasing, cyclic carbonates stabilize SSIPs or free ions, which exhibited slower spectral diffusion timescales.^112^

The length of the solvent molecule and the size of the solvent–ion complexes are also important variables capable of determining speciation and dynamics. As shown in the study by Galle Kankanamge et al. on NaSCN in glymes of varying length, the conformational dynamics of CIPs strongly depend on the size and flexibility of the Na^+^-glyme cavity that hosts the anion.^212^ Spectral diffusion dynamics showed two distinct timescales, which increase with glyme length: a fast wobbling anion motion in the cavity, and a slower fluctuation of the cavity itself. The authors concluded that longer glymes impose stronger local and global reorientational constraints on the Na^+^-glyme complex, reflecting their greater structural complexity. Similarly, Zhou et al. studied metal-ether complexes in a crown ether to investigate how changing the alkali metal modulates binding selectivity.^185^ Using SCN^–^ as a vibrational reporter, the authors observed only small frequency shifts across the Li^+^ to Cs^+^ series, but large variations in spectral diffusion dynamics. Li^+^, Na^+^ and Cs^+^ complexes displayed a single exponential decay, consistent with moderate confinement, whereas K^+^ complexes exhibited a pronounced biexponential dynamics, indicative of stronger geometric restriction within the cavity.

The ability of 2DIR spectroscopy to resolve couplings between vibrational modes has also enabled direct interrogation of ion-pair structure beyond the exchange dynamics. In particular, 2DIR spectroscopy has provided signatures of geometric constraints, preferred binding configurations, and the dynamical consequences of different types of ion coordination. For example, Huerta-Viga et al. observed that distinct 2DIR cross peaks have orientation-specific vibrational coupling amplitudes arising from the coplanar geometry of the guanidinium-acetate ion pair in DMSO.^126^ Kwon et al. also extended the structural sensitivity of 2DIR spectroscopy to the ambidentate nature of thiocyanate, which can coordinate through either its nitrogen or sulfur terminus, showing how cation identity can control both the binding modes and the exchange kinetics of the CIPs.^105^ In particular, this study showed that Cu^+^ samples favored N-bound SCN- configurations and exhibited faster exchange, whereas Ag^+^ systems preferred S-binding with slower exchange dynamics. The authors linked the binding mode to the activation barriers governing exchange, emphasizing that ion-ligand coordination plays a central role in ion pair dynamics in organic media. Finally, Deshmukh et al. demonstrated that variations in spectral diffusion of LiSCN CIPs across multiple solvents can be explained by thiocyanate coordination, even when different binding modes exhibit minimal central frequency shifts, thereby allowing the distinction between the N-bound and S-bound species from the FFCF timescales.^181^

#### 5.1.3 Ionic Aggregation in Aqueous Electrolytes

The work on ionic aggregation in highly concentrated aqueous electrolytes has focused primarily on systems containing SCN^–^ and TFSI^–^. Here, 2DIR spectroscopy has been used to track vibrational energy transfer, manifested as the growth of cross peaks arising from ion–ion and ion–solvent interactions. Collectively, the 2DIR results demonstrated that both the structure and dynamics of ionic aggregates are highly sensitive to the ion identity and size.

Bian et al. provided the initial spectroscopic evidence of ionic clustering in aqueous solutions by monitoring cross peaks growth between isotopically labeled SCN^–^ and ^13^S^15^CN^–^ anions and their changes as a function of salt concentration (Figure 8a,c).^168,171^ The authors observed that in highly concentrated KSCN solutions (∼10 M), nearly all thiocyanate anions (∼95%) participate in ionic clusters, indicating a strong thermodynamic driving force toward aggregation. These ionic clusters exhibited extended solid-like organization reminiscent of crystalline KSCN. Upon dilution to 1 M, both the size and number of clusters decreased, but vibrational energy transfer accelerated, suggesting the persistence of smaller aggregates. Comparative measurements on 4 M solutions of LiSCN, NaSCN, KSCN, and CsSCN further revealed a pronounced cation-dependent effect, where larger and more polarizable cations, such as Cs^+^, form larger aggregates, whereas smaller cations yield fewer and smaller clusters.

**Figure 8:**
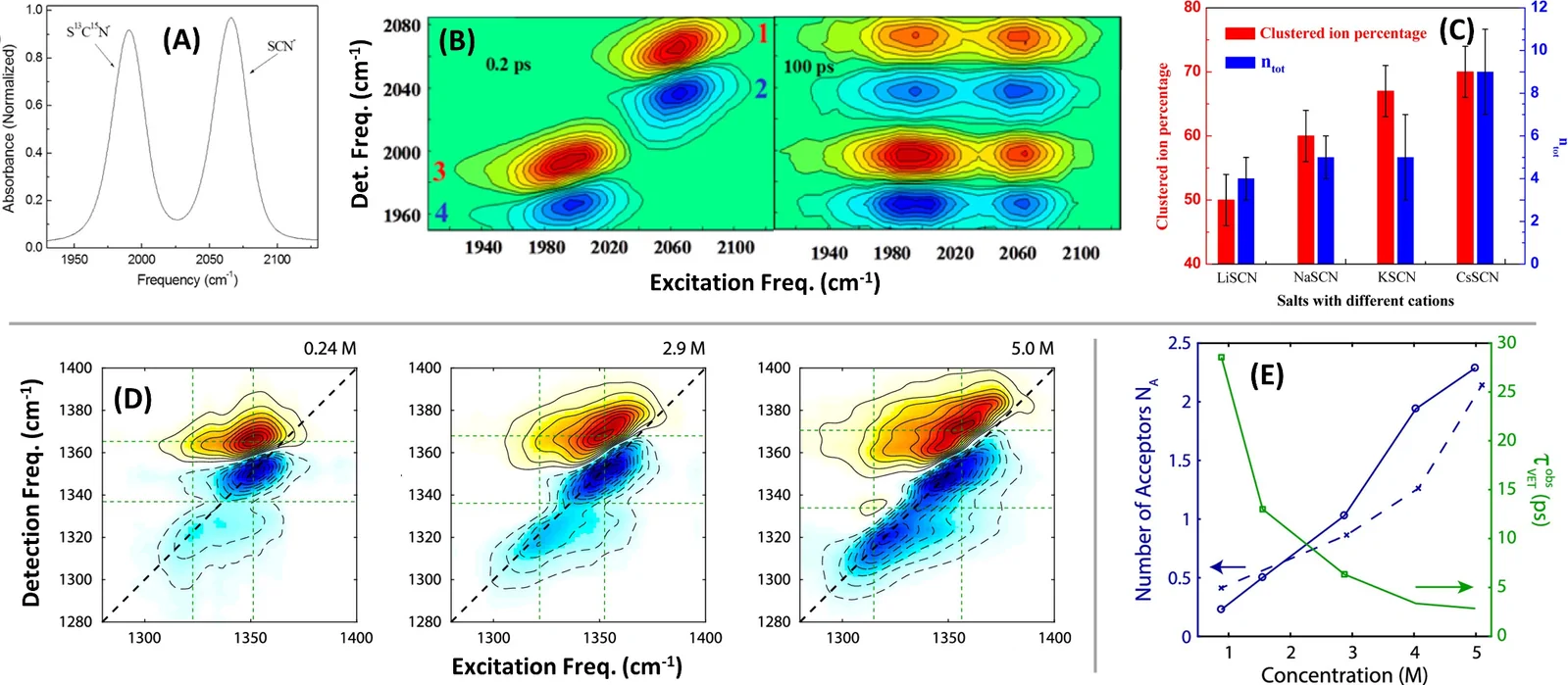
Ionic aggregation in aqueous solutions. Top panel: KSCN solution in D_2_O. (A) FTIR spectrum of the CN and ^13^C^15^N stretches of SCN^–^ and S^13^C^15^N^–^ for a 10 M KSCN/KS^13^C^15^N. (B) The waiting time evolution of 2DIR spectra for the same solution. (C) Cation dependence of the ion cluster percentage and the number of anions (*n*_tot_) in an energy transfer unit of 4 M aqueous solutions of LiSCN, NaSCN, KSCN, and CsSCN. Adapted with permission from ref.^171^ Copyright 2015 American Chemical Society. Bottom panel: high concentration LiTFSI in water. (D) Isotropic 2DIR spectra of the νSO_2a_ bands in solutions at 0.24, 2.9, and 5.0 M LiTFSI in water concentrations at *T* = 0.15 ps. (E) Number of acceptor TFSI^–^ ions (N_A_, blue left axis) determined from the experiments (solid line) and from the Molecular Dynamics (MD) simulations (dashed line) and the observed time scale of vibrational energy transfer (right axis, green squares) as a function concentration. Adapted with permission from ref.^89^ Copyright 2020 American Chemical Society.

Subsequent work by the same group demonstrated that aggregation is also strongly modulated by anion-specific hydration effects.^223^ Using the same SCN^–^/^13^S^15^CN^–^ isotopic mixture, the authors showed that adding large quantities of weakly hydrated anions, such as I^–^, slowed both vibrational energy transfer and rotational dynamics. This behavior was attributed to the association of ionic species that disrupted the proximity between thiocyanates. In contrast, strongly hydrated anions, such as F^–^, CO_3_^2–^, and HPO_4_^2–^, enhanced SCN^–^ clustering by withdrawing water from the thiocyanate ion local environment, thereby “pushing” these anions into closer proximity. These results highlighted the role of competitive hydration in controlling ionic aggregation.

A third study by the same group was focused on quantifying interionic distances in aggregates.^226^ By analyzing both resonant and nonresonant vibrational energy transfer in 10 M KSCN solutions, Bian et al. validated the Fermi golden rule predictions and estimated an average separation of ∼ 4.0 Å between SCN^–^ ions. The close agreement between energy-transfer-derived distances in solution and those in crystalline KSCN led to the conclusion that highly concentrated solutions form clusters that are structurally analogous to their solid-state counterparts.

In a later study by Bian et al., the balance between ionic clustering and direct ion–water interactions was probed by vibrational energy-transfer among thiocyanate ions and compared to the changes in rotational dynamics, as a function of cation identity.^180^ The authors observed a clear trend in rotational times for the alkali metals, Li^+^ > Na^+^ > K^+^ ≈ Cs^+^, consistent with decreasing cation charge density and hydration strength. In particular, for smaller cations, such as Li^+^ and Na^+^, the authors concluded that the strong interaction with water creates rigid and heterogeneous local environments, as well as geometrically constrained ionic clusters, which suppress the rotational motions of both water molecules and anions. In contrast, larger and more weakly hydrated cations (K^+^ and Cs^+^) perturb the water hydrogen-bond network less severely, resulting in nearly viscosity-limited rotational dynamics.

Finally, Lewis et al. extended these studies to LiTFSI-water electrolytes across dilute to water-in-salt electrolytes (WISE) regimes, using the asymmetric SO_2_ stretch of TFSI^–^ as an internal probe (Figure 8d,e).^89^ The 2DIR spectra exhibited strong homogeneous broadening and pronounced cross peaks at concentrations near 2 M, consistent with direct interaction between Li^+^ and TFSI^–^, and consequently, the formation of abundant contact ion pairs and higher-order ionic clusters, as seen by the coordination number. A reduction of 1 order of magnitude in the intermolecular vibrational energy transfer time constants was also observed upon increasing salt concentration, signaling the emergence of a percolating and ion-rich network in which anions are in close contact. Molecular dynamics (MD) simulations corroborated this interpretation and revealed extended ionic domains more reminiscent of ILs than conventional aqueous electrolytes.

#### 5.1.4 Ionic Aggregation in Organic Electrolytes

Ionic aggregation in organic electrolytes has also been extensively investigated using 2DIR spectroscopy. However, the spectroscopic signatures used to identify aggregation differ markedly from those in aqueous systems. In water, ionic aggregation has often been inferred from the growth of cross peaks driven by vibrational energy transfer between neighboring ions, while studies in organic solvents frequently revealed the presence of higher-order aggregates through the appearance of new spectral bands at high salt concentrations. These additional features, observed in both the SCN^–^ nitrile stretch and solvent vibrational modes such as the DMC carbonyl stretch, are distinct from those associated with simple contact ion pairs (CIPs). Notably, all the studies suggest that ionic aggregation occurs at substantially lower concentrations than in aqueous electrolytes, reflecting the limited solvation ability of organic solvents. Weak ion-dipole interactions, significant dispersion forces, and reduced dielectric screening allow Coulombic attraction to dominate, thereby promoting both CIP formation and the emergence of larger ionic clusters.

The influence of solvent dielectric constant on ion association was demonstrated by Ghosh et al. in a study of thiocyanate salts dissolved in ethers (Figure 9a,b).^211^ In low-dielectric solvents, such as THF, NaSCN forms stable CIPs and dimers at concentrations as low as ∼ 0.5 M. The authors did not observe chemical exchange on picosecond timescales for CIPs due to strong Coulombic interaction with minimal screening. In contrast, KSCN and NH_4_SCN under similar conditions showed an additional high-frequency spectral feature from the formation of higher-order ionic aggregates. The spectra were interpreted as arising from multiple coexisting ionic species, whose exchange dynamics occur on two distinct timescales: rapid interconversion between aggregates and CIPs/dimers, and slower chemical exchange between CIPs and dimers. Interestingly, the authors also found that the spectral diffusion dynamics of the neat solvent is comparable to the aggregate interconversion rate, suggesting that solvent fluctuations may mediate speciation exchange even in low-dielectric solvents.

**Figure 9:**
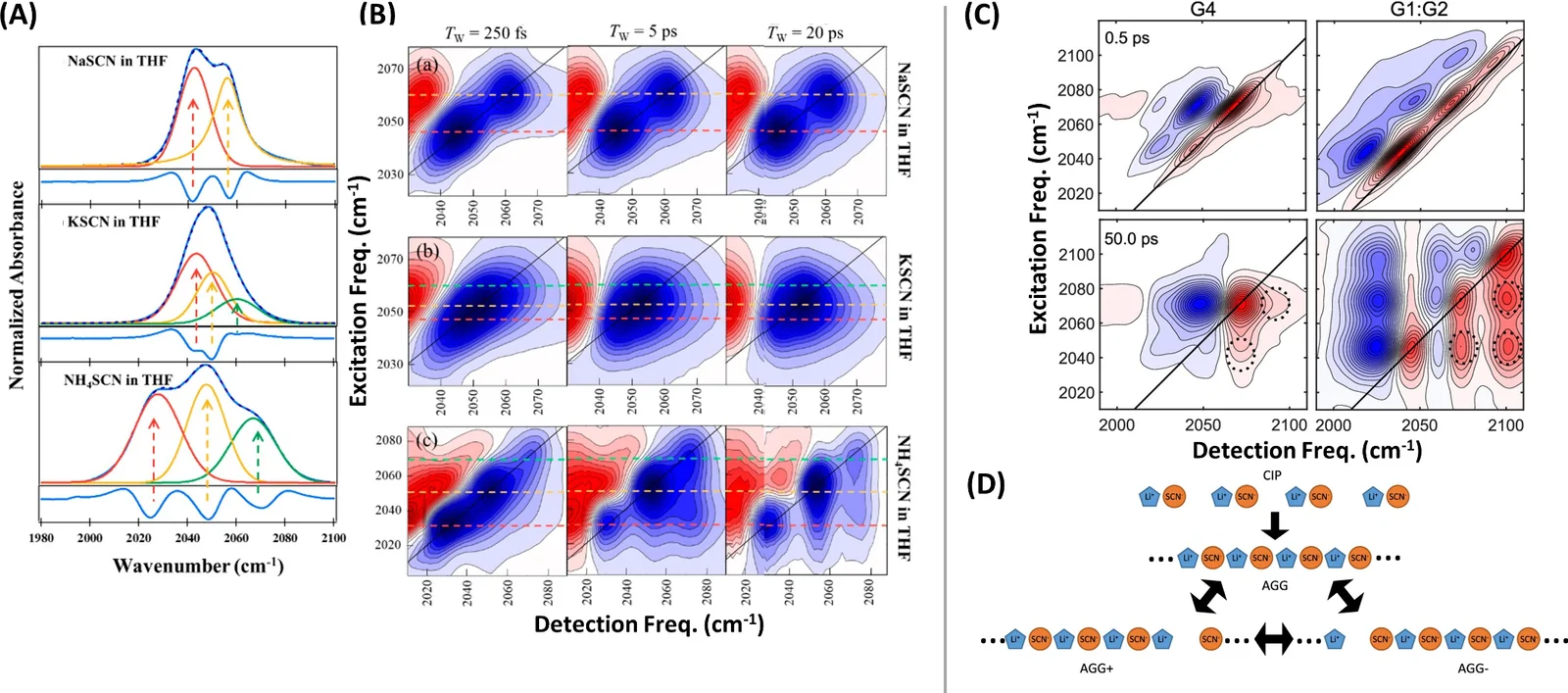
Ionic aggregation in organic solvents. Left panel: Different thiocyanate salts in tetrahydrofuran (THF). (A) IR absorption spectra of NaSCN, KSCN, and NH_4_SCN in THF. The model peak represents the ion pair dimer (red), the contact ion pair (yellow) and the blue-shifted peak (green). (B) 2DIR spectra of NaSCN, KSCN, and NH_4_SCN at different waiting times. The dashed lines denote the frequency positions of the underlying peaks. Adapted with permission from ref.^211^ Copyright 2022 American Chemical Society. Right panel: LiSCN at high concentration in glymes. (C) 2DIR spectra in the CN stretch region of LiSCN in the tetraglyme (G4) solvate ionic liquid and monoglyme-diglyme (G1:G2) high concentration electrolyte for *T* = 0.5 and 50.0 ps. The dashed circle shows the locations of the cross peaks. (D) Cartoon representation of the proposed chemical equilibrium between the CIPs, neutral ionic aggregates (AGG), and charged aggregate (AGG^±^) species of LiSCN in glymes. Adapted with permission from ref.^26^ Copyright 2024 American Chemical Society.

A systematic study of the ionic aggregation in organic electrolytes was provided by Nachaki et al.^26^ The authors examined LiSCN in pure and mixed glyme solvents over a wide concentration range, spanning across conventional electrolytes, solvate ionic liquids, and high-concentration electrolytes (Figure 9c,d). As salt concentration increased, the formation of ionic aggregates became prominent. A comparison between pure and mixed glymes revealed that solvent identity plays a relatively minor role in ionic speciation at low concentrations, but becomes critical in the high concentration regime, where longer glymes more effectively support the extended coordination environments required for ion transport. More importantly, the authors showed that all ionic species exchange thermally on picosecond timescales, indicating that rapid interchange among ionic species is an important feature occurring in highly concentrated Li^+^ organic electrolytes.

Finally, Fulfer et al. demonstrated that solvent vibrational modes themselves are good reporters of ion pairing and aggregation in carbonate-based electrolytes.^201^ Using the carbonyl stretch of DMC to probe LiPF_6_/DMC solutions, the authors found that free ions and SSIPs dominate at typical battery electrolyte concentrations of ∼ 1 M, whereas ionic aggregates become prevalent only at higher salt concentrations (∼3.5 M). The 2DIR spectra exhibited two distinct classes of cross peaks: one arising from vibrational coupling among coordinated carbonyl groups associated with free Li^+^ or SSIPs, and another originating from coupling between carbonyl modes belonging to distinct solvation environments. In particular, the authors observed that only higher-order ionic aggregates exhibited vibrational couplings consistent with their spectral signature, demonstrating that 2DIR spectroscopy can detect complex ionic assemblies in nonaqueous electrolytes by probing either the ions or the solvent molecules.

### 5.2 Solvent Structure and Dynamics in Electrolytes

#### 5.2.1 Effects of Ions on Water Structure

Understanding how hydrated ions modify the structure and dynamics of liquid water remains central to many scientific fields. The ability of water to rapidly reorganize its hydrogen-bond structure in less than a few picoseconds underlies many important phenomena, such as solvation, transport, and chemical reactivity. However, the presence of simple salts induces large perturbations in the water hydrogen-bonded network, challenging the classical notions of “structure-making” and “structure-breaking” ions. Ultrafast infrared spectroscopies, mostly 2DIR spectroscopy, have predominantly studied this area by directly probing the effect of different ions on the hydrogen-bond exchange, solvent motions, and orientational dynamics. Across a wide range of monovalent and multivalent electrolytes, a consistent picture has emerged: ions slow the restructuring of water hydrogen-bond networks by a modest factor, while introducing persistent local heterogeneities in water configurations.

Early 2DIR studies by Park et al. established a benchmark for understanding the effects of monovalent salt on water using highly concentrated NaBr solutions.^146,149–151^ Even at concentrations up to 6 M, the solution dynamics was governed by the making and breaking of hydrogen-bonds between water molecules and ions, in a manner analogous to pure water. Moreover, the OD stretch band of HOD in the salt solutions showed a significant blue shift relative to HOD in pure water, suggesting a large change in the hydrogen-bond structure. Finally, the FFCF of the OD stretch displayed biexponential decay in both pure water and highly concentrated NaBr solutions. The authors attributed the fast component (hundreds of femtoseconds) to local hydrogen-bond length fluctuations and the slow component (of a few picoseconds) to complete randomization of the hydrogen-bond network. Hence, measurements of spectral diffusion and orientational dynamics demonstrated that both dynamical components progressively slowed with increasing salt concentration, suggesting a common molecular origin for the two processes. Moreover, despite the slowdown, the change in the dynamics was limited to roughly a factor of 3 in both cases.

Another foundational observation for the field was reported in 2008 by Moilanen et al., who identified two distinct spectral populations of water molecules (i.e., anion-bound and water-bound) in a highly concentrated NaBF_4_ electrolyte undergoing chemical exchange on a picosecond timescale.^152^ This finding was particularly significant because it provided the first direct spectroscopic evidence that, in concentrated electrolytes, water does not behave as a homogeneous continuum medium, but instead exists in well-defined and dynamically interconverting microscopic environments. Further, the ability to resolve and quantify exchange between local hydration motifs represented a major advance, which linked molecular-scale structure directly to ultrafast dynamics (more about this topic is discussed in Section 5.3). Building on this insight, subsequent studies by Park et al. and Son et al. with salts such as NaClO_4_ ^215^ and NaPF_6_ ^220^ showed that this phenomenon was not unique to a single electrolyte (Figure 10a,b), and established the universal molecular framework of “anion-bound” and “water-bound” water species exchanging in few picoseconds.

**Figure 10:**
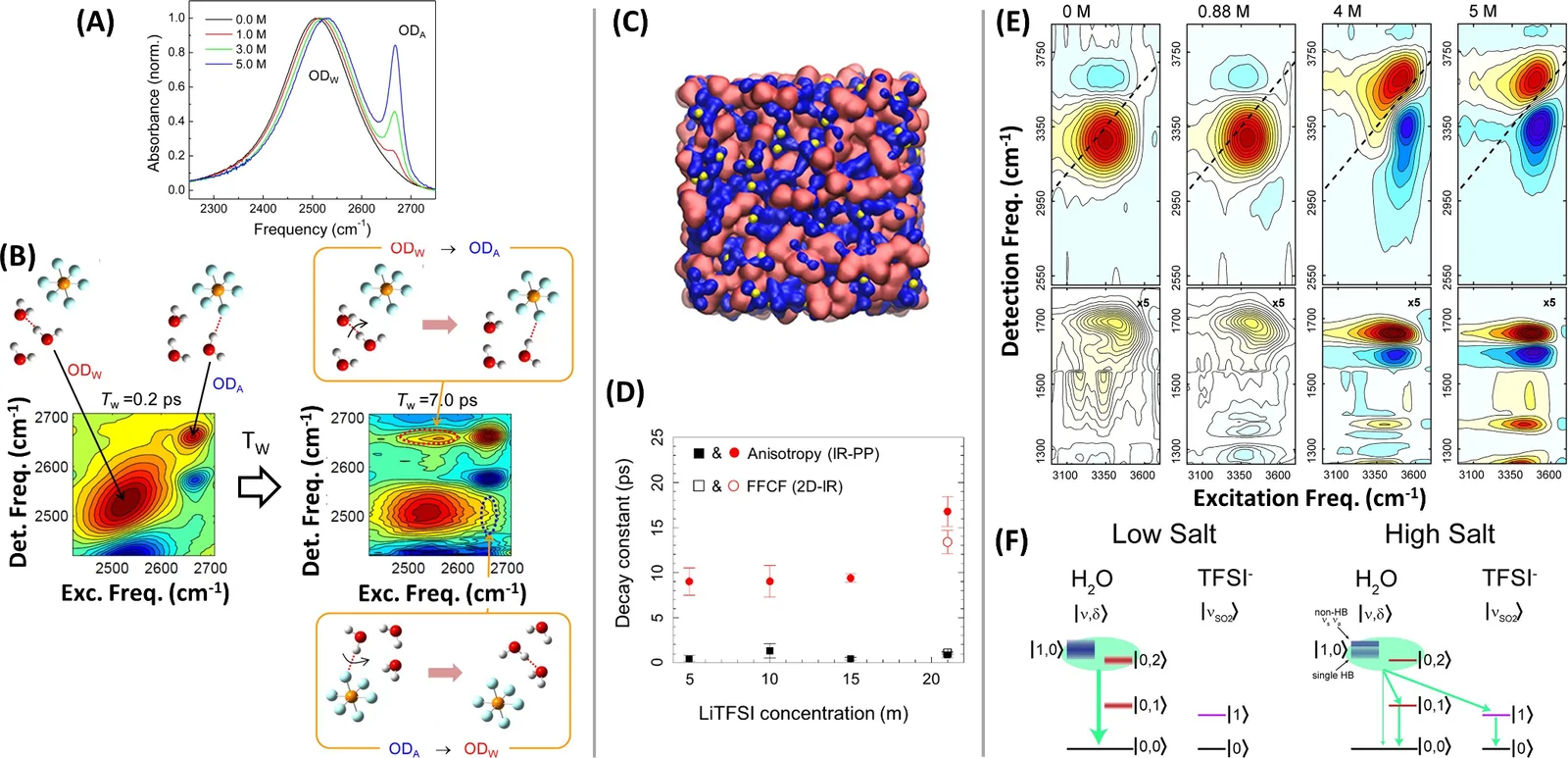
Ionic aggregation in water. Left panel: Aqueous NaPF_6_ solutions. (A) FTIR spectra of aqueous NaPF_6_ solutions at different concentrations in 8% HOD in H_2_O, where OD_W_ is OD group bound to water and OD_A_ correspond to OD group bound to an anion. (B) Schematic illustration of hydrogen-bond exchange dynamics observed in the waiting time evolution of 2DIR spectra for aqueous 5.0 M NaPF_6_ solution. The upper left cross peak is the hydrogen-bond exchange from OD_W_ to OD_A_, while the lower right cross peak is the inverse process. Adapted with permission from ref.^220^ Copyright 2013 American Chemical Society. Center panel: high concentrated LiTFSI aqueous solutions. (C) A slab of an MD simulation box of a 21 m LiTFSI electrolyte solution, where blue and red regions correspond to water and ion aggregate, respectively. Adapted with permission from ref.^262^ Copyright 2020 American Chemical Society. (D) Characteristic constant of the rotational correlation functions from a biexponential function having a fast (solid black squares) and a slow (solid red circles) component as a function of LiTFSI concentration. The biexponential decay constants of the nodal line slope (FFCF) are also included as open symbols. Adapted with permission from ref.^143^ Copyright 2018 American Chemical Society. Right panel: Vibrational relaxation as a function of LiTFSI concentration. (E) 2DIR spectra for the kinetic intermediate as a function of LiTFSI concentration. (F) Schematic ladder diagrams illustrating the changes in vibrational relaxation channels with increasing LiTFSI content. Adapted with permission from ref.^142^ Copyright 2022 American Chemical Society.

Studies largely focused on how monovalent anions modulate water dynamics. However, Giammanco et al. revealed a far richer and more subtle molecular picture in which both cation and anion identities play important roles in shaping water solvation.^203^ By systematically examining electrolytes containing divalent cations paired with a range of different anions, the authors uncovered two insights.^203^ First, it was demonstrated that local observables, such as OD stretch frequency shifts and vibrational lifetimes, are governed by the anion-associated and bulk-like water populations. In contrast, orientational relaxation and the slow component of spectral diffusion of the OD stretch did not report on either population independently. Instead, these dynamics reflected collective motions that span both environments, demonstrating that long-range hydrogen-bond rearrangement transcends simple population partitioning. Second, the study provided evidence that cation identity itself could control key aspects of dynamics in aqueous electrolytes. For example, the authors showed that at similar concentrations, MgBr_2_ exhibits significantly slower structural dynamics than NaBr. This effect, however, was not observed to be universal, since MgSO_4_ and Na_2_SO_4_ did not show no appreciable differences. Thus, the authors concluded that the influence of the cation charge density on solvent dynamics is not intrinsic to the cation, but instead emerges through its interplay with the charge density of the counter-anion, as well. Reorientational dynamics also showed dependence on the identities of both the cation and anion, rather than on either species alone, and progressively slow down with increasing salt concentration. These findings fundamentally revised the prevailing anion-centric framework of aqueous electrolytes.

Over the past decade, the focus of 2DIR studies has shifted toward superconcentrated electrolytes. In such systems, the molecular framework developed for dilute and concentrated electrolytes breaks down, raising fundamental questions about how hydrogen-bond networks reorganize and whether traditional distinctions between bulk-like and ion-associated water exist. Experiments and MD simulations by Lim et al. on 21 molal LiTFSI initially proposed that, in these extreme conditions, ions form a three-dimensional ion network intertwined with a nanoscopic water hydrogen-bond network.^143^ In this molecular picture, the ionic network arrangement has bulk-like water channels with an average diameter of ∼ 1.2 nm, and interfaces where water molecules exhibit distinct dynamical behavior. The experimental results were interpreted as the rotational relaxation of water molecules in water-in-salt electrolytes (WISE), consisting of a fast “wobble-in-a-cone” motion, followed by much slower, large-amplitude rotational jumps between anion sites (Figure 10d). Notably, no chemical exchange between the two dominant water populations was observed, suggesting that water–anion interactions are either exceptionally strong or that water molecules are spatially confined near ion aggregates. The same group later developed a new force field designed to reproduce the target properties of the solution over a larger concentration range (1–21 m).^262^ These simulations revealed a lower degree of segregation between the water and the ionic network in WISE, where only 10% of water molecules are purely solvated by other water molecules (Figure 10c).

Later, experimental studies from other groups also indicated that in WISE, most water molecules interact directly with ions and that bulk-like water is largely absent. Roget et al. showed that in aqueous LiCl, the extremely high salt concentrations cause a significant slowdown of the dynamical components associated with local fluctuations and the complete structure rearrangement process of the water network, compared with bulk water and dilute electrolyte solutions.^174^ Complementary vibrational anisotropy measurements and MD simulations also revealed increasingly constrained angular reorientation imposed by the ions, with the remaining water–water hydrogen-bonded network becoming stronger, on average, but also more spatially restricted. Consistent with this molecular framework, Lewis et al. investigated vibrational energy transport in LiTFSI electrolytes by monitoring the water OH stretch from concentrated to WISE (Figure 10e,f).^142^ The authors observed that bulk-like water behavior persists at moderate concentrations (≤10 m), but at ≥ 20 m, the water hydrogen-bond network fragments into isolated pools in which water molecules interact almost exclusively with the anions. In another experiment using broadband 2DIR spectroscopy, the same authors showed that the strong vibrational coupling characteristic of pure water is replaced by multistep relaxation pathways involving both intramolecular solvent modes and anion vibrations. These results confirmed the rupture of the bulk water structure in the superconcentrated regime.

#### 5.2.2 Effects of Salts on Organic Solvents

Contrasting aqueous electrolytes, the influence of ionic salts on the collective dynamics of organic solvents has received comparatively little attention. While ion–solvent interactions within the primary solvation shell of cations, like Li^+^ and Na^+^, are relatively well understood (see Section 5.4), only a limited number of studies have discussed how ions affect the dynamics of the unbound, or “free,” solvent population through 2DIR measurements of spectral diffusion.

An early study by Fulfer et al. demonstrated that the addition of LiPF_6_ significantly slows the dynamics of carbonate solvent molecules that are not coordinated to Li^+^.^85^ These observations suggested that ions influence solvent structure well beyond the first Li^+^ solvation shell by promoting long-range structural ordering. In a subsequent study, this behavior was observed across a series of linear organic carbonates with varying alkyl chain lengths.^199^ In the absence of salt, solvent size has little influence on the decorrelation times of solvent dynamics, with all values on the order of a few picoseconds. Upon addition of LiPF_6_, however, the FFCF correlation times of the free solvent nearly doubled in the studied systems, indicating that even noncoordinated molecules observe a more ordered environment when ions are present. A similar slowdown was observed in urea-based electrolytes by Chen et al.^263^ Despite the planar geometry and conformational rigidity imposed by amide bonding in the urea, the introduction of Li^+^ slowed the dynamics of the noncoordinated solvent molecules. In both carbonate- and urea-based systems, the slowdown in the dynamics was attributed to a combination of long-range electrostatic interactions, anion-solvent coupling, and constraints propagated from the Li^+^ solvation structure. Overall, these studies established that lithium salts drive extensive structural reorganization in molecular electrolytes that extend far beyond the ions immediate solvation shell.

More recently, Miao et al. investigated the role of cosolvents and nonsolvating additives in sodium-ion electrolytes (Figure 11).^221^ In NaPF_6_ solutions in dimethyl carbonate (DMC), the authors observed that the dynamics of free solvent molecules remain largely unchanged when mixed with the nonsolvating fluorinated ether, indicating that the additive does not significantly restrict the motions of the organic carbonate. In contrast, the use of propylene carbonate as a cosolvent slowed down the DMC dynamics due to its stronger intermolecular interactions and higher viscosity. The authors also observed that regardless of the additive, both cosolvents increase the structural inhomogeneity of DMC molecules coordinated to Na^+^, indicating a more heterogeneous local environment. The authors concluded that both cosolvents and additives amplify structural heterogeneity of the solvent in both free and ion-coordinated carbonate populations.

**Figure 11:**
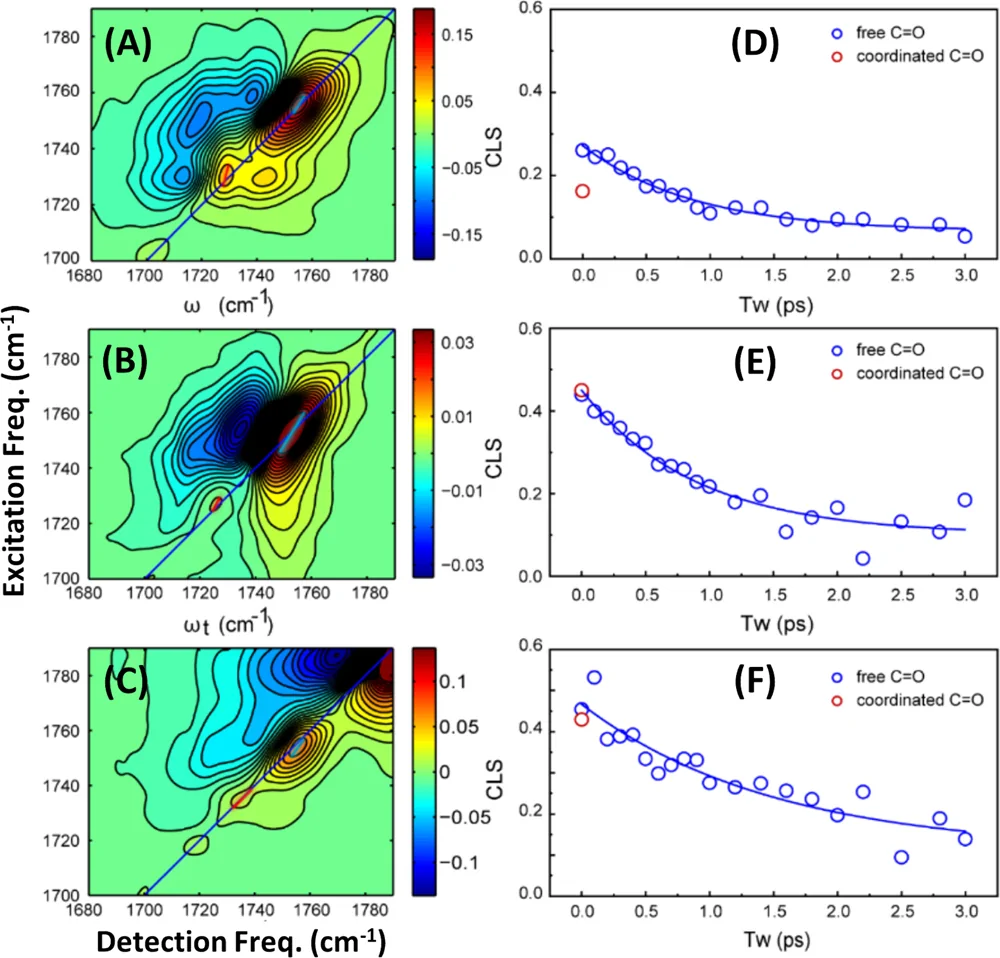
2DIR spectra of 1 M NaPF_6_ in the carbonyl stretching region of (a) dimethyl carbonate (DMC), (b) dimethyl carbonate/1,1,2,2-Tetrafluoro-2,2,2-trifluoroethoxy ethane, and (c) dimethyl carbonate/propylene carbonate at *T* = 0.30 ps with center lines marked for free DMC (blue circle) and Na^+^-coordinated DMC (red circle) in each case. (d-f) CLS dynamics of the correspondingly marked carbonyl stretching peaks, where only initial values were extracted for the Na^+^-coordinated DMC (red circle). Adapted with permission from ref.^221^ Copyright 2025 American Chemical Society.

### 5.3 Solvation Shell Dynamics of Aqueous Electrolytes

In previous sections, it was established that ions perturb the hydrogen-bond network of water and its ultrafast dynamics. Here, the more intricate aspects of electrolyte behavior, such as the structure and dynamics of the ion solvation shells, are presented.

Over the past several decades, a wide range of experimental and theoretical studies have examined how water molecules, anions, and cations respond to one another. All these investigations revealed that hydration dynamics are governed by the interplay between short-range coordination chemistry and longer-range solvent organization. In the following sections, insights from studies of ion solvation are reviewed.

#### 5.3.1 Water Perspective

As mentioned in previous sections, water molecules in salt electrolytes exist in well-defined and dynamically interconverting microscopic environments as demonstrated in the initial studies of aqueous solutions of NaBF_4,_ ^152^ NaClO_4_,^215^ and NaPF_6_.^220^ However, the molecular description of anion hydration and the mechanism through which the exchange of water molecules move in and out of the hydration shell was explored by the Gaffney group in 2010 and 2011.^216,217^ Ji et al. demonstrated that cross peak signals arising from water molecules undergoing chemical exchange between “free” and “anion-bound” states exhibited distinctly different polarization dependence from the populations that remain in a single H-bond configuration (Figure 12).^216,217^ Analysis of the mechanism showed that the hydrogen-bond rearrangement proceeds via large, discrete angular jumps, rather than through diffusive reorientation. This water exchange mechanism explains the three distinct timescales associated with motions of hydroxyl groups: (i) diffusive motion of intact OH···O hydrogen-bonds, (ii) large angular jumps associated with hydrogen-bond exchange, and (iii) restricted wobbling in a cone motion about the hydrogen-bond axis. Thus, this mechanism provided a complete molecular picture of water dynamics, in which rotational diffusion and hydrogen-bond rearrangement are intrinsically coupled, giving rise to a pronounced dynamical heterogeneity in concentrated electrolyte solutions.

**Figure 12:**
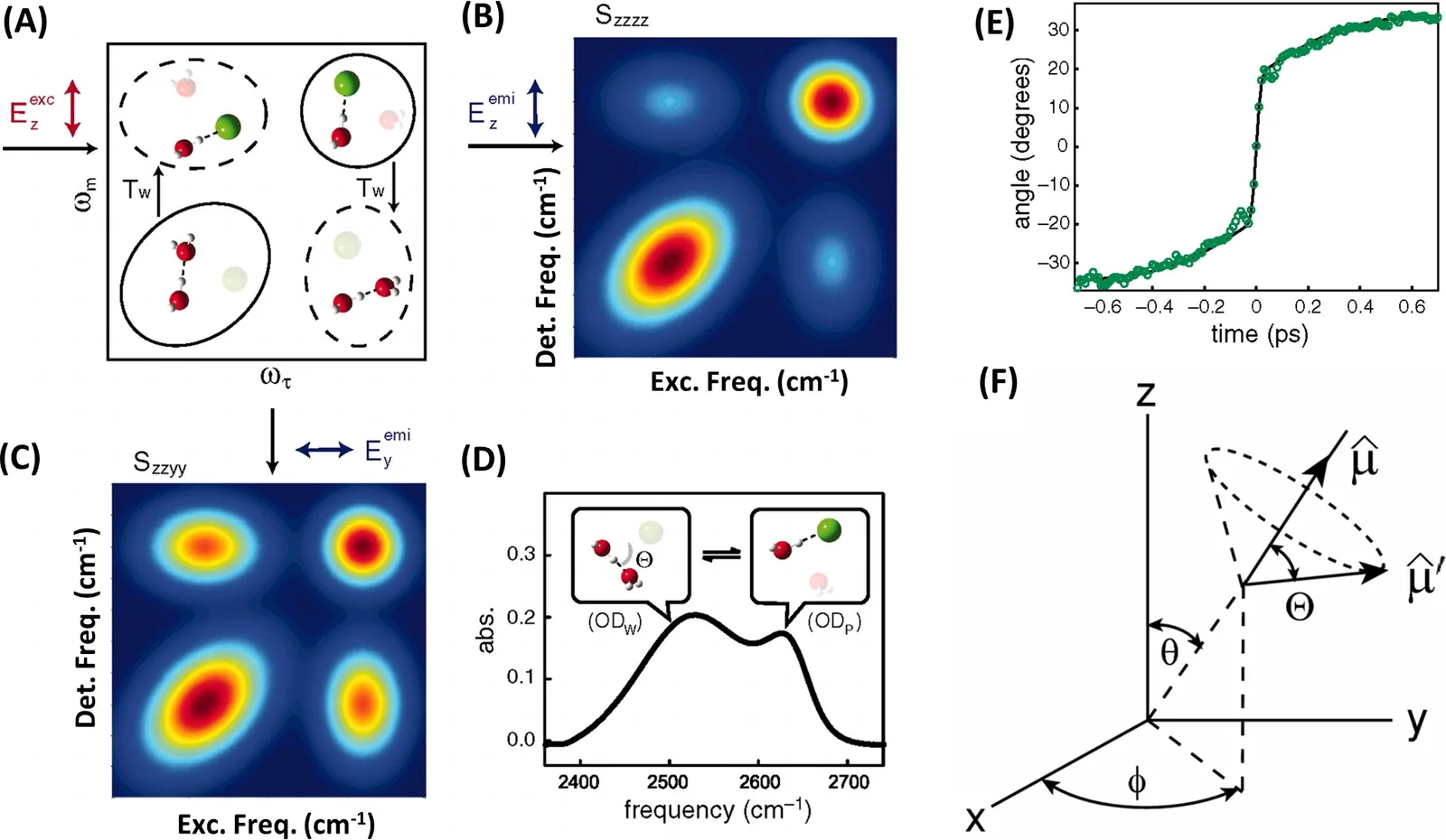
Interpretation of polarization-selective 2DIR signals. The H-bond configurations appear schematically, with the green spheres representing perchlorate ions. (A) Linearly polarized excitation pulses selectively excite vibrational dipoles oriented parallel to the z direction. After a waiting time, vibrationally labeled hydroxyl groups that have not changed hydrogen-bond configuration contribute to the diagonal component of the 2DIR signal. Vibrationally labeled hydroxyl groups that have undergone hydrogen-bond exchange generate cross peaks. If the exchange proceeds through large angular jumps, the hydroxyl groups associated with the cross peaks will show markedly different polarization dependence than those along the diagonal. 2DIR with all parallel polarizations (B) and with cross-polarized pulses (C). (D) FTIR absorption spectrum for 6 M NaClO_4_ dissolved in H_2_O containing 5% HDO water. The low frequency peak corresponds to the OD stretch hydrogen-bonded to other water molecules (OD_W_) and high frequency peak corresponds to the OD stretch hydrogen-bonded to a perchlorate ion (OD_P_). (E) MD simulation of the jump angle for hydrogen-bond exchange between the OD_P_ and the OD_W_ configurations for aqueous 6 M NaClO_4_ solution. Black line is the data fit to a sum of two error functions. Reproduced from Ji et al., Science, 10.1126/science.1187707 [2010], AAAS^216^ (F) Schematic of the angular jump in the laboratory frame of reference (x, y, z). Reprinted from Ji, M.; Gaffney, K. J. *Orientational Relaxation Dynamics in Aqueous Ionic Solution: Polarization-Selective Two-Dimensional Infrared Study of Angular Jump-Exchange Dynamics in Aqueous 6 M NaClO4*. *Journal of Chemical Physics* **2011**, 134 (4), 044516. 10.1063/1.3530783, with the permission of AIP Publishing.^217^

Complementary insights were later obtained by the Fayer group through OD stretch of isotopically diluted water in systems sharing a common anion: TFSI^–^ (bis­(trifluoromethanesulfonyl)­imide anion). Giammanco et al. compared aqueous LiTFSI with an aqueous EMIMTFSI ionic liquid and observed that, while vibrational lifetimes and restricted reorientation dynamics differed only slightly, spectral diffusion was substantially slower in the LiTFSI solution.^186^ This pronounced difference in dynamics was interpreted as Li^+^ salts imposing stronger configurational constraints on nearby water molecules, than the ions comprising the ionic liquid (IL). Further analysis of the 2DIR spectral lineshapes revealed that the aqueous ionic liquid exhibits a larger homogeneous contribution, consistent with faster and motionally narrowed structural fluctuations. These results showed that water plays fundamentally different roles in these two liquids and highlight the importance of both cation identity and mesoscale ionic organization in defining the configurations and dynamical heterogeneity accessible by water molecules in each system.

#### 5.3.2 Cation Perspective

A complete description of solvation shell dynamics requires probing solvation from both the anion and cation perspectives. In practice, however, direct experimental access to cation solvation in water electrolytes has been far more limited. This asymmetry arises primarily from the directionality of water–ion interactions and from the scarcity of intrinsic cation vibrational reporters.

Anions are solvated through directional hydrogen-bonds, which directly involve OH groups of water, making the OH stretch a sensitive probe. In contrast, cation solvation is dominated by coordination of water molecules through their oxygen atoms, with the OH groups oriented away from it. Consequently, fluctuations in cation hydration shells only weakly perturb OH vibrational frequencies, rendering them difficult to detect spectroscopically. Thus, the water orientation in cation solvation added to the lack of intrinsic vibrational modes in cations have severely limited the studies to a few examples using 2DIR spectroscopy.

To date, guanidinium (Gdm^+^) remains as the only cation that has been successfully studied as a 2DIR reporter of aqueous solvation. Vorobyev et al. used Gdm^+^ to investigate solvation-induced symmetry breaking of the cation by monitoring a pair of nearly degenerate vibrational modes involving coupled CN_3_ asymmetric stretches and NH_2_ scissoring motions (Figure 13).^127^ In both water and water/glycerol mixtures, these vibrational modes split due to solvation induced geometric distortions, but most prominently in the viscous aqueous glycerol solution. The 2DIR spectra revealed cross peaks corresponding to vibrational energy transfer on a subpicosecond timescale, which the authors attributed to fluctuations in the hydrogen-bond network surrounding the planar Gdm^+^ ion. Specifically, solvent-driven wagging motions of the terminal NH_2_ groups modulate the vibrational frequencies and lift the near degeneracy of the modes. More importantly, this work established that solvent-induced symmetry breaking of molecular cations can be used as a powerful spectroscopic variable for probing the collective fluctuations of the surrounding solvent that are otherwise difficult to access.

**Figure 13:**
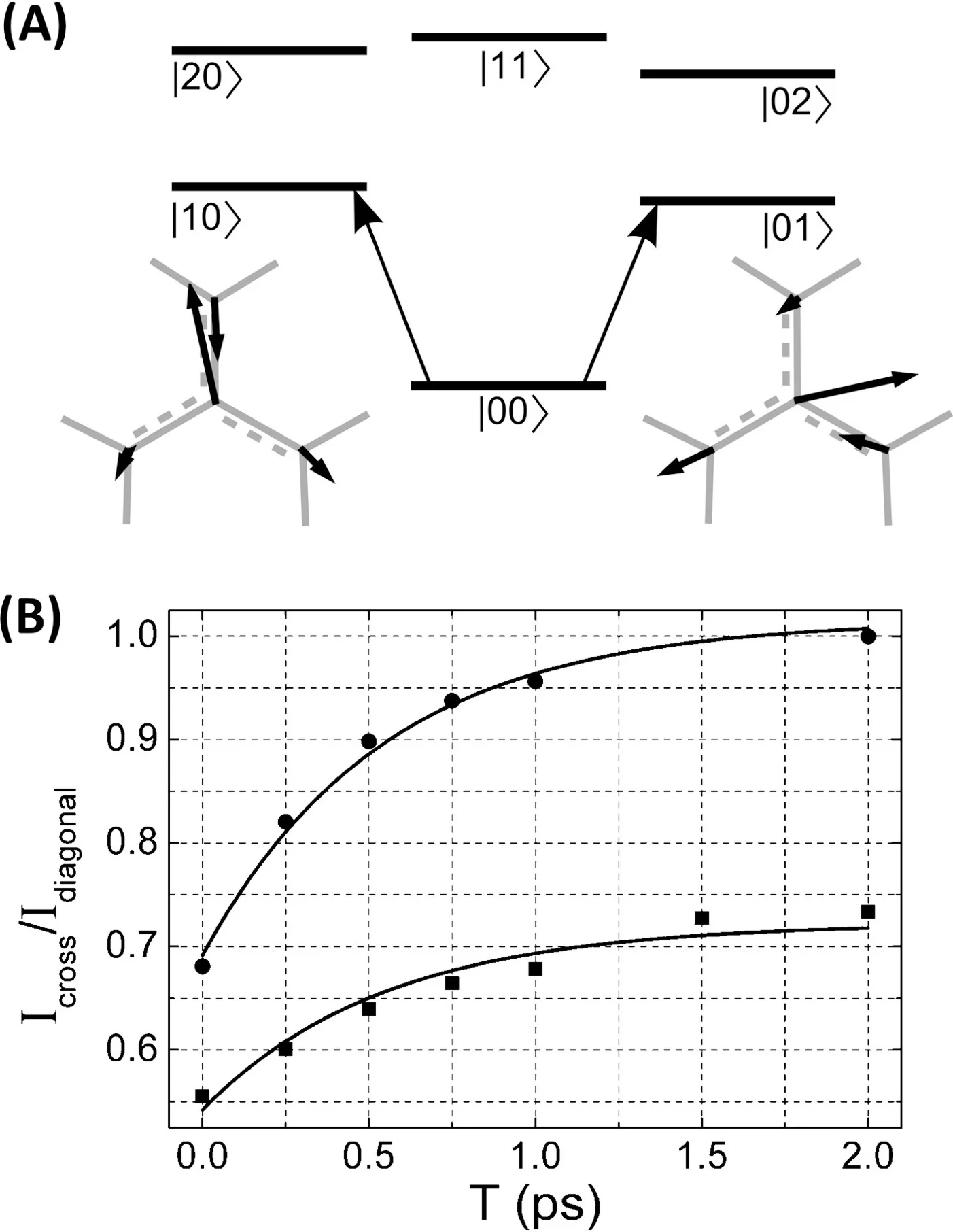
Guanidinium ion (Gdm^+^) solvation in water. (A) Energy diagram for the separated vibrational normal modes. The displacements of the atoms of the CN_3_ group of Gdm+ for the two degenerate components of the 1600 cm^–1^ normal mode, with the atomic displacements illustrating the orthogonal transition dipoles. (B) Cross peak dynamics showcase by the ratio of the amplitudes between the cross peak at two different polarizations XXXX (squares) and XXYY (circles). Solid curves is the modeling of the data. Adapted with permission from ref.^127^ Copyright 2010 American Chemical Society.

#### 5.3.3 Anion Perspective

The cation solvation remains experimentally difficult to probe, but anion solvation offers a more accessible and diverse framework for investigating ion–solvent interactions across a wide range of chemical environments and chemical motifs. Thus, among electrolyte constituents, anions have been extensively studied, enabling a systematic investigation of anions-water interactions through vibrational coupling and hydrogen-bond dynamics using 2DIR spectroscopy.

The modern framework for interpreting anion solvation dynamics was established by Hamm, Lim and Hochstrasser in 1998 through femtosecond infrared photon echo studies of azide in water (Figure 14a).^106^ These experiments revealed characteristic dynamics of FFCF with two timescales: a subpicosecond component and a slower picosecond component. At the time, the fast component was tentatively assigned to motions of the negatively charged oxygen atoms of water interacting with the positively charged central atom of the N_3_^–^ ion, while the slower component was attributed to the residence time of hydrogen-bonds between the anion and surrounding water molecules.

**Figure 14:**
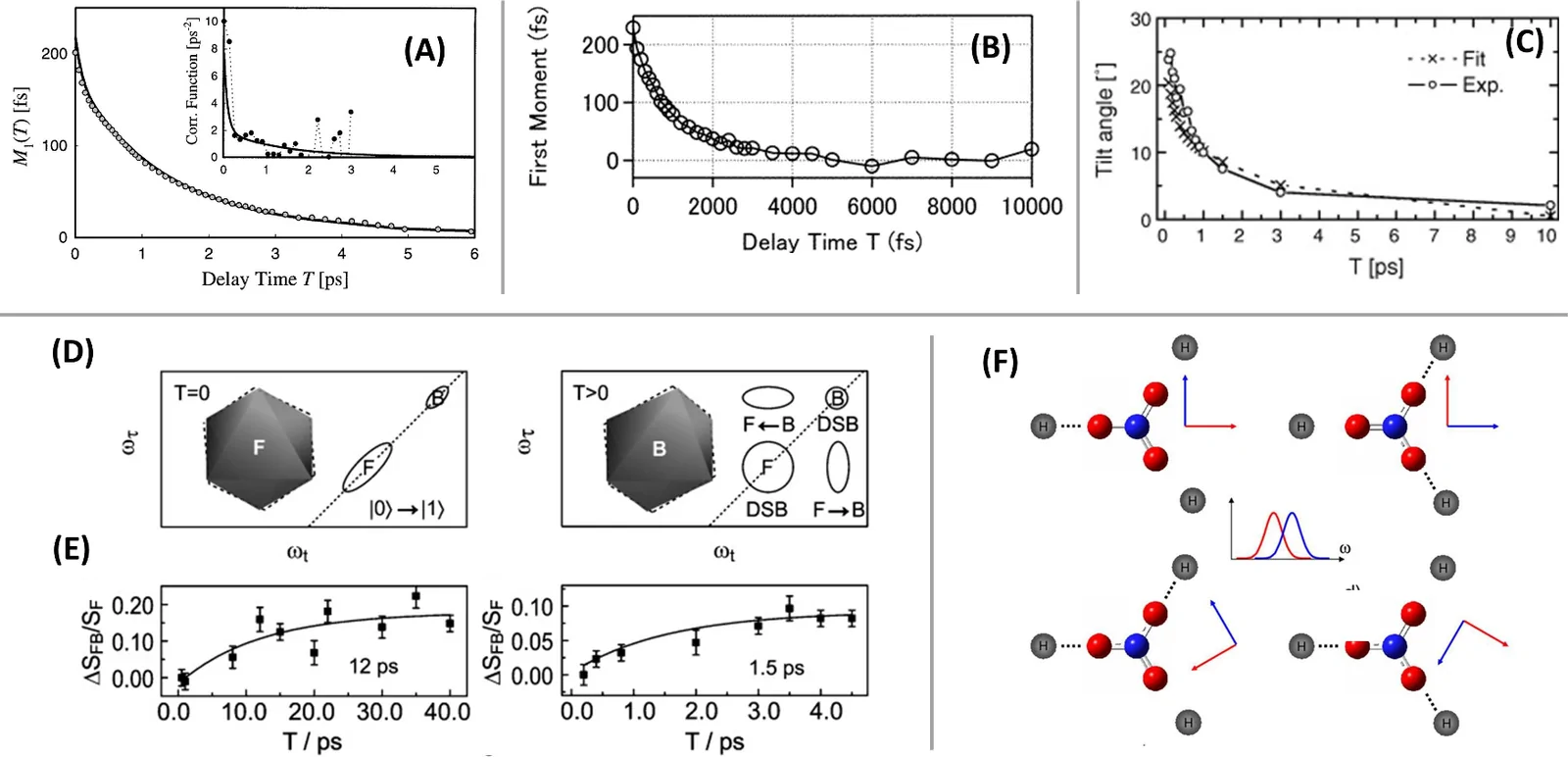
Anion hydration dynamics. Top left panel: (A) The first moment of spectral diffusion for azide ion in D_2_O. The inset shows the correlation function obtained from the experimental absorption spectrum (dots). Reprinted figure with permission from Peter Hamm, Manho Lim, and Robin M. Hochstrasser, *Phys. Rev. Lett*. **81**, 5326–5329 (1998). Copyright (1998) by the American Physical Society.^106^ Top center panel: (B) Spectral diffusion as the first moment of the peak for SCN^–^ in D_2_O. Adapted with permission from ref.^225^ Copyright 2004 American Chemical Society. Top right panel: (C) The tilt of the 2DIR peak line-shape as a function of T for CN^–^ in D_2_O. Reprinted from *Chemical Physics*, Vol. 341, M. Kozinski, S. Garrett-Roe, and P. Hamm, “Vibrational spectral diffusion of CN^–^ in water,” pp. 5–10, Copyright (2007), with permission from Elsevier.^224^ Lower left panel: Symmetry breaking mechanism in water solutions of [Co­(CN)_6_]^3–^. (D) Illustration of the 2DIR signature (0→1 transition only) of the solvent-mediated symmetry-breaking dynamics of the F^–^ and B^–^ species of [Co­(CN)_6_]^3–^. (E) Experimental off-diagonal signal-growth dynamics of cross peaks in glycerol and ethylene glycol, respectively. Adapted with permission from ref.^103^ Copyright 2013 John Wiley and Sons. (F) Schematic representation of different solvated nitrate configurations. Transitions between these configurations caused by solvation shell fluctuations explain the observed cross peak growth and simultaneous decay of the diagonal peak anisotropy due to reorientation of their transition dipole moments. Dipole moment orientations are indicated by red and blue arrows, which correspond to lower- and higher-frequency transitions, respectively. Adapted with permission from ref.^111^ Copyright 2013 American Chemical Society.

Subsequent studies by Ohta et al. and Tayama et al. reported comparable FFCF timescales for the CN stretch, across a broad range of molecular and metal-complex anions, including Fe­(CN)_6_^4–^, SCN^–^, and Ru­(CN)_6_^4–^.^104,225^ These results demonstrated that the characteristic timescales of spectral diffusion are remarkably universal in both systems with degenerate and nondegenerate vibrational modes. At the same time, the relative amplitudes of the FFCF components were found to depend on solute identity and the surrounding solvent environment. Further investigations using isotopic substitution revealed that vibrational lifetimes can differ substantially between H_2_O and D_2_O, highlighting the important role of solvent–solute interactions in the vibrational relaxation dynamics.^234^

Throughout the years, the initial assignments of the two FFCF time components were progressively validated by studies incorporating isotopic substitution and the simultaneous tracking of anion and solvent vibrational modes. In 2007, Kozinski et al. measured K^13^C^15^N in D_2_O and found dynamics closely resembling that of azide ions, but with a slight decrease in the picosecond component (Figure 14b,c).^224^ This difference was attributed to more stable hydrogen-bonding around CN^–^, consistent with its more uniform charge distribution relative to N_3_^–^. Complementary measurements by the Hochstrasser group further reinforced the dynamical picture by simultaneously monitoring the dynamics of the anion and its surrounding water molecules. First, Kuo et al. studied the spectral diffusion of the CN stretch of cyanide and the nearby water bend-libration combination mode. The study revealed a shared subpicosecond component assigned to changes in water–ion distance and angular configurations.^205^ Second, the same authors probed the interaction between anions and hydrating water by exciting the azide asymmetric stretch and probing OH stretches of surrounding water molecules with two-color 2DIR spectroscopic experiment.^107^ The authors observed cross peaks with positive frequency correlations that decayed within a few hundred femtoseconds, providing direct evidence for hydration-shell motions that are distinct from bulk water dynamics. Additional work by Tayama et al. showed that the hydrogen-bonding between solute and solvent weakens as the temperature increases over a 70K range, but the subpicosecond and picosecond components of azide spectral diffusion remain largely unchanged.^213^ Combined with IR pump–probe measurements and dielectric relaxation data, these results suggested that the dominant perturbation of the N_3_^–^ antisymmetric stretch originated from localized solvent motions within the hydration shell surrounding the anion. Thus, these studies provided evidence supporting that the fast component of the FFCF reflects local solvent–ion configurational motions, while the slower component represents the stability of the anion hydration shell.

The two-timescale framework successfully rationalized the hydration dynamics of a wide range of anions, while subsequent studies revealed that solvent-induced symmetry breaking provides additional pathways through which water fluctuations can be observed. For example, Tamaya et al. examined the nondegenerate NO stretch of sodium nitroprusside (Na_2_[Fe­(CN)_5_NO]) and found FFCF dynamics comparable to those of the triply degenerate CN stretches of Fe­(CN)_6_^4–^ demonstrating that vibrational degeneracy has a minimal contribution to spectral diffusion of the system.^222^ In contrast, Kuroda et al. showed, in a study of aqueous tricyanomethanide, that solvent-mediated symmetry breaking can strongly influence both anisotropy decay and population transfer.^99^ In this last study, the analysis revealed two distinct hydration mechanisms: rotational anisotropy decay driven by water molecules that break molecular symmetry, and frequency dephasing controlled by collective motions of the anion hydration shell. These ideas were extended further by Yang et al. through studies of hexacyanocobaltate ions ([Co­(CN)_6_]^3–^).^103^ In this work, the authors proposed the coexistence of equilibrium symmetry breaking, arising from distinct and low-symmetry solvation structures, and dynamical symmetry breaking, driven by instantaneous solvent fluctuations (Figure 14d,e).^103^ Later, it was shown that anion charge density also plays an important role in hydration dynamics. Yu et al. compared ferrocyanide ([Fe­(CN)_6_]^4–^) and ferricyanide ([Fe­(CN)_6_]^3–^), and found broader, more inhomogeneous spectra and slightly slower spectral diffusion for the higher-charge anion.^102^ These observations were attributed to stronger and more structured hydrogen-bond interactions, even though the more highly charged anion was also found to perturb the surrounding water network more strongly. Overall, these studies established symmetry breaking as an essential mechanism by which water motions modulate the anion vibrational spectra. However, the degree to which this effect manifested in the degenerate modes was observed to be highly dependent on the system.

Despite the broad applicability of the emerging water solvation framework, in which anions perturb water structure, it was also observed that not all of them exert a strong perturbation. At one extreme are ions whose hydration dynamics are remarkably similar to those of bulk water. Giammanco et al. demonstrated that borohydride exhibits FFCF dynamics nearly indistinguishable from pure water, even at high concentrations.^118^ This behavior was rationalized by the tetrahedral dihydrogen-bonding motif of BH_4_^–^, which seemingly integrates into the water hydrogen-bond network without inducing substantial distortion. At the opposite extreme, several polyatomic anions possess internal degrees of freedom or structural rearrangement pathways that contribute directly to their vibrational dynamics. Kuroda et al.^119^ observed an apparently ultrafast FFCF dynamics for the ^13^C-label trifluoroacetate ion (TFA) compared to its ^12^C-analogue. This unsual behavior was traced to the spectral overlap of carboxylate asymmetric stretch with a combination band in the ^13^C-anion. Using MD simulations, the authors linked the solvation dynamics to interconversion between distinct hydration-shell geometries of the anion. Similarly, Thøgersen et al. showed that nitrate ion possesses a labile hydration shell dominated by ultrafast hydrogen-bond rearrangements and solvent-driven in-plane “pseudo-rotations” (Figure 14f).^111^ Costard et al. also showed that phosphate ions have extremely fast dynamics in the subhundred-femtoseconds range, governed by librational motions of nearby water molecules.^232,233^ Moreover, the same authors observed 2DIR cross peaks arising from coherent vibrational energy transfer between the symmetric and asymmetric PO_2_^–^ stretching modes, even in the presence of much faster solvation dynamics typically associated with dephasing processes.^264^ Another interesting anion solvation shell behavior was observed for acetate. Zhang et al. showed that acetate exhibited solvation dynamics that was largely insensitive to salt concentration, when compared to azide.^202^ Analysis of first-principles molecular dynamics simulations attributed the effect to the rapid methyl rotation, which masked the solvent reorganization. The authors concluded that anion conformation dynamics plays a major role in vibrational dephasing of molecular ions.

Following the development of the molecular framework for anion hydration dynamics under standard conditions, subsequent studies extended these investigations to environments far from them. Czurlok et al. investigated the solvation dynamics of thiocyanate in liquid and supercritical heavy water.^92^ The authors observed the usual biexponential spectral diffusion of aqueous solutions, with the fast component accelerating with increasing temperature and the static heterogeneity diminishing upon transitioning to the supercritical phase. The authors assigned both dynamics to libration-driven hydrogen-bond dynamics in the ion vicinity. They also noted that the spectral diffusion rate exhibited a clear Arrhenius-like temperature dependence, with an activation energy slightly higher than thermal energy.

Finally, recent work has highlighted the substantial impact of multivalent anions on hydration dynamics. Kundu et al. studied the role of divalent cations in the hydration structure and dynamics of the sulfate ion.^114^ Using the asymmetric stretch of the anion, the authors demonstrated that Mg^2+^ induces a marked slowdown of sulfate spectral diffusion through the formation of SSIPs, which not only organizes the sulfate hydration shell, but also bridges water molecules creating long-range cooperative constraints on hydrogen-bond dynamics of water.

#### 5.3.4 External Probe Perspective

Despite the rich and diverse behavior observed in ion-centered studies, direct interrogation of ion solvation is limited to systems having suitable intrinsic vibrational mode/s. Therefore, the addition of external reporters with active vibrational mode/s has become a powerful complementary strategy for probing molecular environments that are otherwise spectroscopically inaccessible. Vibrational reporters fall into two classes: ionic and molecular. This distinction is important when considering chemically and structurally complex electrolytes, where different regions may be selectively accessed by each probe. When accounting for diverse chemistries and concentration regimes, vibrational probes have helped us to obtain a unified picture of aqueous solvation, complementing that of intrinsic vibrational modes.

Early work focused primarily on ionic probes, which become part of ionic components of the electrolyte and therefore directly report on ion–water interactions. One early demonstration of this approach was presented by Yuan et al., where the authors showed the suitability of SeCN^–^ for probing the long timescale dynamics of aqueous electrolytes.^229^ Using its nitrile stretch, which has a strong transition dipole and relatively long vibrational lifetime, the authors showed that the spectral diffusion dominated by a single picosecond timescale, matching the collective hydrogen-bond network reorganization timescales obtained from the OD stretch of HOD in D_2_O . The authors also noted that SeCN^–^ primarily sensed the bulk-like water dynamics, rather than the slowed hydration structures commonly observed near ions. However, a subsequent study by Yamada et al. resolved an additional subpicosecond FFCF component in this anion, associated with local hydrogen-bond fluctuations (Figure 15a).^230^ Thus, these studies established that selenocyanate reports both local dynamics and collective water motions.

**Figure 15:**
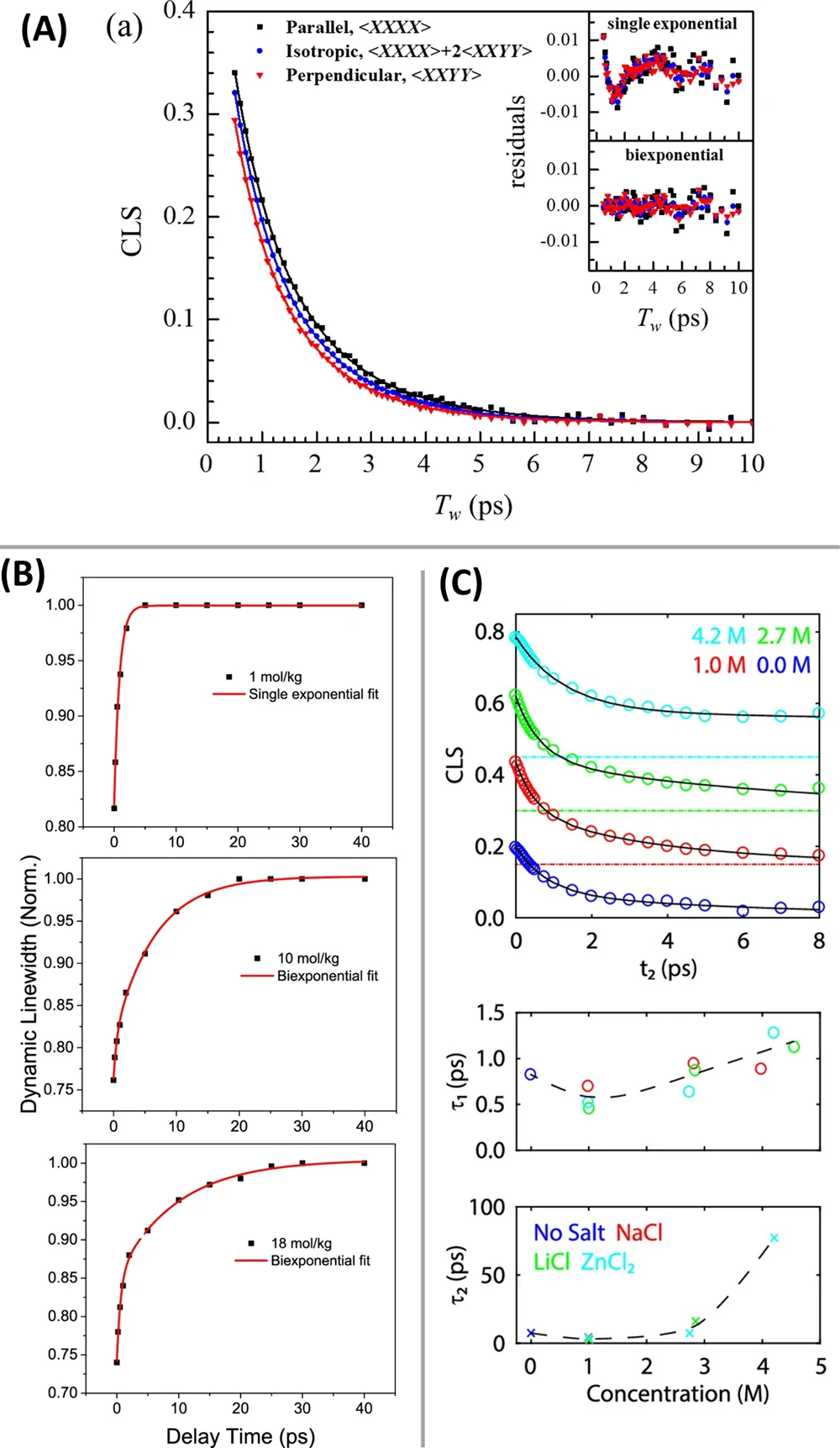
Probe dynamics in aqueous solutions of salt. Top panel: SeCN^–^ in D_2_O (A) Parallel, isotropic, and perpendicular spectral diffusion decays for the CN stretch of SeCN^–^ in D_2_O. The solid curves are biexponential fits to the data. The top and bottom insets display the residuals obtained with single and biexponential fits to the data, respectively. Reprinted from Yamada, S. A.; Thompson, W. H.; Fayer, M. D. *Water–anion Hydrogen Bonding Dynamics: Ultrafast IR Experiments and Simulations*. *Journal of Chemical Physics* **2017**, 146 (23), 234501. 10.1063/1.4984766, with the permission of AIP Publishing. Bottom left panel: SCN^–^ in the LiTFSI aqueous solutions. (B) Dynamic line width analysis of the 2DIR spectra CN stretch of SCN^–^ in the aqueous LiTFSI solution at concentrations of 1, 10, and 18 mol/kg. The solid lines are the fitting results using single or biexponential decay functions. Adapted with permission from ref.^140^ Copyright 2020 American Chemical Society. Bottom right panel: Acetone in aqueous salt solutions. (C) Centerline slope decay for acetone in ZnCl_2_ aqueous solutions with concentrations increasing from 0 to ∼ 5 M. Bottom plots contain the time scales for the decays extracted from the CLS fits. Adapted with permission from ref.^175^ Copyright 2020 American Chemical Society.

The use of ionic probes extended beyond dilute electrolytes and into highly concentrated regimes where intrinsic probes are often unavailable. Thiocyanate (SCN^–^), through its nitrile stretch, has also been used as an external vibrational reporter in highly concentrated (HCE) or water-in-salt electrolytes (WISE), where the dominant salt, LiTFSI, lacks a sufficiently sensitive vibrational transition (Figure 15b).^140^ Zhang et al. showed that at relatively low LiTFSI concentrations (∼1M), the spectral diffusion dynamics of SCN^–^ is well described by a single exponential decay with a time constant comparable to that observed in pure water. However, at high LiTFSI concentrations (10 and 18 M), an additional subnanosecond component emerges. This slow component was attributed to confinement effects imposed by a percolating TFSI^–^ network that appears at high salt concentrations and restricts solvent reorganizations. Interestingly, the authors also observed that the rotational dynamics of SCN^–^ was decoupled from the bulk viscosity, demonstrating that the probe reports the local nanodomain dynamics rather than on the dynamics of the viscous electrolyte, providing evidence for the nanoscale heterogeneity of LiTFSI WISE electrolytes.

Beyond monovalent systems, ionic probes have also proven valuable for resolving solvation structures in electrolytes containing multivalent ions. Hung et al. used KSeCN as a vibrational reporter to study aqueous solutions of MgCl_2_ in a wide range of concentrations.^238^ The study revealed two spectroscopically distinct SeCN^–^ populations: one associated with bulk water, and the other coordinated to Mg^2+^, with no evidence of their chemical exchange on the picosecond timescale. While the water-associated population exhibited the expected biexponential dynamics of spectral diffusion, characteristic of hydrogen-bond fluctuations, the Mg^2+^-bound population of the anion displayed an additional slow decay component of tens of picoseconds. This additional timescale was attributed to the rigid Mg^2+^ hydration shell, which remains constant across concentrations until near saturation. At saturation, major changes in line widths and dynamics appear highlighting the hydration shell restructuring due to water scarcity. Overall, these ionic-probe studies revealed that local water dynamics remains largely bulk-like at low concentrations, but becomes increasingly heterogeneous and spatially confined in highly concentrated electrolytes.

Neutral molecular probes offer a fundamentally different, solvent-centered viewpoint. Moreover, molecules, such as acetone and methyl thiocyanate (CH_3_SCN), make posssible to probe solvation dynamics on longer timescales than those accessible through water OD stretch alone. For example, Lewis et al. investigated the vibrational solvatochromism and dynamics of dilute acetone in aqueous NaCl, LiCl, and ZnCl_2_ solutions (Figure 15c).^175^ The carbonyl stretch of acetone exhibited both frequency shifts and solvation dynamics that scaled with cation charge density. Interestingly, 2DIR measurements revealed a nonmonotonic concentration dependence: at moderate salt concentrations, structural fluctuations were faster than in pure water, while at concentrations above ∼ 1 M, they became dramatically slower, coinciding with the electrolyte becoming more viscous. Comparison with MD simulations showed that these experimental observations could not be fully explained by electrostatic effects and dynamics alone. Instead, the results suggest that nonelectrostatic interactions also play an important role, contributing to both the observed vibrational frequencies and ultrafast solvation dynamics of the probe.

Among molecular probes, CH_3_SCN has become the reporter of choice to investigate aqueous electrolyte dynamics because it is sensitive to ion coordination, but also the probe exhibits dynamics similar to water. In early work, Yuan et al. studied CH_3_SCN in highly concentrated LiCl solutions. The authors observed spectral diffusion times comparable to those obtained from OD stretch measurements, validating the capacity of CH_3_SCN to report on the bulk dynamics of the system.^139,174^ They also detected the presence of chemical exchange between water-hydrogen-bonded (CH_3_SCN-H_2_O) and Li^+^-coordinated (CH_3_SCN-Li^+^) on tens of picoseconds timescales, with only weak dependence on viscosity or ionic strength. These results were interpreted as the exchange being governed by local coordination, rather than bulk diffusion (Figure 16a).^139^ A follow-up study by the same authors disentangled spectral diffusion and chemical exchange contributions, revealing that, in concentrated LiCl solutions, CH_3_SCN exhibits three distinct dynamical components: subpicosecond hydrogen-bond length fluctuations, few-picosecond hydrogen-bond network rearrangements, and tens of picoseconds ion pair or ion-cluster reorganizations (Figure 16b).^141^ More recently, Felsted et al. used CH_3_SCN to investigate concentrated lithium salt solutions with different anions, namely LiCl, LiBr, LiNO_2_, and LiNO_3_.^176^ While the fast subpicosecond spectral diffusion component remained largely invariant across different salts, LiNO_2_ solutions exhibited slower dynamics for both water- and lithium-coordinated CH_3_SCN species. The authors attributed this behavior to enhanced water–ion network formation, promoted by nitrite forming ion pairs, leading to more structured water environments compared to halide- or nitrate-based electrolytes.

**Figure 16:**
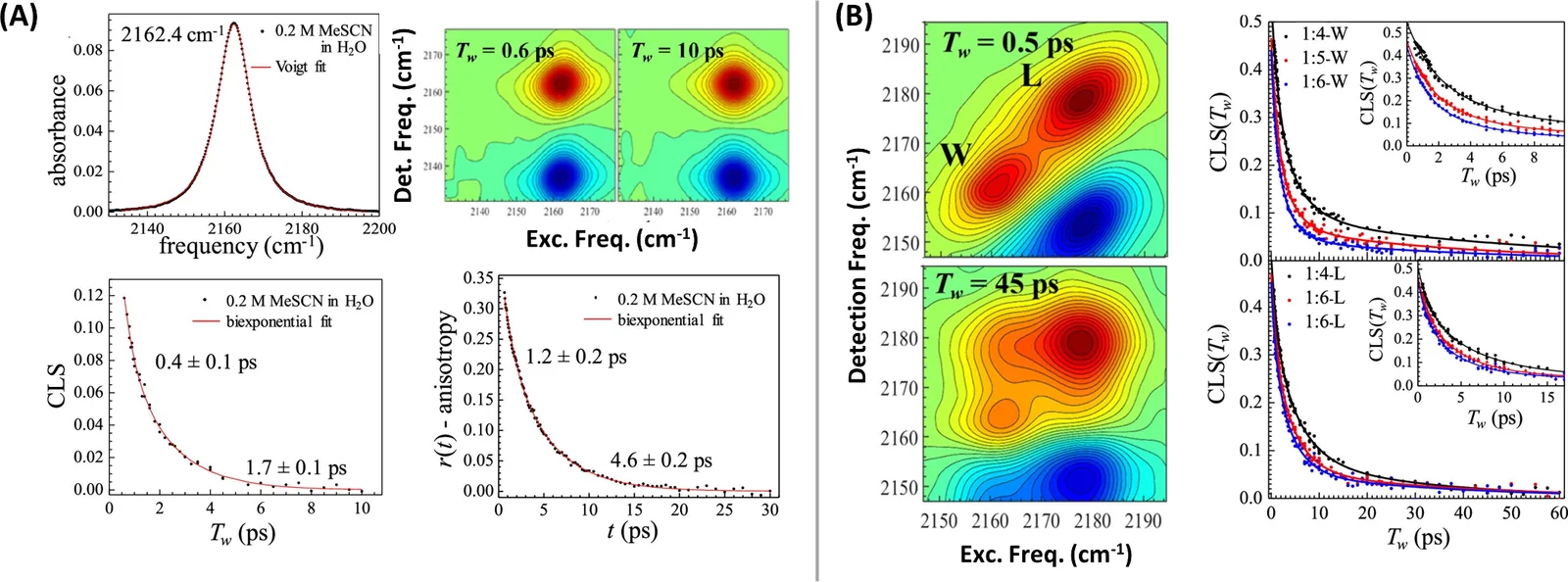
Left panel: (A) CH_3_SCN as probe of water structure. Linear IR absorption spectrum of 0.2 M CH_3_SCN in H_2_O (black dots) and its Voigt fit (red line). Examples of 2DIR spectra of this system at two waiting times (Tw). The CLS extracted from the 2DIR experiments (black dots) and its biexponential fit (red line). Finally, the anisotropy (black dots) at the peak center extracted from pump–probe experiments and its biexponential fit (red line). Adapted with permission from ref.^139^ Copyright 2018 American Chemical Society. Right panel: (B) CH_3_SCN as probe of electrolyte water structure. Experimental 2DIR spectra of CH_3_SCN in LiCl 1:4 H_2_O solution at two T_w_s. CLS as a function of waiting time for the water-bound (W) species and lithium-bound (L) species bands, respectively at three LiCl concentrations. The solid curves are triexponential fits to the data. The insets show the short time portions. Adapted with permission from ref.^141^ Copyright 2019 American Chemical Society.

Despite their proven effectiveness, external solutes can induce subtle perturbations to the system, and their measured dynamical observables are inherently influenced by the chemical nature of the probe itself. This hypothesis was tested in a comparative study by Okuda et al., who examined the vibrational dynamics of SCN^–^ and its neutral analog in 2-nitro-5-thiocyanate benzoic acid.^207^ While both probes displayed similar picosecond spectral diffusion dynamics, significant differences were observed in the extent of inhomogeneous broadening perceived by each probe. The large inhomogeneity sensed by the ionic probe was attributed to its exposure to a wider range of solvation environments. In contrast, the neutral probe lacked a long-lived FFCF component, indicating that its aromatic core does not induce a significant dynamical perturbation of the hydration shell. Nevertheless, both probes show comparable short-range hydrogen-bond rearrangements, even when their hydration structures differ, showcasing the universality of the local hydration dynamics mechanism.

#### 5.3.5 Effect of Confinement and Interfaces

In bulk electrolytes, structure and dynamics are governed by interactions among ions and solvent molecules. Under confinement, however, ion solvation, solvent dynamics, and transport mechanisms are fundamentally altered because molecular rearrangements are restricted and ion-interface interactions become more pronounced. Across a range of confined environments, including nanoporous polymers, mesoporous solids, micelles, and electrochemical interfaces, 2DIR studies have revealed that confinement induces persistent dynamical heterogeneities and restructuring of electrolyte free-energy landscapes.

Early molecular insights into confinement-modified solvent dynamics in electrolytes were provided by Kuroda et al.^147^ Using very low concentrations of the TCM^–^ in water as a vibrational reporter in reverse micelles, this work demonstrated that ions in nanoconfined water solutions cannot fully randomize their hydrogen-bond solvation shell as fast as in bulk. 2DIR spectra revealed long-lived frequency correlations and suppressed spectral diffusion, confirming the core–shell picture, in which interfacial solvent dynamics are kinetically distinct from interior water (Figure 17a). Moreover, the authors developed a model to describe the heterogeneity of water dynamics, predicting that this heterogeneity disappears within a single water layer from the interface. This study provided the first semiquantitative framework for interpreting confinement effects in electrolytes.

**Figure 17:**
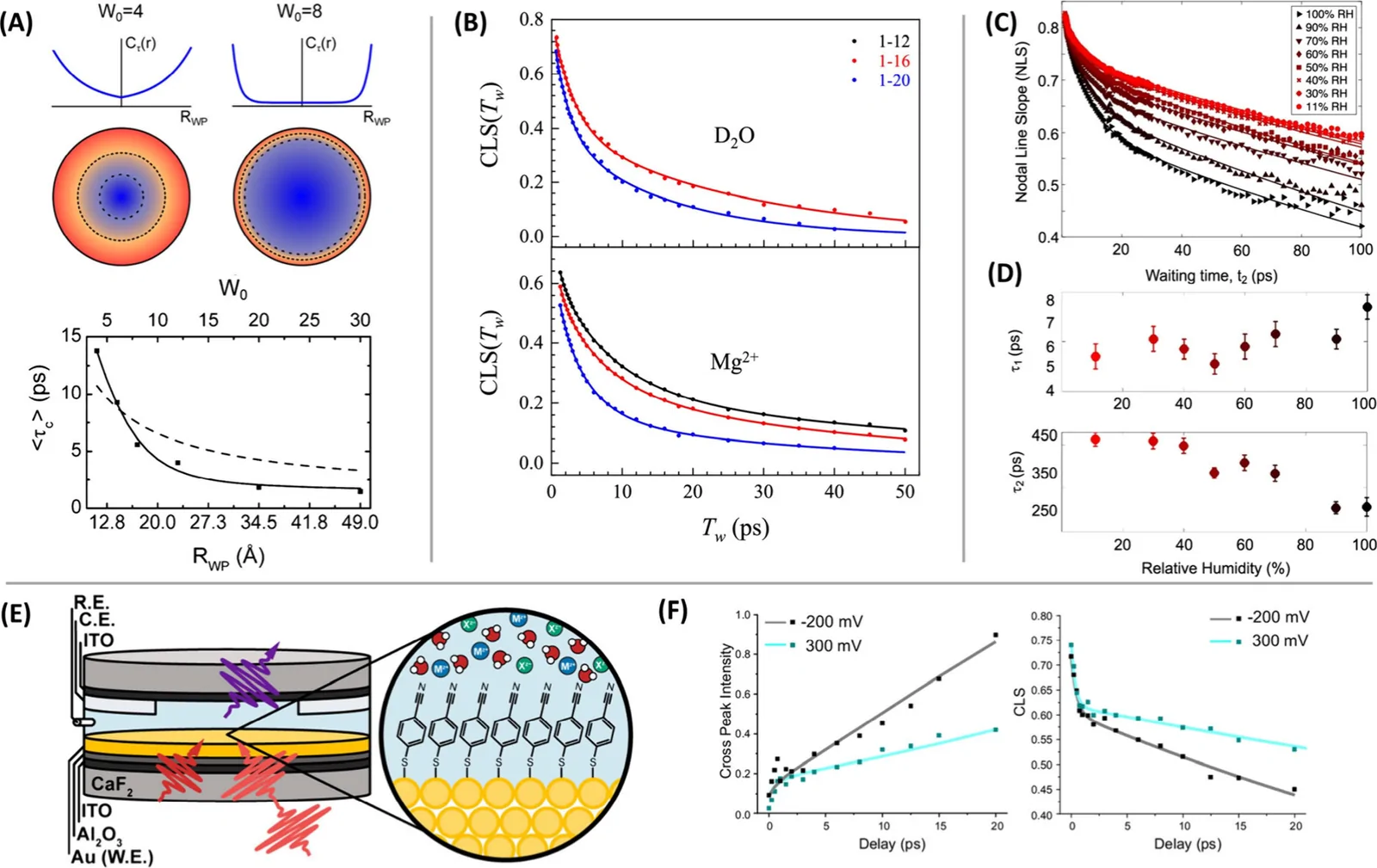
Dynamics of aqueous electrolytes in confinement. Top left panel: Heterogeneous hydration dynamics perceived by an ion a reverse micelle. (A) Schematic presentation for the variation of the FFCF correlation time of TCM^–^ as a function of distance from the interface for small (W_0_ = 4, left) and large (W_0_ = 8, right) micelles. Bottom panel of A) showcases the variation of the decay of the FFCF correlation time as a function of the micelle size and the fitting with the heterogeneous model. Adapted with permission from ref.^147^ Copyright 2013 American Chemical Society. Top center panel: (B) CLS decays of SeCN^–^ in aqueous MgCl_2_ solutions of varying concentrations confined in 2.8 nm silica pores. High and low panels are the data for the D_2_O-associated peak and for the Mg^2+^-associated peak, respectively. Adapted with permission from ref.^145^ Copyright 2024 American Chemical Society. Top right panel: (C) Nodal line slope as a function of waiting time at different humidity values (RH) along with the biexponential fitting in solid lines. (D) Short (τ_1_) and long time (τ_2_) constant obtained from spectral diffusion analysis as a function of RH representing the hydrogen-bond dynamics. Adapted with permission from ref.^210^ Available under a CC-BY-NC-ND license. Copyright 2026 Ranadeb Ball et al. Bottom panel: (E) Schematic of 2DIR Spectroelectrochemical cell and inset is a schematic of the surface and representative electrical dual layer. Adapted with permission from ref.^177^ Copyright 2025 American Chemical Society. (F) Cross peak intensity and CLS extracted from the 2DIR spectra where the dots represent experimental data whereas the lines represent their modeling. Adapted with permission from ref.^265^ Available under a CC BY-NC-ND license. Copyright 2023 Ryan et al.

The influence of confinement on electrolytes at higher salt concentrations was later quantified by Hung et al.^145^ The authors used SeCN^–^ as a vibrational probe to investigate the dynamics of water and concentrated MgCl_2_ solutions confined within mesoporous silica (Figure 17b). Although the linear spectra of the confined solutions do not exhibit distinct shell and core distributions, the 2DIR spectroscopic characterization showed that the confinement increased the variety of probe-solvent interactions, and it amplified the ion–ion correlations at high salt concentrations. In particular, the MgCl_2_ aqueous solutions under confinement exhibited increased inhomogeneous broadening and slower spectral diffusion dynamics for both the water-associated SCN^–^ and the Mg^2+^-associated SCN^–^. After spatially decomposing the averaged pore dynamics into shell and core components, the authors found that the Mg^2+^-associated shell dynamics are impacted more by confinement, consistent with a more restricted rearrangement and stabilization of ion clusters. These results highlighted that confinement and salt concentration act synergistically, driving electrolyte behavior away from bulk-like solvation toward ionic network dominated dynamics, as seen in high concentrated electrolytes.

Electrolyte confinement has also been directly linked to changes in ion transport behavior. Ball et al. studied a confined electrolyte in polymeric anion exchange membranes (AEMs).^210^ Combining 2DIR spectroscopy with macroscopic conductivity measurements and MD simulations, the authors demonstrated that ion diffusion is primarily dictated by subdiffusive ion hopping timescales within sub-2 nm charged pores, leading to reduced ionic mobility (Figure 17c,d). The results were interpreted as confinement immobilizing parts of the solvation shell, thereby increasing the energetic cost of ion hopping between sites. More importantly, the authors established that hydration primarily regulates transport by tuning ion pair interactions, which in turn influences the enthalpic activation barrier for ion hopping in extremely nanoconfined anion exchange membranes. Therefore, the authors concluded that tuning the ion–ion interaction to lower the activation barrier for ion hopping could serve as a design principle for next generation AEMs.

Studies on ion specificity and selectivity under confinement were further pursued by Johnson et al.^209^ Using 2DIR spectroscopy, the authors probed the hydration dynamics of anions within uniformly charged nanoporous polymers having pore diameters of ∼ 1 nm. The authors observed that weakly hydrated anions (SCN^–^) experience stronger confinement-induced heterogeneity than strongly hydrated counterparts (N_3_^–^), as manifested by the suppressed chemical exchange and long-lived frequency offsets associated with surface-bound solvation environments. The study also demonstrated that confinement magnifies the hydration differences between ions having constrasting interactions with water, directly influencing selectivity and transport efficiency.

Confinement effects at soft interfaces were also probed by Roy et al.^208^ This work revealed that ions localized near micellar head groups experience dramatically slowed hydration dynamics compared to bulk solution. The authors observed incomplete spectral diffusion dynamics even at long waiting times, indicating that ions are effectively trapped in interfacial solvation environments. These findings showed that confinement does not necessarily require rigid pores since soft interfaces can impose comparable constraints on the electrolyte dynamics.

At electrochemical interfaces, the solvent dynamics can additionally be modulated by applied electric fields. Ryan et al. used the newly developed surface-enhanced (SE) 2DIR spectroscopy and benzonitrile-functionalized surfaces to demonstrate that electrode polarization alters interfacial water structure and electrolyte dynamics (Figure 17e,f).^265^ The authors observed that an applied voltage produces non-negligible changes in the spectral diffusion and vibrational lifetimes, which is consistent with field-induced reorientation of interfacial hydrogen-bonded water networks. This work demonstrated that the electrolyte interface can dynamically tune solvation environments via electrostatic potentials. Gilbert et al. extended these ideas in a follow-up work.^177^ The authors used benzonitrile-functionalized electrode surfaces and SE-2DIR spectroscopy to investigate the effect of voltage and an electrolyte composition on the dynamics of electric double layers. The authors found that the surface dynamics strongly depends on voltage polarity and electrolyte composition, which was explained by the impact of the chemical identity of the ions through direct ion-dipole interactions and indirectly through changes in the solvent properties. Their results also revealed that different anions impose distinct interfacial confinement regimes, leading to electrolyte-dependent energy dissipation and solvation dynamics in the presence of a potential. Together, these two studies using SE-2DIR spectroscopy demonstrated that electrochemical interfaces are both structurally and electrically programmable.

All these 2DIR studies revealed how confinement and interfaces modulate the microscopic processes, such as solvation-shell fluctuations, dynamical heterogeneity, and chemical exchange, which define ion transport and electrochemical phenomena in aqueous electrolytes.

### 5.4 Solvation Shell Dynamics of Organic Electrolytes

Over the past two decades, 2DIR spectroscopy has also provided detailed molecular-level insights into organic solvent electrolytes. These studies provided a much-needed characterization of electrolyte structure, ion–solvent interactions, solvation shell dynamics, and elementary transport mechanisms.

#### 5.4.1 Organic Solvent Perspective

The solvation shell of metal cations has become one of the most extensively studied aspects of organic electrolytes. In contrast to their aqueous analogues, the vibrational modes of common organic solvents are highly sensitive to the presence of metal cations, such as Li^+^. This sensitivity arises from the solvents coordinating to the cation through electron-rich functional groups, which usually have strong and sensitive vibrational signatures. Consequently, free and cation-bound solvent populations are often spectroscopically distinguishable, allowing direct monitoring of their structure and dynamics.

Early work by Yuan et al. established foundational insights into Li^+^ solvation in acetonitrile (CH_3_CN).^197^ Using femtosecond anisotropy measurements, the authors inferred a Li^+^ coordination number of four to six and demonstrated that solvent residence times exceed tens of picoseconds. Results from 2DIR spectroscopy also allowed the determination of a lower bound estimate for the Li^+^-CH_3_CN complex lifetime, since no chemical-exchange cross peaks were detected in the 20 ps experimental window. This observation provided strong evidence of Li^+^-solvent being a long-lived interaction (Figure 18a),^197^ which was later confirmed via ab initio MD simulations.^137^

**Figure 18:**
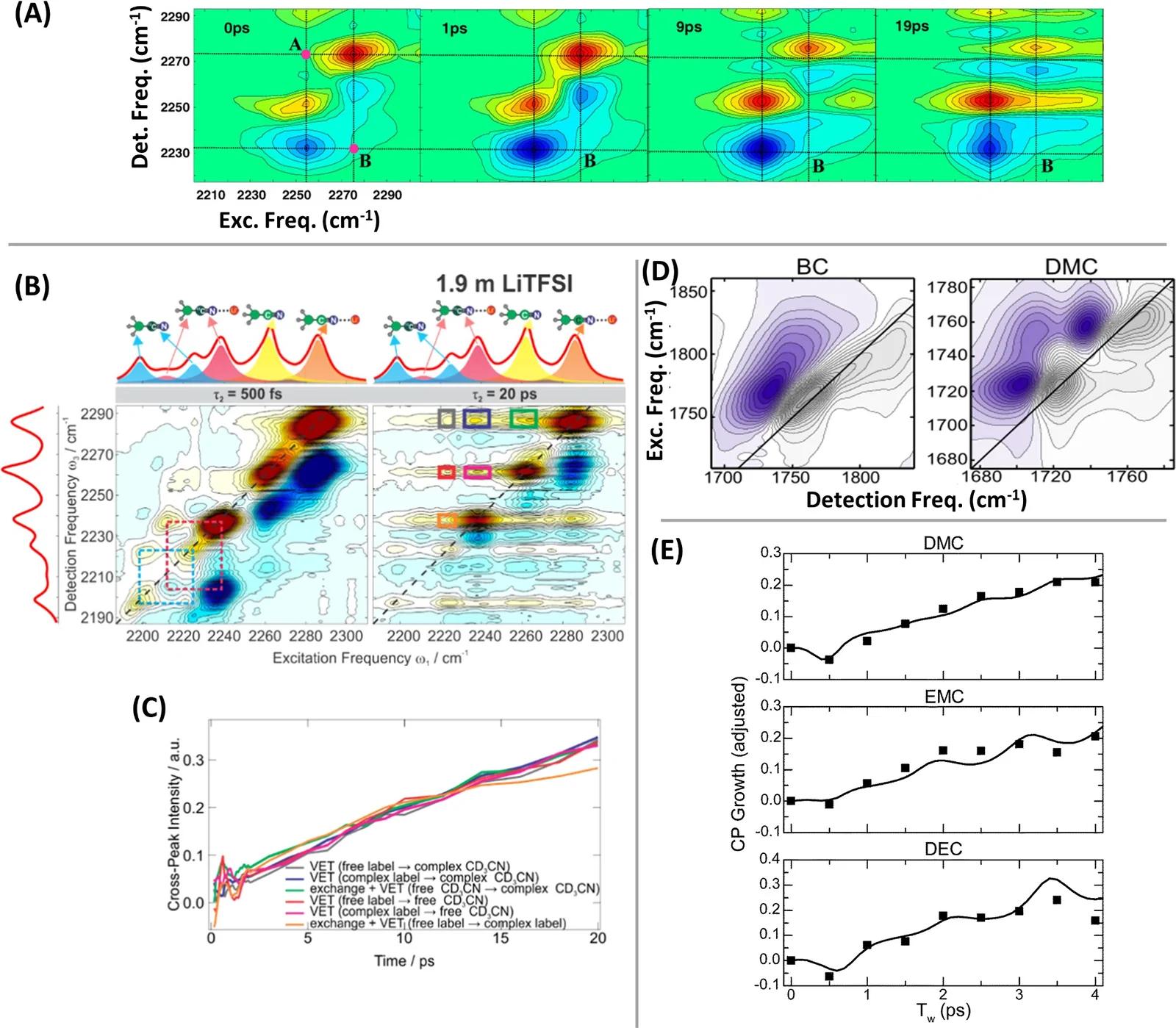
Solvation of cations in organic solvents. Top panel: (A) Waiting time evolution of 2DIR spectra of LiBF_4_ in CH_3_CN at 1 mol/kg. Adapted with permission from ref.^197^ Copyright 2014 American Chemical Society. Left panel: Separation of the chemical exchange and energy-transfer pathways in LiTFSI CH_3_CN solutions. (B) Linear IR spectra (red line, top and left) with the decomposition into contributions from different species (cyan: free label; pink: complexed label; yellow: free CH_3_CN; orange: complexed CH_3_CN; purple: water-mediated metal complex). 2DIR spectroscopy of 1.9 m LiTFSI in a 1:1 mixture of the label and CD_3_CN At a waiting time of 500 fs and 20 ps. Dashed rectangles denote the Fermi-like coupling between the C–D and C≡N modes of the free (cyan) and complexed (pink) labels. Colored solid rectangles at late waiting time designate integration regions to track the kinetics of various pathways. (C) Kinetics of the integrated peaks shown in (B). Adapted with permission from ref.^190^ Copyright 2022 American Chemical Society. Middle right panel: Li^+^ in organic carbonates. (D) 2DIR spectra of LiPF_6_ in BC (left) and DMC (right) at X­(Li+)=0.09 for waiting time *T* = 0 ps with < XXYY> polarization. Adapted with permission from ref.^85^ Copyright 2016 American Chemical Society. Bottom right panel: (E) Cross peak growth as a function of T the electrolytes containing dimethyl carbonate (DMC), ethylmethyl carbonate (EMC) and diethylcarbonate (DEC). Solid black lines represent the model of the data. Used with permission of The Royal Society of Chemistry, from *A Comparison of the Solvation Structure and Dynamics of the Lithium Ion in Linear Organic Carbonates with Different Alkyl Chain Lengths*, Kristen D. Fulfer and Daniel G. Kuroda, *Physical Chemistry Chemical Physics*, Vol. 19, Issue 36, 2017; permission conveyed through Copyright Clearance Center, Inc.^266^

Following this work, Fulfer et al. performed the 2DIR investigation of Li^+^ solvation in carbonate-based electrolytes.^85^ The authors observed cross peaks at zero waiting-time and attributed them to vibrational coupling among carbonate molecules in the first solvation shell (Figure 18d). Modeling supported tetrahedral coordination motifs involving both linear and cyclic carbonates. Spectral diffusion further showed that the cyclic carbonate exhibits a more internally ordered solvent structure than the linear carbonate, an effect attributed to antiparallel dipolar ordering in cyclic carbonates, even in the absence of salt. A later study related this aspect to cyclic solvent molecules of the second solvation shell intercalating into the first one.^267^

Subsequent work by the same group demonstrated that subtle variations in the molecular structure of the linear carbonate modulate Li^+^ solvation shell structural dynamics (Figure 18e).^266^ Analysis of the 2DIR cross peaks showed that increasing alkyl-chain length in linear carbonates distorted the tetrahedral Li^+^ coordination geometry and slowed solvation shell deformations. In addition, oscillatory growth of cross peaks provided strong evidence for excitonic coupling among carbonyl modes in the solvation shell. The results of this study were later corroborated by ab initio MD simulations.^267^ A complementary dilution study by Fulfer et al. consisting on replacing linear carbonates with tetrahydrofuran,^196^ showed a decrease in cross peak intensity and a slowdown in the anisotropy dynamics due to a weaker vibrational coupling in the dilute sample, agreeing with the interpretation reached before by the same authors.

During the same time, Lee et al. investigated carbonate electrolytes using 2DIR experiments alongside MD simulations by Liang et al.^135,268^ While their spectra also showed time-dependent cross peak intensities, these features were initially attributed to rapid chemical exchange between free and bound carbonates. This interpretation was later revised by Lim et al.,^198^ who demonstrated that the observed cross peaks arise from interchromophore energy transfer between coordinated and free solvent molecules, with growth times of ∼ 10 ps. Moreover, the authors also showed that all these cross peaks were insensitive to salt concentration or solvent composition.

Analogous behavior was later observed in other Li^+^-binding solvents. Chen et al. reported similar tetrahedral solvation shells in urea-based electrolytes, with 2DIR cross peaks arising from vibrational coupling.^263^ Notably, cyclic and linear ureas exhibited opposite trends from carbonates, with cyclic ureas producing more distorted solvation environments.

Unlike organic carbonates and ureas, the nitrile stretch of CH_3_CN has very weak vibrational coupling due to its small transition dipole moment, and therefore does not display vibrational coupling cross peaks in the 2DIR spectra. Instead, a strong Fermi resonance, arising from coupling between the nitrile stretch and a combination band of CH_3_CN. Kwon et al. showed that the effect of Fermi resonance becomes more pronounced upon Li^+^ coordination, thereby complicating the characterization of the Li^+^ solvation shell.^194^ To circumvent this issue, several groups have used different isotopologues to study the structure and dynamics of CH_3_CN electrolytes more effectively.

Chen et al. examined Li^+^ solvation in CD_3_CN and found remarkably similar spectral-diffusion dynamics for free ions and contact ion pairs.^137^ These results were attributed to angular “in-cone” fluctuations of CD_3_CN molecules, with spectral diffusion governed primarily by Li^+^-N≡C angular motions rather than changes in Li^+^-solvent distance or anion identity. Later, Dereka et al. systematically evaluated different CH_3_CN isotopologues in concentrated LiTFSI electrolytes to disentangle the contributions from chemical exchange and vibrational coupling.^189^ CD_3_CN emerged as a particularly effective probe due to its narrow spectral features and absence of Fermi resonances, in agreement with the previous work by Chen et al.^137^ Finally, a follow-up study by Dereka et al. combined CD_3_CN and CD_3_^13^CN to investigate the different vibrational energy transfer pathways. The authors demonstrated that 2DIR cross peaks in concentrated electrolytes arise exclusively from intermolecular vibrational energy transfer, establishing that ion–solvent bond breaking does not occur on sub hundred picosecond timescales (Figure 18b,c).^190^

The analysis of cross peak growth was further extended to high-concentration electrolytes (HCEs), composed of LiTFSI and carbonyl-containing solvents. Galle Kankanamge et al. reported picosecond-scale cross peak growth attributed to solvent exchange, which was initiated by conformational fluctuations of nearby TFSI^–^ anions.^136^ These rearrangements were proposed to preserve Li^+^ preferred coordination number of 4, while inducing solvent molecules to de- and recoordinate to other lithium centers. The authors also observed spectral features consistent with solvent dimer formation in butylene carbonate (BC), potentially contributing to the high viscosity of cyclic carbonates. A complementary study by Chen et al. demonstrated that increasing anion size in sulfonylimide-based in CH_3_CN-based HCEs systematically slows solvent dynamics.^192^ The authors also identified a correlation between picosecond solvation dynamics and macroscopic viscosity and resistivity of the electrolyte, demonstrating that elementary ion–ion rearrangements sometimes relate to both viscosity and ionic transport.

The collective knowledge of pure HCE gave rise to investigations of more complex systems. Wu et al. showed that adding a fluorinated antisolvent to an HCE composed of LiFSI/DMC electrolytes increases the slowest FFCF component associated with the local solvent rearrangements around Li^+^.^193^ This effect was attributed to a reduced ion density and a constrained network reorganization, consistent with prior observations by Rushing et al.^269^ The authors also noted that, despite the structural slowdown, vibrational energy transfer between free and coordinated DMC accelerated, indicating that TTE simultaneously restricts reorganization while enhancing Li^+^ diffusion pathways. MD simulations also supported this dual role, demonstrating that the behavior of complex solutions cannot be derived solely from the knowledge gained from binary systems.

#### 5.4.2 Anion Perspective

Early investigations of anion solvation in organic solvents revealed striking parallels with aqueous systems. Ohta et al. studied OCN^–^ and SCN^–^ in methanol using three-pulse photon echoes and identified both ultrafast (subpicosecond) and slower (picosecond) spectral diffusion components.^228^ The nearly identical dynamics of these chemically distinct ions demonstrated that solvent motions dominate frequency fluctuations, while ion–solvent interaction strength primarily determines the FFCF fluctuation amplitude. A subsequent study by the same group extended this framework to other protic and aprotic organic solvents (Figure 19a).^237^ Using SCN^–^ and N_3_^–^, the authors showed that biexponential spectral diffusion persists irrespective of the solvent, but with distinct spectral diffusion dynamics. In aprotic solvents, fast and motionally narrowed components dominate, whereas in protic solvents the slow component and static offset substantially increase. The observed behavior showcased the important role of hydrogen-bonding in the solvation dynamics of anions.

**Figure 19:**
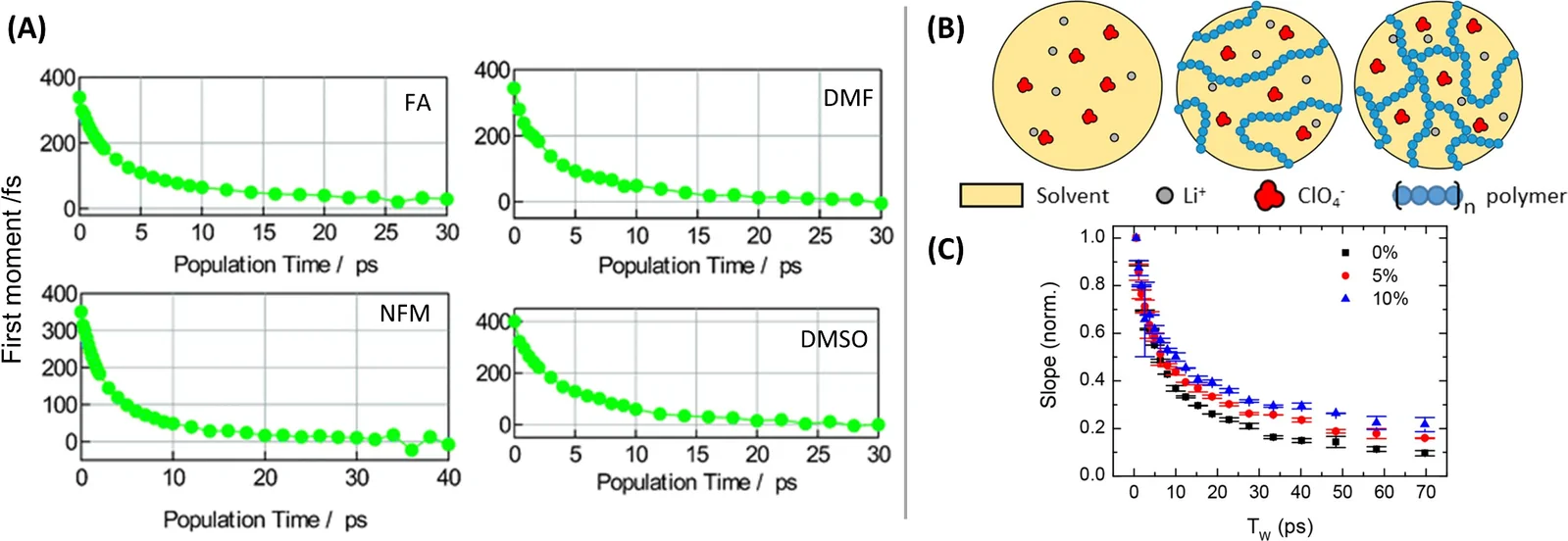
Anions solvation in organic solvents. Left panel: Anion solvation in simple solvents. (A) First moment of the photon echo signals for SCN^–^ in formamide, dimethylformamide, *N*-Methylformamide, and DMSO. Used with permission of The Royal Society of Chemistry, from Ultrafast vibrational dynamics of SCN^–^ and N3^–^ in polar solvents studied by nonlinear infrared spectroscopy, Kaoru Ohta, Jumpei Tayama, and Keisuke Tominaga, Physical Chemistry Chemical Physics, Vol. 14, Issue 30, 2012; permission conveyed through Copyright Clearance Center, Inc.^237^ Right panel: Anion solvation in organic gels. (B) Cartoon representation of the electrolyte molecular structure with polyacrylonitrile (PAN) concentration increasing from 0% to 5% to 10% by mass. (C) Slope as a function of waiting time for the electrolytes containing 0% PAN (black), 5% PAN (red), and 10% PAN (blue). Reprinted from J. C. Rushing, A. Gurung, and D. G. Kuroda, “Relation between microscopic structure and macroscopic properties in polyacrylonitrile-based lithium-ion polymer gel electrolytes,” *The Journal of Chemical Physics* **158**, 144705 (2023), 10.1063/5.0135631, with the permission of AIP Publishing.^182^

The influence of the organic solvent environment on symmetry and solvation dynamics was further highlighted in work by Yang et al. on hexacyanocobaltate (Co­[CN]_6_^3–^) in protic solvents.^103^ The authors showed that chemical exchange and solvent-induced symmetry breaking coexist, but occur on timescales that are solvent dependent. For example, in highly viscous alcohols, such as glycerol, symmetry breaking was nearly an order of magnitude slower than in ethylene glycol.

More recent efforts have focused on resolving the microscopic solvation structure surrounding anions in so-called “good” organic electrolyte solvents. Kore et al. used 2DIR spectroscopy to study thiocyanate in propylene carbonate, identifying the same dual components of the FFCF dynamics: a fast (picosecond) solvation shell fluctuations, and a slower (tens of picoseconds) collective solvent reorganization.^236^ Also, the authors observed a close correspondence between orientational relaxation and spectral diffusion and suggested that solute reorientation is also governed by the same solvent structural rearrangements.

Building on this framework, Rushing et al. extended anion-based solvation studies into more complex carbonate-based polymer gel electrolytes (Figure 19b,c).^182^ Using SCN^–^ in LiClO_4_/EC:PC polymer gels, the authors noticed that, despite dramatic increases in viscosity with polymer concentration, both ionic conductivity and spectral-diffusion dynamics were only weakly affected. The decoupling of transport properties was interpreted as the local solvation environment of the ions remaining largely liquid-like, even as the system transitions into a highly structured gel. The authors attributed the spectral diffusion of the ions to the polymer behavior, which mainly participates in the formation of the gel matrix, while the solvent and salt form an electrolyte confined by the gel matrix.

#### 5.4.3 External Probe Perspective

The molecular components of lithium organic electrolytes often do not serve as vibrational probes of the structural dynamics, and so many studies have instead used vibrational reporters with long vibrational lifetimes to access slow timescale processes.The use of probes has also made possible to disentangle many different processes, such as chemical exchange, thermal effects, and structural rearrangements, that are otherwise difficult to resolve.

An example of the use of external probes is the study by Kwon et al., which investigated the ion–molecule complexation dynamics between LiClO_4_ and methyl thiocyanate (CH_3_SCN) in CH_3_CN.^195^ The authors monitored the Li^+^-CH_3_SCN complex and measured a timescale of hundreds of picoseconds for the chemical exchange between free and Li^+^-bound CH_3_SCN (Figure 20a,b,c). Thus, this work established that vibrational probes are effective reporters of slow dynamics processes in organic electrolytes.

**Figure 20:**
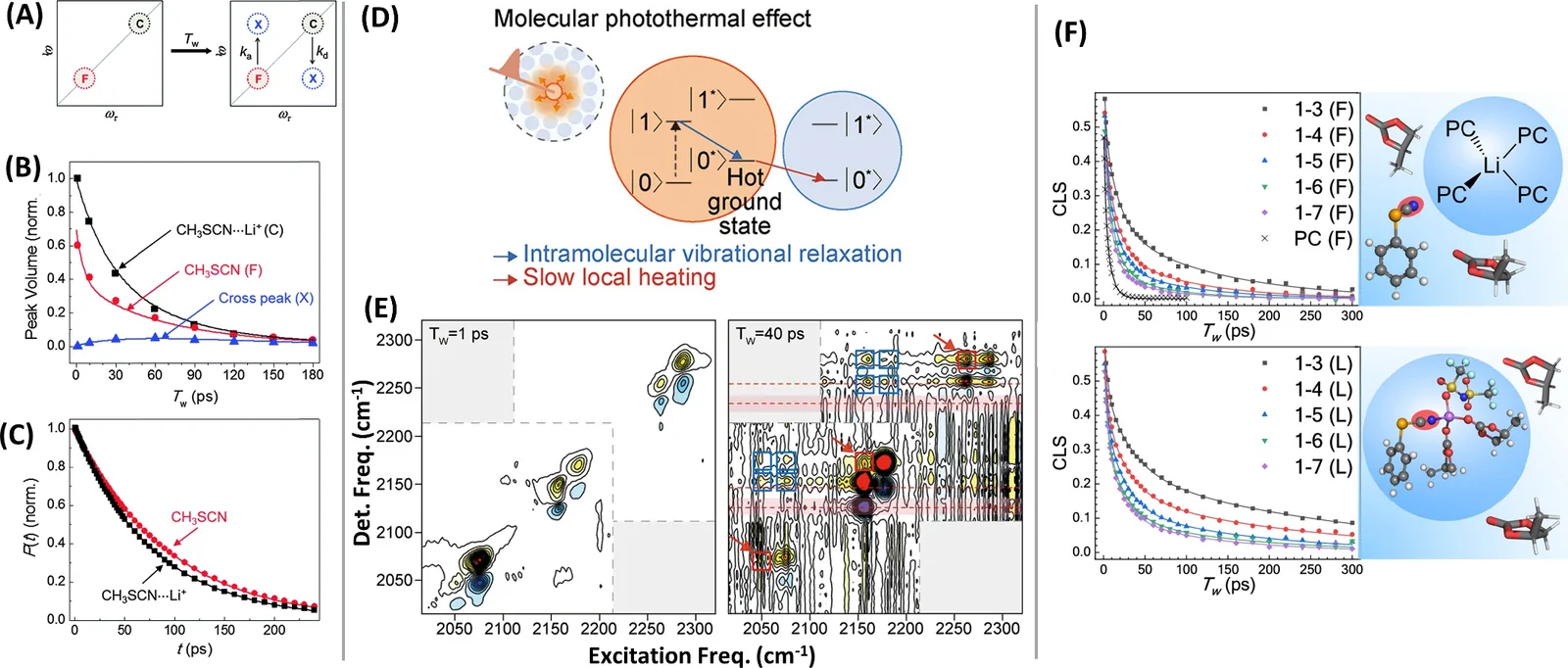
Probes in organic electrolytes. Left panel: CH_3_SCN and Li^+^ in CH_3_CN. (A) Two-species exchange kinetics observed in the T-dependent 2DIR spectra. (B) The volumes of the diagonal peaks and cross peak as a function of waiting time. (C) Vibrational lifetimes, P­(t), obtained from polarization-controlled IR PP experiments. Used with permission of The Royal Society of Chemistry, from “Complexation dynamics of CH_3_SCN and Li^+^ in acetonitrile studied by two-dimensional infrared spectroscopy,” YoungAh Kwon and Sungnam Park, *Physical Chemistry Chemical Physics*, Vol. 17, Issue 37, 2015; permission conveyed through Copyright Clearance Center, Inc.^195^ Middle panel: (D) Schematic illustration of the molecular photothermal (or local heating) effect (MPTE), generating cross peaks in 2DIR spectrum. (E) Time-resolved 2DIR spectra of 0.05 M LiSCN, 1 M LiBr, and 5 M CH_3_SCN in CD_3_CN solution at *T* = 1 and 40 ps. The blue and red squares in represent the cross peaks that can be produced by VET and VET+CE processes, respectively. Adapted with permission from ref.^179^ Copyright 2024 American Chemical Society. Right panel: Phenyl selenocyanate (PhSeCN) in propylene carbonate LiTFSI solutions. (F) Spectral diffusion dynamics for the free (F) PhSeCN and for the Li^+^-bound PhSeCN (L) in LiTFSI/PC electrolytes and pure PC (cross symbols). Lines are triexponential decay fits to the data (points). The right side contains the schematic showing the probed PhSeCN CN group. Adapted with permission from ref.^166^ Copyright 2024 American Chemical Society.

Another example is the work of Chun et al., who used time-resolved IR spectroscopy to elucidate the dominant role of the molecular photothermal effect (MPTE). To this end, the authors studied the long-time dynamics observed by CH_3_SCN in CH_3_CN-based lithium electrolytes (Figure 20d,e).^179^ The authors demonstrated that long-delay cross peaks in such systems arise primarily from MPTE-driven solute–solvent energy transfer rather than from vibrational energy transfer or chemical exchange, emphasizing the need to explicitly account for these contributions.

More recently, the use of external vibrational reporters was extended to carbonate-based electrolytes. Pan et al. introduced phenyl selenocyanate (PhSeCN) to probe the long timescale dynamics in cyclic carbonate lithium-ion electrolytes using 2DIR spectroscopy (Figure 20f).^166^ Chemical exchange between free PhSeCN and Li^+^-coordinated PhSeCN was observed on timescales of several hundred picoseconds across a wide range of salt concentrations, revealing that diffusion dominates lithium transport in these electrolytes. The authors also observed a close correspondence between the PhSeCN-Li^+^ dissociation time and the slowest component of PhSeCN-Li^+^ spectral diffusion, suggesting that partial desolvation dynamics of the Li^+^ solvation shell is the rate-limiting step in the ionic transport of these electrolytes.

The use of PhSeCN as a probe was extended to mixed-solvent systems by Charnay et al.^188^ Using both 2DIR spectroscopy and NMR characterizations on binary mixtures of carbonates, the authors revealed that vehicular diffusion increases inversely with viscosity, while structural diffusion remains largely invariant across solvent compositions. The authors also noted that this behavior is intrinsic to carbonate-based electrolytes, which predominantly rely on vehicular diffusion. Pan et al. also used the PhSeCN to probe the molecular mechanisms of lithium-ion transport in poly­(propylene carbonate) gel-polymer electrolytes.^187^ In this case, increasing polymer content slowed down the solvent exchange and simultaneously reduced Li^+^ coordination number in the electrolyte, demonstrating that ion solvation dynamics is largely decoupled from the slower polymer motions in the electrolyte. Further, the authors introduced a new molecular descriptor for ionic conductivity, consisting of the ratio of solvent residence time and the Li^+^ coordination number, which appears to capture the fundamental timescale for the partial desolvation that governs ion transport in these complex environments.

## 6 Ionic Liquids

In the last decades, ultrafast infrared spectroscopies, particularly 2DIR, have provided a molecular-level picture of the IL structure and dynamics from femtoseconds to subnanoseconds timescales. These studies have provided valuable insights into how and why chemical motifs in the IL determine its bulk properties, such as CO_2_ uptake and ion mobility, as well as how the IL structure changes in the presence of other components, such as water. However, the interpretation of the dynamic components of the ILs in the short and long timescale proved to be nontrivial. Hence, many studies required substantial conceptual advancements to interpret the time dependent data and methodological advancements in the form of specific vibrational probes to study these systems.

### 6.1 Bulk Structure, Dynamics, and Transport

Early interpretation of 2DIR experiments attributed spectral diffusion primarily to structural rearrangements of local structures in IL, such as ionic cages and hydrogen-bonding networks. Subsequent studies, however, demonstrated that solvation and consequently transport in ILs are governed by randomly oriented ion cages rather than persistent ion pairs.

The ion-cage model for ILs emerged from early 2DIR studies using small vibrational probes capable of reporting on local electrostatic and structural fluctuations. An important 2DIR work by the Garret-Roe group using a 1-butyl-3-methylimidazolium thiocyanate IL as a reporter established three foundational insights into IL dynamics.^245^ First, analysis of the FFCFs show the presence of at least two dynamical components: a fast, few-picosecond decay associated with localized “rattling” of the probe within a quasi-static ionic cage and a slower, tens of picoseconds decay attributed to the ionic cage rearrangements. This assignment was later supported by molecular dynamics (MD) simulations by Kirchner and Maginn groups, which predicted the formation of ion pairs and ion cages, with relaxation times comparable to the slower 2DIR component.^270–272^ Second, the study elucidated the important role of hydrogen-bonding in IL dynamics, demonstrating that methylation at the C2 position of the imidazolium cation weakens local hydrogen-bonding interactions, leading to longer-lived cages.^245^ Third, and most consequential for the use of ILs as electrolytes, the increase in ionic cage lifetimes was found to correlate closely with an increase in macroscopic viscosity.^245^ This result demonstrated that the fundamental step governing macroscopic transport is the making and breaking of these ionic cages.

A subsequent study by Ren et al. also demonstrated that the identity of the cation critically influences the dynamics reported by the probe (Figure 21a).^251^ When the cation matched that of the investigated IL, the authors did not observe any evidence of ion pairing or aggregation over probe at millimolar concentrations. In contrast, incorporation of SCN^–^ as KSCN led to the emergence of a new, long-lived and unresolved correlation time (>100 ps), which the authors attributed to the formation of ion pairs.

**Figure 21:**
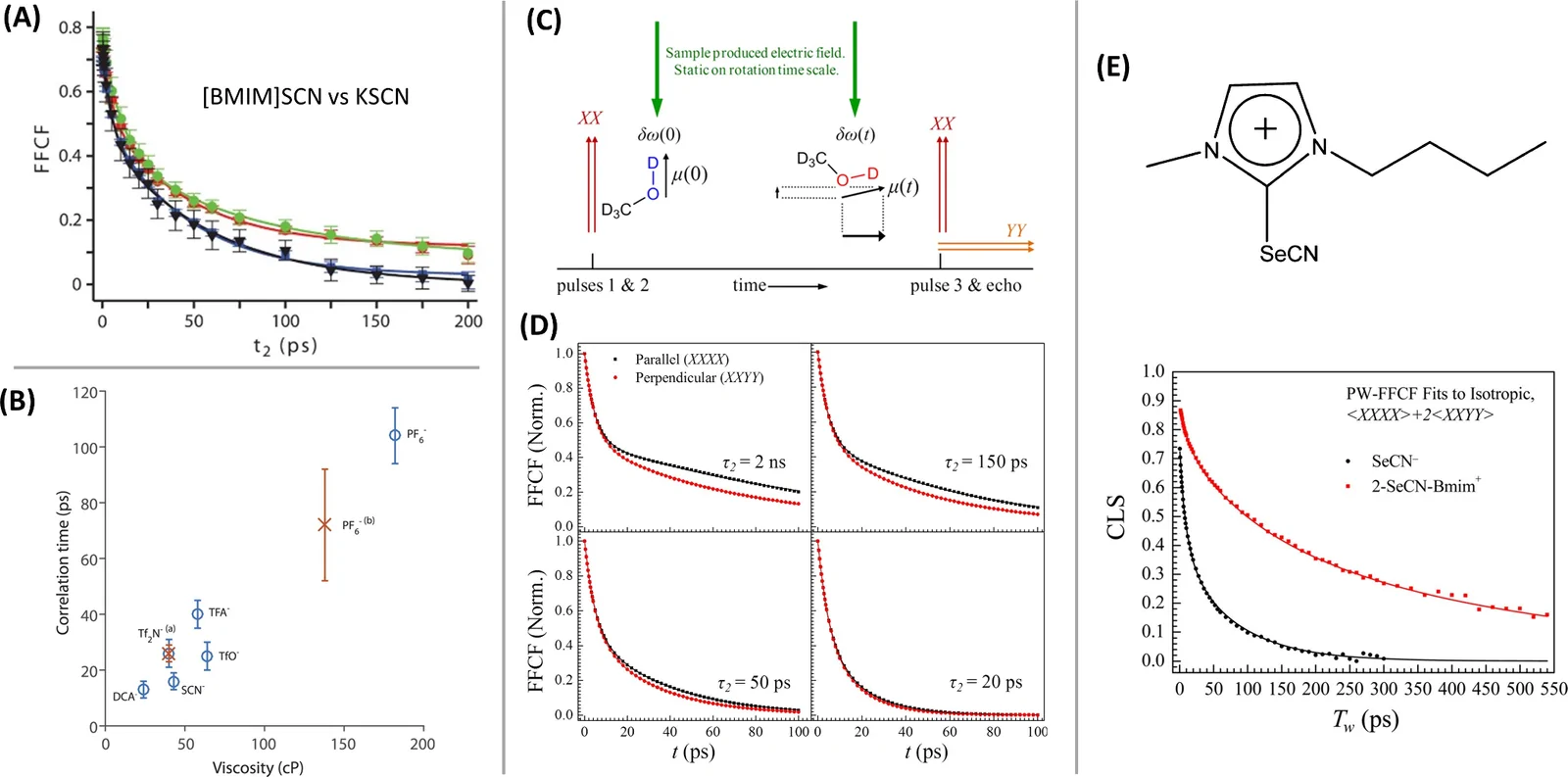
Structural dynamics of ionic liquids. Top left panel: Cation effect on dynamics. (A) FFCF as a function of waiting time shows showcasing the difference between the KSCN (red and green) and [BMIm]­[SCN] (blue and black) systems. Adapted with permission from ref.^251^ Copyright 2015 American Chemical Society. Bottom left panel: FFCF of CO_2_, SCN^–^ and HDO in different ILs. (B) Correlation time, τ_c_, compared with viscosity for the 1-butyl-3-methylimidazolium ionic liquid solvent. Reprinted from T. Brinzer et al., “Ultrafast vibrational spectroscopy (2D-IR) of CO_2_ in ionic liquids: Carbon capture from carbon dioxide’s point of view,” *The Journal of Chemical Physics* **142**, 212425 (2015), 10.1063/1.4917467, with the permission of AIP Publishing.^154^ Middle panel: The RISD process. (C) Cartoon of the reorientation induced spectral diffusion for one methanol-d^4^ molecule in solution. Rotation of the molecule changes the projection of the static electric field on the transition dipole (μ), changing the frequency. Also, the contribution of the frequency change to < XXXX> is reduced while the contribution to < XXYY> is increased. (D) Simulated parallel (black squares) and perpendicular (red circles) polarization-weighted FFCF data for various structural spectral diffusion rates, with a fixed orientational diffusion constant (τ_m_ = 30 ps). RISD results in a significant difference in the parallel and perpendicular decays, when structural dynamics is slow (SSD, τ_2_ = 2 ns). For SSD time scales not substantially slower than the reorientation time (150 and 50 ps), the increase in decay rate and difference between polarization configurations is visible, but small. A SSD time of 20 ps (faster than τ_m_) is nearly unaffected by the RISD contribution. Reprinted from P. L. Kramer et al., “Separation of experimental 2D IR frequency-frequency correlation functions into structural and reorientation-induced contributions,” *The Journal of Chemical Physics* **143** (12), 124505 (2015), 10.1063/1.4931402, with the permission of AIP Publishing.^257^ Right panel: Structural long-term IL dynamics. (E) CLS decays for 2-SeCN-Bmim^+^ (red squares) and SeCN^–^ (black circles) in an 1-butyl-3-methylimidazolium IL. Adapted with permission from ref.^129^ Copyright 2017 American Chemical Society.

As an extension of this initial work, Brinzer et al.^154^ studied the solvation of carbon dioxide in ILs and demonstrated that neutral solutes also experienced well-defined IL local environments (Figure 21b). The authors noted that the frequency of the antisymmetric stretch of CO_2_ was sensitive to charge-transfer-induced distortions imposed by the surrounding ions. Using a family of imidazolium-based ILs with varying anions, the authors also observed that the spectral diffusion timescales varied up to an order of magnitude in the different ILs, and they scaled linearly with viscosity, reinforcing the ion cage framework and its dependence on anion identity. Moreover, analysis of inhomogeneous line widths and spectral diffusion in these ILs indicated that CO_2_ remains localized in a transient cage until collective rearrangements in the IL permit its exchange. Finally, Ivanova et al. used CO_2_ to study eutectic mixtures of short-chain pyridinium and pyrrolidinium ILs.^155^ Notably, these eutectic IL mixtures outperformed the conventional 1-hexyl-3-methylimidazolium IL in conductivity without increasing its viscosity. The authors observed that the binary IL mixture exhibited faster spectral diffusion compared to imidazolium-based IL, indicating a more rapid reorganization of the ionic cages. The authors attributed this enhanced microscopic mobility to another important feature of IL design, the absence of bulky hexyl chains in the cations of the pyridinium and pyrrolidinium ILs, which reduced steric hindrance and enabled faster cation rotation, allowing the rapid interchange of the CO_2_ ionic cages.

Early 2DIR and pump–probe studies treated spectral and rotational dynamics as independent variables, as is the case for traditional electrolytes and other systems. However, Fayer’s group revealed an important problem with this interpretation. Kramer et al. demonstrated that in viscous 1-hexyl-3-methylimidazolium IL the local electric field experienced by methanol is effectively static on the rotational timescale, and that the probe reorientation alone leads to frequency fluctuations, which can be observe in the 2DIR spectra (Figure 21c,d).^257,260^ This phenomenon, termed reorientation-induced spectral diffusion (RISD), was demonstrated through polarization-selective IR pump-probe and 2DIR experiments combined with theory. The authors showed that neglecting RISD in spectral diffusion dynamics leads to substantial underestimation of intrinsic structural decorrelation times. The results motivated a revised framework that explicitly separates structural spectral diffusion (SSD) from RISD in the interpretation of IL dynamics. Later, the use of this framework in the analysis of the dynamics of SeCN^–^,^255^ and CO_2_ ^153,160^ in 1-ethyl-3-methylimidazolium IL provided clear evidence of the presence of RISD. Once the authors accounted for rotation induced contributions, the two components of the structural spectral diffusion in the ILs were determined to occur on slower timescales than previously reported.^154,245^ Nevertheless, the local rearrangement of the ion cages remained on the order of several tens of picoseconds.^255^

Using the new framework, the perturbations introduced by alkali cations on 1-ethyl-3-methylimidazolium IL containing SeCN^-^ were examined.^255^ The results closely mirroring earlier observations by Ren et al.^251^ For example, in 1-ethyl-3-methylimidazolium IL containing K^+^, the extracted dynamical timescales remained unchanged, but presented increased long time offsets in both orientational and spectral observables. This behavior was described in terms of a two-ensemble picture, in which a fraction of the probes experience very slow dynamics beyond the experimental window, likely caused by the formation of CIPs, while the majority of the molecules remains solvated by the ionic liquid matrix and retains their characteristic tens of picoseconds dynamics.

These early 2DIR studies using small external vibrational probes were instrumental in establishing a consistent picture of localized caging, structural relaxation, and viscosity scaling in IL. However, as the range of vibrational reporters used to investigate ILs via 2DIR spectroscopy expanded, it became increasingly clear that the observed solvation dynamics were not universal, but strongly depended on the chemical identity, charge, and interactions of the probe chosen to study the IL. For example, Dutta et al. showed that neutral organic azides undergo rapid spectral diffusion comparable to that in aprotic molecular solvents, indicating that they primarily probe relatively weakly constrained, solvent like environments.^239^ In contrast, the azide anion exhibited much slower dynamics due to its incorporation into the ionic charge network of a 1-butyl-3-methylimidazolium IL, reflecting strong, localized ion–ion and hydrogen-bonding interactions that are not captured by bulk viscosity alone.^239^

Comparisons between water and small alcohols demonstrated that the shape and size of the probe can also have an impact on the orientational relaxation and spectral diffusion at long timescales in ILs. Kramer et al. studied the solvation and rotational dynamics of the OD stretches of water, methanol, and ethanol in the 1-ethyl-3-methylimidazolium IL.^161^ The authors observed that, in the IL, all three probes exhibit comparable triexponential decay behavior in both spectral diffusion and orientational relaxation, attributting the subpicosecond and few-picosecond time constants to specific hydrogen-bond length fluctuations occurring in these probes. However, at the timescale for cage reformation, spectral diffusion showed a weak dependence on solute size, while orientational relaxation slowed down appreciably with increasing probe size. This observation demonstrated that the shape and volume of the probe impact reorientation far more severely than spectral diffusion at long times.

The effects of hydrogen-bonding on the IL dynamics and transport properties were also studied with nonlinear and more delocalized anions. For example, Guchhait et al. reported lower viscosity, reduced melting points, and improved ionic conductivity when they incorporated the tricyanomethanide (TCM^–^) anion into a series of imidazolium-based ILs.^100^ TCM^–^ exhibited the two characteristic timescales of spectral diffusion, but at substantially faster rates compared to its linear ionic analogs, indicating shorter ion cage lifetimes. The authors linked the faster dynamics to the delocalized charge of TCM^–^ (i.e., distributed over the CN groups), which weakens the local electrostatic and hydrogen-bonding interactions with imidazolium cations. Surprisingly, the authors also observed that increasing the hydrogen-bonding strength of the IL by adding an OH group in the cation did not measurably affect the spectral diffusion of TCM^–^. The authors explained the phenomenon as a trade-off, in which the acceleration from the hydrogen-bonding was offset by the substantially higher viscosity. This study showcased once more that the relationship between local and bulk structures and dynamics of ILs is not straightforward.

Other studies sought to extend the scope of ultrafast spectroscopic investigations toward intrinsic IL dynamics through the development of site-specific vibrational labels with the aim of minimizing perturbations to the native local structure . For example, the Dutta group introduced a C2 deuteration in the imidazolium cation, as a site-specific and minimally perturbative vibrational probe, to study hydrogen-bonding and the effect of adding solutes, like NO_2_, DMSO, and acetone, to the IL (Figure 22c).^128,252^ The authors observed that the probe was sensitive to strong hydrogen-bond acceptors, like DMSO, due to the formation of stable complexes. In contrast, weaker acceptors, like acetone, formed stacked configurations with the imidazolium cation rather than hydrogen-bonded acceptor motifs. Spectral diffusion of the weakly and solvent mediated hydrogen-bonded species was reported on the picosecond timescale, showcasing the typical hydrogen-bond dynamics. In contrast, the strongly bound cation–anion pairs exhibited much slower (unresolved) dynamics, demonstrating the importance of ion-ion interactions in defining the IL dynamics.

**Figure 22:**
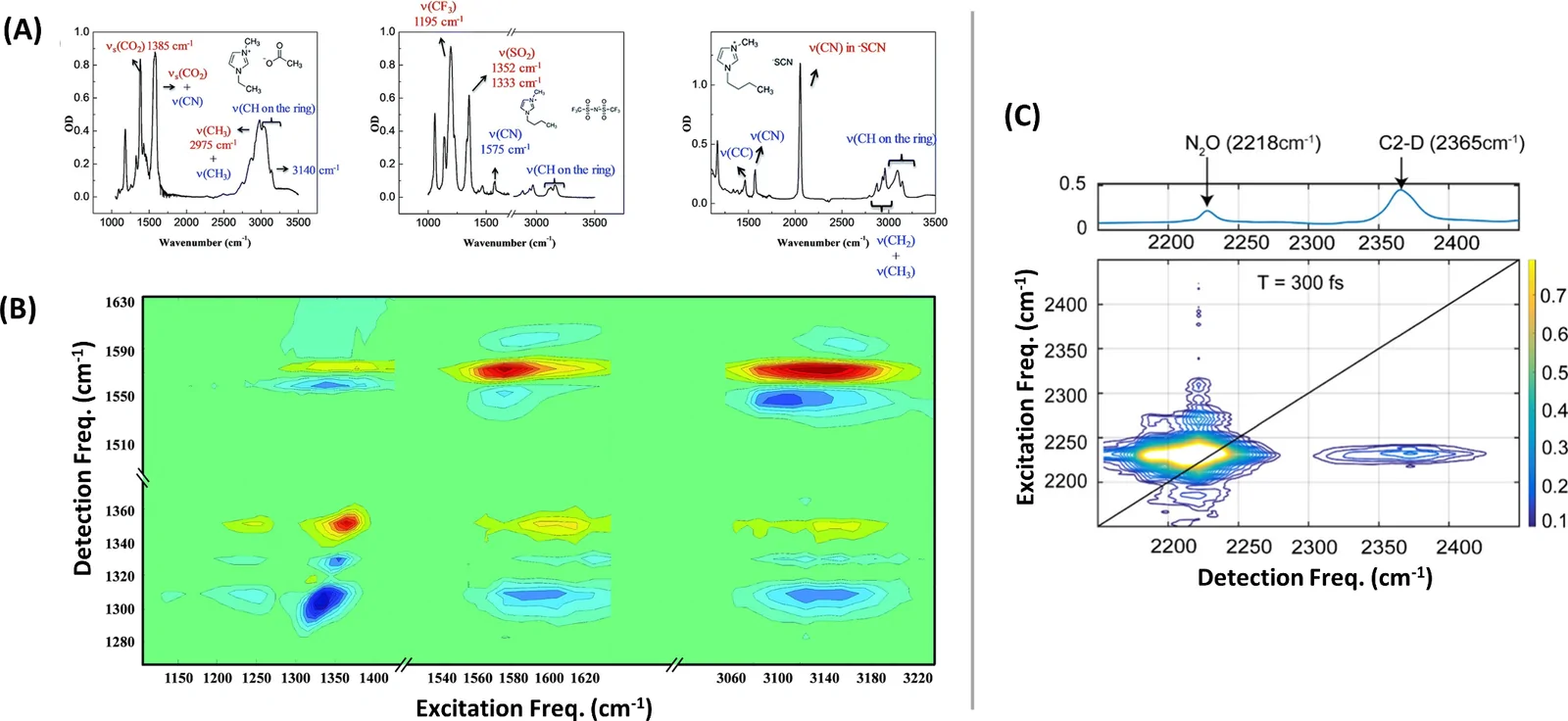
Interaction between IL components and/or solutes and IL. Left panel: Vibrational energy transfer between different modes. (A) FTIR spectra of 1-ethyl-3-methylimidazolium acetate, 1-butyl-3-methylimidazolium bis­(trifluoromethylsulfonyl)­imide, and 1-butyl-3-methylimidazolium thiocyanate. (B) 2DIR spectrum of 1-butyl-3-methylimidazolium bis­(trifluoromethylsulfonyl)­imide at time zero. Adapted with permission from ref.^244^ Available under a CC-BY 3.0 Unported license. Copyright 2018 Chen et al. Right panel: Interaction between IL cation and a solute. (C) Absolute-value 2DIR snapshot of N_2_O dissolved in C_2_-D labeled [EMIM]­[FAP] shows a clear cross peak indicating an interaction between the gas and the cation. Adapted with permission from ref.^128^ Copyright 2019 American Chemical Society.

Despite these advances, the probes discussed above were unable to capture the sub nanosecond timescale for complete bulk structural reorganization in ILs reported by other techniques, such as optical Kerr effect (OKE) spectroscopy.^273,274^ To address this discrepancy, Yamada et al. developed a novel reporter by incorporating the SeCN moiety into the imidazolium ring (Figure 21 e).^129^ This new probe not only closely mimics the original cation, but also, it has sufficiently long vibrational lifetime to probe the subnanosecond timescale. While the two faster spectral diffusion components were consistent with those previously observed using smaller probes, the major advance of this work was the observation of an additional, much slower spectral diffusion component. This newly identified long timescale, of hundreds of picoseconds, in the spectral diffusion dynamics closely matched the complete structural randomization time of the ionic liquid network observed in both pump–probe and OKE measurements. Hence, by directly accessing these collective motions, the cation-based probe reconciled the discrepancy between the local perspective in the IL dynamics, deduced using small vibrational probes and spectroscopic measurements of bulk structural relaxation.

The wide diversity of probe dependent observations in ILs stands in stark contrast to aqueous and organic solvent electrolytes, where solvation dynamics measured by different probes converged to a molecular framework, reflecting the relative homogeneity and rapid structural averaging of the solvent environment. In ILs, the difference in dynamics does not challenge the ion cage framework, but instead, it reveals a pronounced structural and electrostatic heterogeneity, which inevitably leads different probes to selectively sample distinct local motifs and dynamical processes. Thus, a number of studies have focused on establishing a more comprehensive and unified picture of IL structure through dynamical investigations using 2DIR and other time-resolved spectroscopies.

Zheng’s group directly probed the relative orientations of neighboring counterions in different imidazolium-based ILs by selectively exciting vibrational modes of the imidazolium ring and detecting their coupling to vibrational modes of the different anions (Figure 22a,b).^244^ The authors demonstrated that nearest-neighbor cation–anion orientations are effectively random, directly contradicting models based on persistent ion pairing.^51^ Instead, they proposed that ILs lack a well-defined order at the subnanometer length scale, and are more accurately described as forming bicontinuous ionic and nonpolar domains at the mesoscale. In this picture, ILs behave as 3D liquids, composed of interconnected molten-salt-like and molecular-liquid-like domains.

Mukherjee et al. also reported structural inhomogeneity in neat 1-ethyl-3-methylimidazolium trifluoroacetate by directly probing the asymmetric carbonyl stretching mode of the anion, without the use of any external probe.^254^ Although the time evolution of the vibrational signals was not resolved, the linear and time dependent spectra revealed the coexistence of multiple hydrogen-bonded TFA^–^ species within the native IL structure.

Studies on hydrogen-bonding in ILs later extended to protic ILs. Johnson et al. used SCN^–^ as a vibrational probe to study ethylammonium nitrate.^258^ Their 2DIR experiments revealed significant dynamical heterogeneity in the protic IL, characterized by two distinct structural subensembles. The two subensembles exhibited contrasting reorientational dynamics and spectral diffusion, which were assigned to the location of the probe in either nanometer-scale, charge-dense regions (ammonium and nitrate ions) or charge-depleted regions (alkyl chains).

Overall, these 2DIR studies revealed a hierarchical organization, in which dynamically distinct environments coexist within ILs, comprising locally disordered structures embedded in a medium that undergoes slow, collective structural relaxation. These findings further emphasized that probe selection is a decisive factor in determining which structural motifs, intermolecular interactions, and dynamical processes are studied when investigating ILs via 2DIR spectroscopy.

### 6.2 Effect of Alkyl Chain Length

Changes in the long-range dynamical behavior of ILs have been closely linked to variations in the length of the alkyl side chains of the IL cation. As the chain length increases, the growing nonpolar character of the cation promotes segregation between charged (polar) and alkyl (nonpolar) regions in the intrinsically heterogeneous IL environment.^275–277^ Accordingly, this section examines how such structural evolution at the mesoscale manifests in the time-dependent dynamics obtained from spectral diffusion and anisotropic measurements.

Early investigations using carbon dioxide as a neutral probe established that CO_2_ resides almost exclusively within the polar ionic regions of imidazolium-based ILs. Giammanco et al. observed only small red shifts and modest line width changes in the linear IR of the probe when the chain length of the IL cation was increased (Figure 23a).^156^ These observations were consistent with a gradual reduction in the average polarity of the system, but with little change in the structure of the local environment. Rotational and spectral diffusion dynamics revealed that short-time solvation dynamics and restricted wobbling motions of CO_2_ are not sensitive to the IL cation chain length. In contrast, the long-time dynamics, linked to the rearrangement of the ionic cages and displayed by both reorientation and spectral diffusion, slowed down by nearly an order of magnitude when the alkyl chain was increased from 2 to 10 carbon atoms. Notably, the slowest component of structural rearrangement dynamics exhibits a sharp increase between butyl and hexyl chains, which coincides with the rise of nanoscopic ordering as shown by X-ray scattering.^275,277^ The authors therefore concluded that the polar-nonpolar nanosegregation is the dominant factor modulating the IL long-time dynamics.

**Figure 23:**
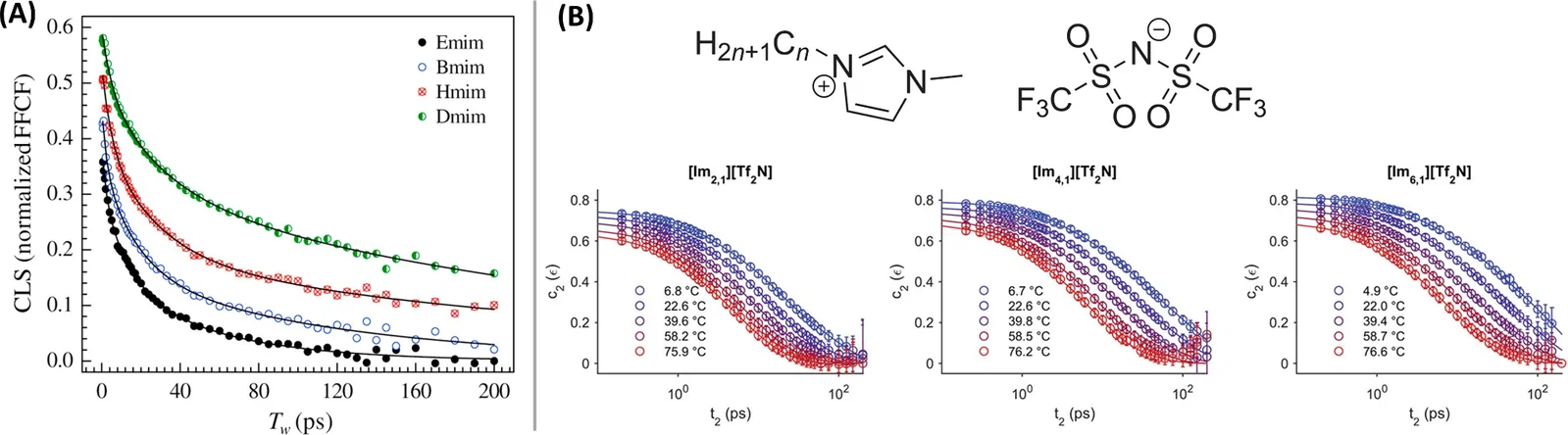
IL dynamics as a function of the cation alkyl chain length. Left panel: Dynamics of CO_2_ in different 1-alkyl-3methyl-imidazolium ILs. (A) Isotropic spectral diffusion of carbon dioxide asymmetric stretch in the RTILs, as quantified by the CLS method. Adapted with permission from ref.^156^ Copyright 2016 American Chemical Society. Right panel: Dynamics of SCN^–^ in different 1-alkyl-3methyl-imidazolium ILs. (B) FFCF for the CN stretch of SCN^–^ with increasing temperature. Colored circles correspond to ellipticities of the 2DIR spectra, while colored lines represent the corresponding triexponential fit. Reprinted from T. Brinzer and S. Garrett-Roe, “Temperature and chain length dependence of ultrafast vibrational dynamics of thiocyanate in alkylimidazolium ionic liquids: A random walk on a rugged energy landscape,” *The Journal of Chemical Physics* **147**, 194501 (2017), 10.1063/1.4991813, with the permission of AIP Publishing.^249^

Building on this work, a complementary perspective was provided in a series of follow-up studies from the same group. These studies used the weakly coordinating SeCN^–^ probe,^250^ as well as the OD stretch^240^ in water-diluted imidazolium-based ILs. Despite the distinct charge, size, and interaction motifs of these two reporters, the authors observed comparable trends with increasing alkyl chain length to those reported for CO_2_.^156^ However, in the case of SeCN^–^ and water, the slowdown of the long-time component of the structural spectral diffusion displayed a much weaker chain-length dependence, changing by approximately a factor of two when transitioning from a cation with an ethyl group to one with a decyl group. This result demonstrated that the mesoscale organization in the IL universally affects the slow dynamical component, but its manifestation strongly depends on how the probe interacts with polar and nonpolar domains.

Further insights into the connection between alkyl chain length and collective dynamics in ILs were provided by temperature-dependent studies of thiocyanate anion dynamics in alkylimidazolium ILs. Brinzer et al. showed that spectral diffusion dynamics increases with temperature, but decreases with increasing chain length, with the structural decorrelation times scaling proportionally to bulk IL viscosity (Figure 23b).^249^ Arrhenius analysis revealed activation barriers for local structural rearrangements in the IL comparable to those governing ion diffusion, but with a nonmonotonic dependence on chain length. This observation showcased the presence of reduced frictional interactions between cation head groups and anions in the longest chain ionic liquid, likely due to weaker intermolecular forces. The authors interpreted the results with a ″rugged energy landscape″ model. In this model, structural rearrangements are thermally activated and progress along low-frequency coordinates, and the solvent friction is determined by local energetic barriers. Hence, this model explained the influence of mesoscopic nonpolar domains on collective transport and structural relaxation processes occurring in ILs with temperature.

In contrast to these general trends, a standalone study by Mora et al. reported no measurable dependence of either vibrational frequency or spectral diffusion times of W­(CO)_6_ on alkyl chain length in a family of fluoroalkylphosphate (FAP) imidazolium-based ILs.^253^ This apparent exception was interpreted as an effect of probe size, polarity, and solvation preferences specific to the FAP IL framework, highlighting the broader theme of probe-dependent sensitivity to IL nanostructure.

Altogether, these studies demonstrated that increasing alkyl chain length selectively influences long-time structural and orientational randomization dynamics through the formation of polar–apolar nanostructures, while short-time local dynamics involving ionic cages remains remarkably insensitive for many different vibrational reporters.

### 6.3 Effect of Water and Hydrogen-Bonding

The addition of water to ILs has been widely explored as a practical strategy to reduce the high viscosity and moderate conductivity of neat ILs at room temperature.^278–280^ Initially, the presence of water was though to weaken the cation–anion interaction. However, 2DIR studies revealed that the structural and dynamical response of IL-water mixture is far more complex than simple dilution would suggest.

Wong et al. first showed that at very low concentrations (χ­(H_2_O) = 0.04), water molecules can remain largely isolated within imidazolium-based ILs, since these ILs primarily interact with the anions through hydrogen bonds with water molecules.^242^ Under these conditions, the spectral diffusion dynamics of water exhibited the same biexponential behavior observed for anionic probes in neat ILs, corresponding to fast local motions and slower ion cage rearrangements.^153,154,160,245,255^ The 2DIR spectra of water also revealed complex vibrational dynamics, including incoherent population transfer between the symmetric and antisymmetric stretching modes. Further, these vibrational transitions presented quantum beats in the form of peak shape oscillations arising from anticorrelated frequency fluctuations in the isolated water molecules. The authors attributed this latter phenomenon to variations in the coupling strength between the local hydroxyl modes due to the ionic IL environment.^242^ These observations suggested that dilute water molecules can largely experience the intrinsic dynamics of the IL matrix.

More recent studies have also revealed that water can act as a strong perturbation in the ionic network. Rather than homogenizing the liquid structure, the addition of water was found to enhance local heterogeneity and promote the emergence of distinct microenvironments. For example, Tibbetts et al. demonstrated that water induces significant heterogeneities in 1-butyl-3-methylimidazolium IL mixtures (Figure 24a).^98^ Using dicyanamide anions (DCA^–^), the authors showed that low, intermediate and high water contents promote water clustering and the formation of distinct anion populations, each characterized with unique solvation structures and dynamical behaviors. Interestingly, while the bulk viscosity decreased monotonically with increasing water content, the spectral diffusion and rotational dynamics of water-associated anions slowed down dramatically. This apparent discrepancy was explained by the authors in terms of water weakening the intermolecular interactions in the IL, which, on a macroscopic scale, resulted in lower viscosities, but simultaneously imposing stronger restrictions on the local solvation cages experienced by DCA^–^. Thus, water emerges not only as a viscosity modifier, but also as a structure disruptor that can stabilize long-lived solvation motifs within a highly dynamic ionic environment.

**Figure 24:**
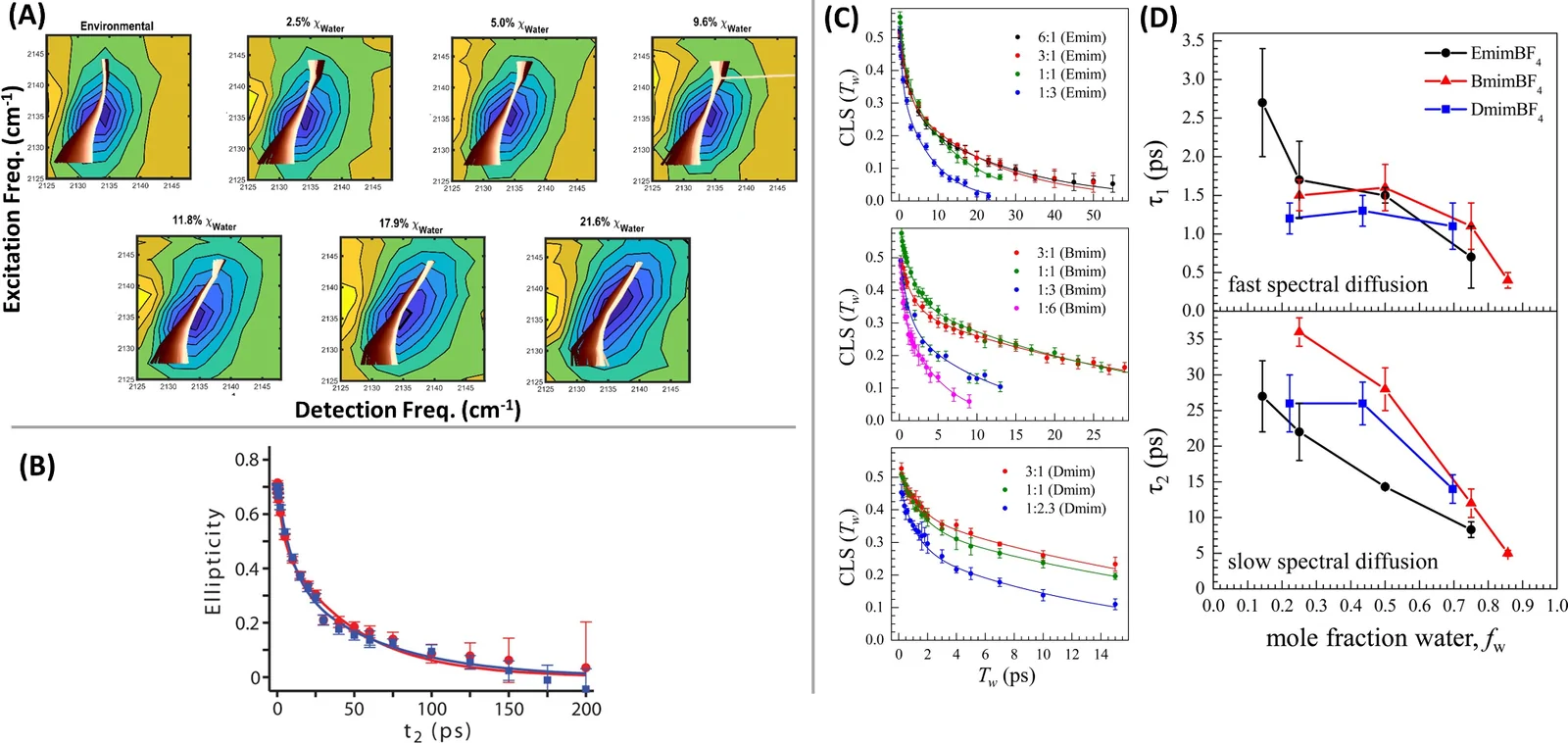
Effect of water on IL dynamics. Left top panel: Dicyanamide anion in IL mixtures. (A) 2DIR spectra with overlaid center line decays for each water sample, from short time (dark red) to long time (yellow) waiting time of 20.4 ps. Adapted with permission from ref.^98^ Copyright 2023 American Chemical Society. Left bottom panel: SCN^–^ in dry and wet ILs. (B) The ellipticity of SCN^–^ dynamics for both systems of dry sample (blue) and water saturated sample (red). Adapted with permission from ref.^251^ Copyright 2015 American Chemical Society. Right panel: Water dynamics in different ILs. (C) CLS decays for the anion-associated hydroxyls in different alkyl chain length ILs. (D) Spectral diffusion time constants (fast, τ_1_, and slow, τ_2_) for OD stretch of HOD in the RTIL solutions as a function of water mole fraction. Adapted with permission from ref.^240^ Copyright 2016 American Chemical Society.

Importantly, the relationship between these microscopic structural changes and bulk properties was observed to depend strongly on the choice of vibrational reporter. Several studies of IL-water mixtures have reported enhanced heterogeneity upon water addition, but the resulting dynamical responses were not universal. In particular, two studies (Figure 24b) used the nitrile stretch of thiocyanate^251^ and the OD stretch of water (Figure 24c,d)^240^ as molecular reporters. As observed for the IL containing DCA^–^,^98^ the introduction of water led to the emergence of two distinct sub ensembles in both the SCN^–^ and OD spectra. One ensemble was assigned to the isolated probe residing within the native IL structure, and the other to the probe having one or more water molecules in their first solvation shell. However, unlike the dynamics perceived by DCA^–^ in the IL, which slowed progressively with increasing water content, the results from SCN^–^ probe did not exhibit significant changes in the long-lived dynamics, whereas the OD stretch reported an increase in fast structural and orientational relaxation dynamics. All together, these results indicate that water does not impose a uniform modification of IL dynamics; instead, its influence is highly localized and manifests as probe dependent dynamical responses that cannot be directly correlated to macroscopic properties, such as viscosity.

The pronounced heterogeneity introduced by water in ILs raises a more fundamental question regarding the extent to which the local environments experienced by water molecules resemble those encountered in conventional aqueous electrolytes. Addressing this issue requires direct comparisons between these two classes of ionic media, enabling the distinct structural roles of water to be studied in both ionic frameworks. In this regard, Giammaco et al. compared water dynamics an aqueous lithium salt solution and a water diluted 1-ethyl-3-methylimidazolium IL, both containing TFSI^–^ as the anion.^186^ The authors observed that water molecules hydrogen-bonded to the anions exhibited similar vibrational lifetimes and rotational dynamics in the two systems, but the spectral diffusion was significantly slower in the lithium salt solution. The authors attributed this difference to the distinct structural roles of water. In the aqueous electrolyte, water acts as an integral component of the lithium-anion coordination network, whereas in the IL, water remains comparatively dynamically unconstrained due to the inherent ionic ordering of the surrounding IL environment.

Another contrasting difference between water-diluted IL and traditional aqueous electrolytes is the timescale of the chemical exchange between water molecules hydrogen-bonded to other water molecules and those hydrogen-bonded to anions. Wei et al. measured the 2DIR spectra of water in a 1-butyl-3-methylimidazolium tetrafluoroborate (BF_4_^–^), and observed population interconversion on the tens of picoseconds timescale,^241^ which is significantly slower than the reported value for NaBF_4_ aqueous solutions.^152^ Further, the reorientational dynamics of water molecules and of the external anionic probe (SCN^–^) both exhibit a slowdown with increasing IL concentration, but at different rates. The authors attributed the distinct rotational behavior of the anion reporter to its localization within cation segregated nanostructures of the IL.

The nanosegregation of solutes via hydrogen-bond formation in ILs extends beyond water-containing systems. Cui et al. studied the transition from imidazolium-based IL to amide-based deep eutectic solvents, with increasing concentrations of the hydrogen-bond donor trifluoroacetamide.^259^ By measuring spectral diffusion of the thiocyanate ion as a function of the amide concentration, the authors observed a change in the ion-cage restructuring dynamics with amide concentration. The authors interpreted this dynamical behavior as a change in the amide solvation environment, from a relatively homogeneous one at low amide concentration, to a nanosegregated one when the amide content reach the critical threshold of 1:1 molar ratio between the amide and the IL. This structural reorganization highlights the general propensity of hydrogen-bonding additives to self-associate within IL environments.

### 6.4 Effect of Confinement and Interfaces

Confinement effects provide a powerful way of separating local solvation dynamics from collective and long-range structural motions in IL. Unlike molecular liquids, spatially constrained ILs, whether confined within colloidal dispersions, nanoporous membranes, thin films, or polymer matrices, exhibit interface-induced ordering that can extend over tens to hundreds of nanometers. This distance far exceeds the few-nanometer length scales typical of simple liquids.^281–283^ As a result, the confinement effects on the structural rearrangement dynamics offer an ideal platform for disentangling local and collective processes in ILs.

Initial insights into confinement-induced dynamics were provided by Ren et al.^243^ The authors used 2DIR spectroscopy to probe 1-buthyl-3-methyl imidazolium IL dynamics within microconfined systems formed using various organic solvents and surfactants. Their results revealed pronounced variations in the reorientational and structural dynamics perceived by SCN^–^ with solvent identity, viscosity, and surfactant chemistry used to create the micelle-like system. These findings support the view that surfactant addition promotes the formation of interfacial microscopic complexes, in which the IL is in direct contact with the organic solvent, rather than forming reverse micelles where the IL is isolated in its core, as seen in water.^284–286^

A systematic evaluation of confinement effects in ILs was achieved using supported IL membranes (SILMs). To this end, Shin et al. studied the picosecond vibrational spectroscopy of SeCN^–^ in immobilized 1-ethyl-3-methylimidazolium bis­(trifluoromethylsulfonyl)­imide in nanoporous polymer matrices with pore sizes varying from 100 to 300 nm.^256^ The authors observed that confinement slowed down both IL orientational relaxation and structural spectral diffusion when compared to the IL bulk. Interestingly, the linear IR spectra and vibrational lifetimes demonstrated that the local environment of the probe did not change within the membrane. These two observations led the authors to conclude that polymer interfaces impose long-range structural ordering on IL. Moreover, the dynamical perturbations were observed to propagate over distances exceeding 100 nm, which is orders of magnitude larger than that observed for nanoconfined molecular liquids.^284–287^ To test the generality of this conclusion, a subsequent study by the same authors used CO_2_ as a neutral probe to study the 1-ethyl-3-methylimidazolium bis­(trifluoromethylsulfonyl)­imide confinement.^157^ While confirming the overall slowing of collective dynamics of the IL under confinement, this work also revealed a clear probe dependence. Within the confined membrane, CO_2_ occupied two distinct locations. One corresponding to the IL-filled pores, and the other trapped in polymer matrix itself. As in the previous case, the probe located in IL-filled pores sensed similar local structures relative to the bulk, but orientational and structural randomization were slow down by a factor of 2, despite the relatively large 350 nm average pore size of the membrane. Together, these results showcased further evidence that IL confinement primarily perturbs its collective dynamics, while leaving local IL solvation environments largely unperturbed, regardless of probe identity.

The influence of confinement was further explored in more realistic operating conditions by examining how water affects CO_2_ dynamics in SILMs. Shin et al. showed that the presence of water substantially decreases the population of CO_2_ in the IL-pore phase and increases its population in the polymer matrix.^158^ Analysis of orientational and structural dynamics showed that water enhances large scale fluctuations of the IL in the SILM more strongly than in the bulk, yet it has minimal impact on the IL environment surrounding CO_2_. These results indicated that the long-range ordering induced by the polymer pore interface persists even in the presence of water. However, they also showed that the addition of water is unlikely to improve CO_2_ translational diffusion, or equivalent solubility, because the local dynamics that govern it remains largely unaffected. These observations were consistent with earlier findings by Scovazzo et al. from CO_2_ sorption isotherms.^288^

An even more pronounced manifestation of confinement effects emerges when ILs are constrained to thin films. Nishida et al. investigated 1-butyl-3-methylimidazolium bis­(trifluoromethylsulfonyl)­imide films with thicknesses ranging from tens to hundreds of nanometers (Figure 25a,b).^247^ Despite the absence of detectable changes in linear vibrational spectra, the authors observed an extraordinary slowing of structural dynamics as film thickness decreased. Specifically, spectral diffusion correlation times increased by four times from the bulk liquid to the thickest films, and by a factor of 4 in the thinnest film. They further estimated interfacial correlation lengths of approximately 20–30 nm, indicating that Coulombic interactions propagate the IL ordering created by the interface far beyond the few nanometers of the interfacial region.

**Figure 25:**
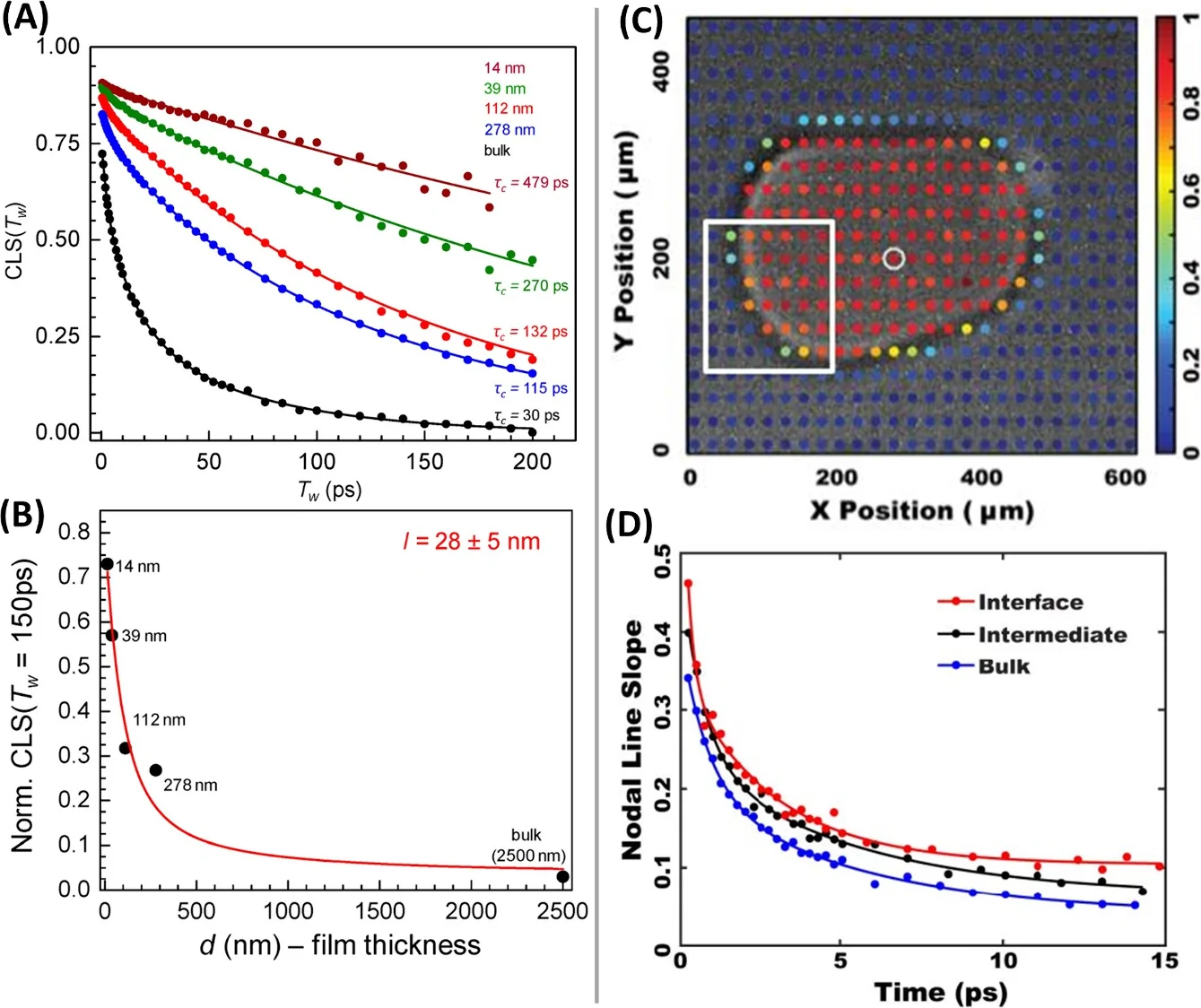
Confinement effect in IL dynamics. Left panel: ILs in thin films. (A) CLS decays extracted from 2DIR band shapes as a function of waiting time and for films with different thicknesses (14, 39, 112, and 278 nm, and bulk). Dots are averaged data and lines fits to multiexponential decays. (B) Normalized CLS values at *T* = 150 ps plotted vs the film thickness. Adapted with permission from ref.^247^ Copyright 2018 American Chemical Society. Right panel: 2DIR microscopy of an IL drop in oil. (C) Image of the RTIL microdroplet generated from the 2DIR imaging experiments with white box indicating the points chosen for the 2DIR experiments. (D) NLS curves extracted from points in the interfacial (blue), in the intermediate (black) and bulk (blue) regions. The points are the experimentally measured values and the lines indicate the biexponential fits to the data. Reproduced from ref.^122^ Available under a CC BY-NC-ND license. Copyright 2019 Tracy et al.

Complementary insights into film-induced ordering were provided by Wu et al., who showed that the extent of interfacial influence on imidazolium-based IL thin film dynamics depends strongly on the alkyl chain length of the cation.^248^ In this study, the authors noted that, as the alkyl chain length in the IL cation increases, the influence of interface on the dynamics weakens more rapidly with distance. The authors attributed this behavior to nanosegregation in long-alkyl-chain ILs, which promotes bulk-like organization close to the interface and disrupts interfacial ordering.

Beyond thickness effects, the role of surface chemistry on the dynamics of ILs was also explored by systematically varying the surface charge density through changes in the density of surface-functionalized ionic groups.^246^ Wu et al. showed that increasing surface charge density leads to progressively slower dynamics in the imidazolium-based IL, particularly in thinner IL films, demonstrating that electrostatic interactions at interfaces exert strong control over IL structural ordering and relaxation. Notably, even IL films in contact with nominally neutral surfaces exhibited slower structural reorganization dynamics than the bulk IL, underscoring the intrinsic sensitivity of ILs to the chemical environment of the interface.

A further advance in probing confinement-induced heterogeneity was introduced by Tracy et al., who developed a spatially resolved 2DIR microscopy approach to directly image dynamics within individual IL microdroplets (Figure 25c,d).^122^ In this work, microdroplets of 1-ethyl-3-methylimidazolium tetrafluoroborate containing the vibrational probe tricyanomethanide (TCM^–^) were suspended in oil, enabling the first spatial maps of solvation dynamics with micrometer resolution. The authors identified three dynamically distinct regions within a single droplet: a bulk-like interior, a highly perturbed interfacial layer, and an intermediate transition region. Although interfacial dynamics were faster than in the droplet interior, they were also more spatially constrained. In contrast, the intermediate region exhibited mixed dynamical behavior showcasing the gradual crossover between the bulk and the interface. Models of the spatially-resolved dynamics in the microdroplet indicated that interfacial perturbations extend over tens of micrometers, demonstrating that confinement and interfaces can influence IL dynamics far deeper into the IL than previously anticipated.

Finally, Kelsheimer et al. investigated confinement effects in ion gels by probing the CO_2_ solvation dynamics in three distinct systems: a neat imidazolium-based ionic liquid, a cross-linked poly­(ethylene glycol) diacrylate (PEGDA) polymer gel, and a 50:50 mixture of the two forming a gel.^159^ In the hybrid system, the overall structural reorganization timescales were found to be in between those of the pure IL and the pure polymer, demonstrating a systematic slowing of collective dynamics with increasing polymer content. In contrast, intramolecular vibrational energy relaxation of CO_2_ in the IL gel closely resembled that of the neat IL, as evidenced by comparable energy-transfer kinetics and the absence of a hot-ground-state feature, observed only in the pure polymer matrix. The apparent decoupling between local and collective dynamics was attributed to CO_2_ preferentially residing in IL-rich regions near the polymer interface, where the local solvation environment remains IL-like. However, it was determined that the long-range structural relaxation of IL gel is also governed by the surrounding polymer network. Together, these results reinforce the view that confinement selectively perturbs collective, low-frequency motions while largely preserving short-range solvation dynamics in ILs.

## 7 Emerging Molecular Principles from 2DIR Studies of Electrolytes

A broad consensus has emerged regarding the characteristic time scales and molecular origins of the dominant dynamical processes in aqueous electrolytes, organic electrolytes, and ionic liquids. Across these systems, local structural fluctuations, chemical exchange events, and collective reorganization processes occur on distinct time scales, providing a unified framework for describing electrolyte dynamics at the molecular level.

Figure 26 summarizes the dynamical pictures that have emerged from three decades of 2DIR experiments and related ultrafast spectroscopic studies of aqueous electrolytes, organic electrolytes, and ionic liquids. This framework has been shaped by experimental considerations, particularly the limited availability of suitable vibrational reporters and the inherent complexity of 2DIR spectral interpretation. Selecting the appropriate probes has required balancing chemical relevance with spectroscopic requirements, such as vibrational lifetime and spectral isolation. These limitations have motivated the design and implementation of tailored vibrational reporters. Together, these advances have enabled the reliable separation of overlapping contributions happening at similar timescales, thereby refining mechanistic interpretations of electrolyte dynamics.

**Figure 26:**
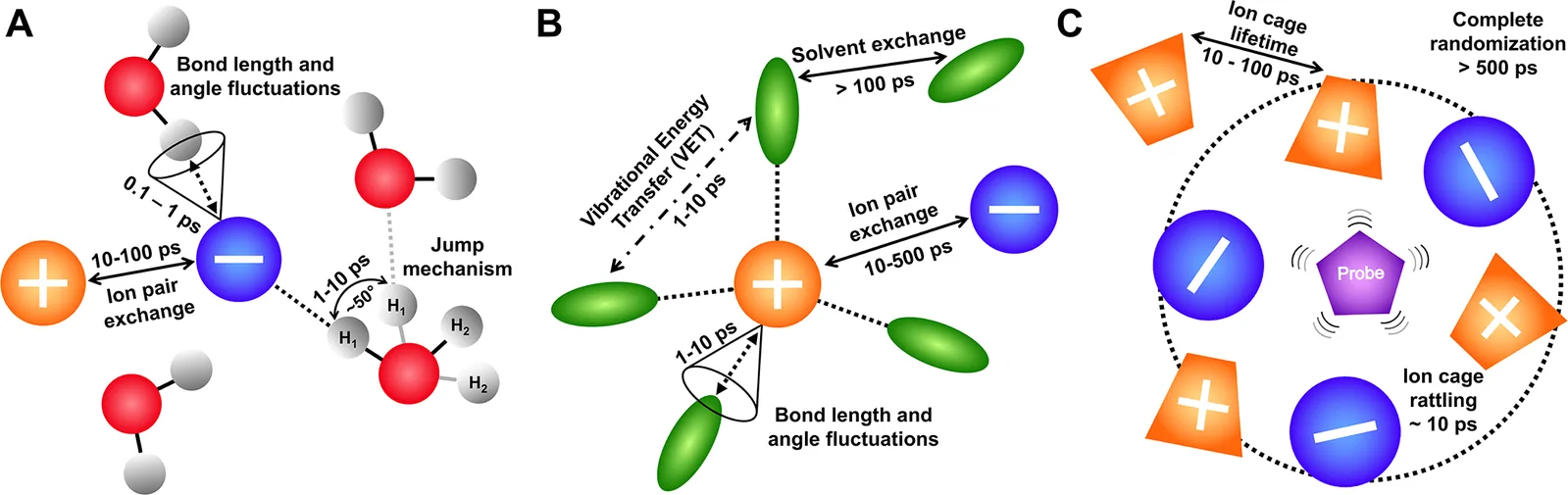
Cartoon representation of solvation dynamics and time scales identified by 2DIR spectroscopy in different electrolytes. (A) Aqueous anion solvation shells, dominated by bond/angle fluctuations, the water jump mechanism, and speciation exchange. (B) Organic electrolyte solvation shells (e.g., Li^+^) in tetrahedral coordination arrangement with VET among bound solvent molecules, and slow solvent exchange. (C) Ionic liquid cage structure around a vibrational probe, showing the process and time scale of local rattling, cage lifetime, and complete randomization.

In aqueous electrolytes (Figure 26a), local fluctuations of hydrogen-bond lengths and angles occur on subpicosecond to picosecond timescales, while hydrogen-bond exchange around ions proceeds through the well-established jump mechanism, in which reorientation occurs via concerted, nondiffusive motions.^216,217^

Organic electrolytes (Figure 26b), most extensively studied for Li^+^-containing systems, exhibit a similar separation between local and collective dynamics. The first solvation shell is generally described as tetrahedrally coordinated, and local structural fluctuations, together with vibrational energy transfer (VET) among coordinated solvent molecules occur on the 1–10 ps timescale. Unlike aqueous systems, solvent exchange proceeds through a diffusive pathway, requiring partial disruption of the primary coordination environment, and therefore occurs on substantially longer timescales (>100 ps). Despite the slow solvent exchange dynamics, CIP formation and dissociation in organic electrolytes have similar timescales as in aqueous electrolytes.

Ionic liquids (Figure 26c) exhibit a distinct structural picture in which neighboring ions form transient ionic cages surrounding the probe. Without considering systems with hydrogen-bonding motifs, these cages lack persistent directional order and are instead characterized by dynamically disordered ion arrangements. Local structural fluctuations are commonly described as cage rattling, occurring on ∼ 10 ps timescales, whereas the lifetime of a given cage configuration is typically 10–100 ps, comparable to the timescale associated with contact ion-pair exchange in electrolytes. Complete configurational randomization occurs in substantially longer timescale (>500 ps), again reflecting the separation between local structural fluctuations and slower collective reorganization observed across all three classes of systems. Collectively, these observations establish a molecular framework in which distinct dynamical regimes can be assigned to local solvation motions, solvent or ion exchange events, and long-range structural relaxation, largely independent of the specific identities of the constituent ions.

Beyond the identification of characteristic timescales, 2DIR studies have also revealed several broader chemical principles. First, electrolyte properties emerge from continuously exchanging local environments, rather than static solvation structures. Second, increasing salt concentration promotes a transition from solvent-dominated behavior toward collective ionic networks, producing dynamics that increasingly resemble those observed in ionic liquids. Third, local structural fluctuations and macroscopic transport properties are often only weakly correlated, indicating that macroscopic properties, such as viscosity and conductivity, are not generally predicted from a single microscopic timescale. Finally, the observed dynamics depend strongly on the local coordination environment, where the role of ion pairing, aggregation, and solvation-shell composition are the determining factors of electrolyte behavior.

## 8 Future Outlook

Based on the molecular principles described in the previous section, several important directions emerge for further advancing the field. One key area of ongoing investigation is the mechanistic description of solvent exchange processes. While solvent exchange in aqueous electrolytes is now largely understood within the framework of the water jump mechanism, developing an equivalent, molecular-level description for organic electrolytes remains an important objective. The greater structural diversity and weaker hydrogen-bonding interactions of organic solvents are expected to produce more heterogeneous exchange pathways that have yet to be systematically characterized. Similarly, the mechanism of contact ion pair (CIP) exchange is not yet fully resolved. While some results support a dissociative pathway involving a lower-coordinate CIP intermediate, others suggest associative mechanisms, in which a bonded anion is replaced by a new one simultaneously.^204^ Discerning between these pathways will require continued advances in experimental sensitivity and complementary theoretical approaches, ultimately enabling a more comprehensive, molecular-level description of electrolyte dynamics.

Further progress in this field relies on strengthening the connections between molecular descriptors, electrolyte dynamics, and macroscopic properties. While it is well-known that polar organic solvents stabilize ionic species, establishing quantitative relationships between physicochemical descriptors and dynamical timescales remains an open challenge. Comparisons across studies show that commonly used macroscopic parameters, such as the dielectric constant, do not consistently capture variations in processes such as contact ion pair (CIP) exchange or solvent rearrangement dynamics. For example, solvents with similar dielectric properties can exhibit orders of magnitude differences in CIP exchange rates,^178,184,261^ while systems with comparable exchange kinetics may display markedly different solvent dynamics.^183^ These observations underscore the need for more refined molecular-level descriptors that explicitly account for ion–solvent and ion–ion interactions, as well as structural features such as molecular size, shape, and local organization.

Translating these microscopic dynamics into macroscopic observables is a growing direction for future research, particularly through integrative approaches, such as those combining 2DIR spectroscopy with complementary experimental and theoretical methods. Studies of ionic liquids have provided a relatively consistent picture, where increases in ionic cage lifetimes correlate with higher macroscopic viscosity and also depend on anion identity and alkyl chain length.^100,154,245,249^ In most electrolyte systems, however, such relationships remain less explored, and variations in ultrafast solvation dynamics do not always directly correlate with viscosity or ionic conductivity, even at high salt concentrations^140^ or under confinement.^182^ Only a limited number of studies in lithium/carbonate electrolytes have provided direct evidence linking ion–ion rearrangements to transport,^188,192^ where vehicular diffusion of cations has been reported to be inversely related to viscosity.^188^

In this context, the role of bulk-like solvent subensembles remains an emerging area of interest. While most studies have focused on solvent molecules directly coordinated to ions, some results have indicated that even the “free” solvent populations can exhibit slower dynamics than the bulk solvent.^85,199,221,263^ Understanding how these subensembles contribute to collective fluctuations, exchange processes, and long-range transport could represent an important opportunity to bridge the gap between local solvation dynamics and macroscopic behavior. Further, this direction will ultimately enable the predictive, mechanism-driven design of next-generation electrolytes.

Another promising direction is extending these studies to more realistic operating conditions. Most investigations to date have focused on simplified systems under ambient conditions, whereas practical energy storage devices involve more complex environments. Some studies have begun to explore multicomponent systems,^114,182,188,196,206,221,223^ including the effects of applied voltage,^177,265^ and temperature variations,^92,213,249^ yet a broader exploration of these conditions could benefit the field. For example, aqueous electrolytes appear to exhibit weak temperature dependence in frequency fluctuation dynamics,^92,213^ but it remains unclear whether this behavior is universal. Systematic investigations across broader compositional and environmental spaces could therefore help determine how external stimuli influence structural relaxation and chemical exchange, and whether new dynamical regimes emerge under extreme operating conditions.

Deep eutectic solvents (DESs) have also emerged as promising electrolyte materials due to their low volatility, high thermal stability, tunable physicochemical properties, and potential for sustainable, low-cost synthesis. However, despite growing interest in their application to energy storage, molecular-level understanding of their structure and dynamics remains limited. Expanding the use of 2DIR spectroscopy to DESs with possible electrolyte applications could provide valuable insights into their complex solvation environments, hydrogen-bonding networks, and ion transport mechanisms. The resulting molecular framework could be used to determine the structure–dynamics–property relationships needed for their rational design.

Overall, these directions emphasize the ongoing evolution of the electrolyte field from establishing fundamental dynamical frameworks to developing a predictive understanding. Addressing these questions is essential for advancing the rational design of electrolytes for their potential applications.

## Supplementary Material



## Notes

Comprehensive versions of Tables 1–5, providing complementary information on the identity of the vibrational modes used for 2D IR measurements and the location of the probe within the electrolytes and ionic liquids studied, distinguishing between intrinsic molecular components and externally introduced vibrational probes (XLSX)

## Abbreviations

Acronym/Abbreviation: Full Term
2D NMR: Two-Dimensional Nuclear Magnetic Resonance
2DIR: Two-Dimensional Infrared (Spectroscopy)
2DA: 2DIR anisotropy
AGG/AGGs: Aggregate(s)
ATR: Attenuated Total Reflectance
BC: 1,2-butylene carbonate
BETI^–^: bis­(perfluoroethylsulfonyl)­imide
BMIm^+^: 1-butyl-3-methylimidazolium
BMMIm^+^: 1-butyl-2,3-dimethylimidazolium
C_2_O_4_^2–^: Oxalate
CX: Chemical Exchange
CIP/CIPs: Contact Ion Pair(s)
CLS: Center Line Slope
DCA^–^: Dicyanamide
DEC: Diethyl carbonate
DGdm^+^: Deuterated guanidinium
DMC: Dimethyl carbonate
DMF: Dimethylformamide
DMI: 1,3-dimethyl-2-imidazolidinone
DMIm^+^: 1-decyl-3-methylimidazolium
DMSO: Dimethyl sulfoxide
EC: Ethylene carbonate
EMC: Ethyl methyl carbonate
EMIm^+^: 1-ethyl-3-methylimidazolium
ESA: Excited-State Absorption
EtAc^–^: Ethyl acetate
EtNH_3_^+^: Ethylammonium
FA: Formamide
FAP^–^: tris­(pentafluoroethyl) trifluorophosphate
FFCF: Frequency-Frequency Correlation Function
FSI^–^: bis­(fluorosulfonyl)­imide
FTFSI^–^: Fluorosulfonyl)­(trifluoromethanesulfonyl)­imide
FTIR: Fourier-Transform Infrared Spectroscopy
Gdm^+^: guanidinium
GSB: Ground-State Bleach
HCE/HCEs: High-Concentration Electrolyte(s)
HFE: 1,1,2,2-tetrafluoro-2,2,2-trifluoroethoxy ethane
HMIm^+^: 1-Hexyl-3-methylimidazolium
IL/ILs: Ionic liquid(s)
IR: Infrared
IR PP: Infrared Pump–Probe (spectroscopy)
MD: Molecular Dynamics simulations
MeOH: Methanol
MMPyrr: 1,1-dimethylpyrrolidinium
MP: Methyl propionate
MPTE: Molecular Photothermal Effect
Mpyr: 1-methylpyridinium
NLS: Nodal Line Slope
NMF: *N*-Methylformamide
NMR: Nuclear Magnetic Resonance
OKE: Optical Kerr Effect (spectroscopy)
OMIm^+^: 1-octyl-3-methylimidazolium
PAN: Polyacrylonitrile
PC: Propylene carbonate
PPC: Poly­(propylene carbonate)
PhSeCN: Phenyl Selenocyanate
RISD: Reorientation-Induced Spectral Diffusion
RTIL/*RT*ILs: Room-Temperature Ionic Liquid(s)
SE: Stimulated Emission
SD: Solvation Dynamics
SE-2DIR: Surface-Enhanced 2DIR Spectroscopy
SILs: Solvate Ionic Liquids
SSD: Structural Spectral Diffusion
SSIP/SSIPs: Solvent-Separated Ion Pair(s)
TCM^–^: Tricyanomethanide
TDI^–^: 4,5-dicyano-2-(trifluoromethyl)­imidazolate
TEU: Tetraethylurea
TFA^–^: Trifluoroacetate
TfO^–^: Triflate (trifluoromethanesulfonate)
TFSI-: bis­(trifluoromethanesulfonyl)­imide
THF: Tetrahydrofuran
TMU: Tetramethylurea
TTE: 1,1,2,2-Tetrafluoroethyl ether
Tw: Waiting Time
VC: Vibrational Coupling
VET: Vibrational Energy Transfer
WISE: Water-in-Salt Electrolyte
